# Functional Characterization of 17 Protein Serine/Threonine Phosphatases in *Toxoplasma gondii* Using CRISPR-Cas9 System

**DOI:** 10.3389/fcell.2021.738794

**Published:** 2022-01-10

**Authors:** Qin-Li Liang, Lan-Bi Nie, Ting-Ting Li, Hany M. Elsheikha, Li-Xiu Sun, Zhi-Wei Zhang, Dan-Yu Zhao, Xing-Quan Zhu, Jin-Lei Wang

**Affiliations:** ^1^ State Key Laboratory of Veterinary Etiological Biology, Key Laboratory of Veterinary Parasitology of Gansu Province, Lanzhou Veterinary Research Institute, Chinese Academy of Agricultural Sciences, Lanzhou, China; ^2^ Faculty of Medicine and Health Sciences, School of Veterinary Medicine and Science, University of Nottingham, Loughborough, United Kingdom; ^3^ College of Veterinary Medicine, Shanxi Agricultural University, Taigu, China; ^4^ Key Laboratory of Veterinary Public Health of Higher Education of Yunnan Province, College of Veterinary Medicine, Yunnan Agricultural University, Kunming, China

**Keywords:** *Toxoplasma gondii*, protein serine/threonine phosphatases, PP7, subcellular localization, virulence, host-pathogen interaction

## Abstract

Protein serine/threonine phosphatases (PSPs), found in various plants and protozoa, are involved in the regulation of various biological processes. However, very little is known about the role of PSPs in the pathogenicity of the apicomplexan protozoan *Toxoplasma gondii*. Herein, the subcellular localization of 17 PSPs (PP5, PP7, EFPP, SLP, PPM3F, PPM4, PPM5A, PPM5B, PPM6, PPM8, PPM9, PPM12, PPM14, PPM18, CTD1, CTD2, and CTD3) was examined by 6× HA tagging of endogenous genes in C-terminal. The PSPs were detected in the cytoplasm (PP5, EFPP, PPM8, and CTD2), dense granules (SLP), nucleus (PPM4 and PPM9), inner membrane complex (PPM12), basal complex (CTD3), and apical pole (PP7). The remaining PSPs exhibited low or undetectable level of expression. To characterize the contribution of these genes to the infectivity of *T. gondii*, knock-out (KO) strains of type I RH strain deficient in the 17 *psp* genes and KO type II Pru strain deficient in *pp7* and *slp* genes were constructed. The pathogenicity of individual RHΔ*psp* mutants was characterized *in vitro* using plaque, egress, and intracellular replication assays, and mouse infection, while pathogenicity of PruΔ*pp7* and PruΔ*slp* mutant strains was evaluated by examining the parasite lytic cycle *in vitro* and assessment of brain cyst burden in mice. No significant differences were observed between 16 RHΔ*psp* strains and wild-type (WT) RH strain. However, RHΔ*pp7* exhibited significantly lower invasion efficiency and parasitophorous vacuole formation *in vitro*, and less virulence in mice compared with other RHΔ*psp* and WT strains. In addition, PruΔ*pp7* exhibited marked attenuation of virulence and significant reduction in the brain cyst burden in mice compared with PruΔ*slp* and WT strains, suggesting the key role of PP7 in the virulence of *T. gondii*. Comparative transcriptomic profiling of the 17 *psp* gene*s* showed that they may play different roles in the pathogenesis of different genotypes or life cycle stages of *T. gondii*. These findings provide new insight into the role of PSPs in the pathogenesis of *T. gondii*.

## Introduction

The causative agent of toxoplasmosis, *Toxoplasma gondii*, is an obligate intracellular protozoan parasite (Robert-Gangneux and Dardé, 2012; Smith et al., 2021), which can infect many warm-blooded animals and humans (Elsheikha et al., 2021). Humans acquire infection *via* waterborne transmission, foodborne transmission, and congenital (transplacental) infection (Yarovinsky, 2014). *T. gondii* infection in immunocompetent individuals generally do not lead to illness; however, infection of immunocompromised individuals such as those with AIDS or malignancies, can lead to encephalitis and retinochoroiditis or even death (Wang et al., 2017). Congenital infection of the fetus can cause neonatal blindness and cognitive impairment (Wang et al., 2017; Elsheikha et al., 2021). Current anti-*Toxoplasma* medications are not highly effective and often cause adverse effects (Holland and Joseph, 2017). Additionally, there remains no vaccine effective for the treatment or prevention of *T. gondii* infection. Therefore, there is a need for the identification of new pharmacological targets for the development of novel therapeutics for toxoplasmosis.

*T. gondii* is exquisitely adapted to invade and colonize host cells and utilizes virulence factors secreted by the specialized organelles, micronemes, rhoptries, and dense granules, to colonize the host cell cytoplasm, proliferate within a parasitophorous vacuole (PV), and egress from the host cell to infect new cells and repeat this lytic cycle (Lebrun et al., 2014). There are several atypical cyclins and cyclin-dependent kinases (CDKs)-related kinases (CRKs) in *T. gondii*, suggesting that phosphorylation events play a role in the cell cycle of the parasite (Gubbels et al., 2008; Alvarez and Suvorova, 2017). Among these, calcium dependent phosphorylation, which is regulated by calcium dependent protein kinases (CDPKs), plays prominent roles in the apicomplexan protozoa (Lourido et al., 2010; Uboldi et al., 2015). *T. gondii* encodes at least 14 CDPKs, most of which play a role in the infection cycle, including invasion, motility, colonization, and egress (Long et al., 2016; Wang et al., 2018). For example, CDPK1 is involved in a signaling pathway that contributes to the motility, invasion, and egress of *T. gondii* (Lourido et al., 2010). Deletion of *cdpk2* causes abnormal accumulation of amylopectin granules in the tachyzoites and renders *T. gondii* Pru strain unable to establish chronic infection in mice (Uboldi et al., 2015; Wang et al., 2018). CDPK3 changes the phosphorylation state of the motor protein myosin A (MyoA) and stimulates the rapid egress of the parasite (Garrison et al., 2012).

Reversible phosphorylation mainly occurs in serine, threonine, and tyrosine, which affects the lytic cycle and pathogenicity of *T. gondii via* regulating the structure and function of proteins (Cieśla et al., 2011; Yang and Arrizabalaga, 2017). Protein serine/threonine phosphatases (PSPs) are a major category of phosphorylation phosphatases, which include three major families: phosphoprotein phosphatases (PPPs), Mg^2+/^Mn^2+^-dependent protein phosphatases (PPMs), and aspartate-based phosphatases (FCP/SCP) (Shi, 2009; Yang and Arrizabalaga, 2017). *T. gondii* contains the complete set of all PPP subfamilies (protein phosphotase 1 (PP1), PP2A, PP2B (a.k.a. calcineurin), PP4, PP5, PP6, and PP7) (Yang and Arrizabalaga, 2017). In addition, the apicomplexan parasites have its unique kelch-like domain containing protein phosphatase (PPKL) subfamily, bacterial-like phosphatase subfamily (Shewanella-like phosphatase, SLP) and EF-hand motif (EFPP) of phosphatase subfamily in the PPPs family (Kutuzov and Andreeva, 2008; Philip et al., 2012; Kerk et al., 2013).

The functions of TgPP1 and TgPP2B have been reported. Exposure to PP1 inhibitors reduces the invasion of *T. gondii*, and PP2B is involved in the attachment and invasion of *T. gondii* (Daher et al., 2007; Paul et al., 2015). PP2C protein phosphatases are the main subfamily of PPMs which requires Mg^2+/^Mn^2+^ phosphatase activity. In contrast to PPPs, PPMs do not have catalytic and regulatory subunits, but a single polypeptide monomer, and there is limited similarity between the two families at the amino acid level (Yang and Arrizabalaga, 2017). PP2Chn is the first member of PPMs family to be studied, and *T. gondii* lacking PP2Chn has limited growth ability *in vitro* (Gilbert et al., 2007). Little is known about the roles of aspartate-based phosphatases in *T. gondii*, which have eight hypothetical FCP/SCP family phosphatases.

To improve the understanding of the role of *psp* genes in the pathogenesis of *T. gondii*, the present study was conducted to elucidate the biological functions of 17 *psp* genes (*pp5*, *pp7*, *efpp*, *slp*, *ppm3f*, *ppm4*, *ppm5a*, *ppm5b*, *ppm6*, *ppm8*, *ppm9*, *ppm12*, *ppm14*, *ppm18*, *ctdspl1*, ctdspl2, and ctdspl3 (abbr. *ctd1*, *ctd2* and *ctd3*) in *T. gondii* type I RH strain and two *psp* genes (*pp7* and *slp*) in type II Pru strain. Although these 17 *psp* genes have phenotype values that ranged from −0.81 to 1.75 (Sidik et al., 2016), which may be dispensable for the growth of parasites *in vitro*, it remains unclear if deletion of these genes can directly affect the pathogenicity *in vitro* and *in vivo* of the parasite. The present study was performed to elucidate the role of these genes in the fitness of *T. gondii*. Our data suggest that one of these *psp* genes plays an indispensable role in the growth capability and virulence of *T. gondii*.

## Materials and methods

### Mice

Female Kunming mice (7–9 weeks old) were obtained from the Center of Laboratory Animals of Lanzhou Veterinary Research Institute, Chinese Academy of Agricultural Sciences. The mice were housed under specific pathogen-free and environmentally enriched conditions with 12-h light/dark cycle and free access to commercial pelleted food and water. The mice were adapted to the environment for at least 1 week prior to the conduction of the experiment (Wang et al., 2020b). The study protocol was reviewed and approved by the research ethics committee of Lanzhou Veterinary Research Institute (Permit No. 2021-002). All the experiments were carried out in accordance with the approved guidelines.

### Parasite culture and purification

The tachyzoites of *T. gondii* strains (type I: RHΔ*ku80* referred as RH and type II: Pru) were grown in monolayers of human foreskin fibroblasts (HFFs, ATCC, Manassas, VA, USA) as described previously (Cao et al., 2019; Wang et al., 2020a). The parasite culture was maintained in Dulbecco’s modified Eagle medium (DMEM) containing 2% fetal bovine serum (FBS), 10 mM HEPES (pH 7.2), 100 U ml^−1^ penicillin, and 100 μg ml^−1^ streptomycin at 37°C and 5% CO_2_. To purify the tachyzoites, infected HFF monolayers that have shown >80% destruction with many extracellular tachyzoites were scraped off and the remaining infected HFF cells were lysed by passing through a 27-gauge needle. The purified tachyzoites were harvested by using a 5-μm Millipore filter and counted using a hemocytometer.

### Construction of protein serine/threonine phosphatasess knockout strains

All RHΔ*psp* strains were generated using the clustered regularly interspaced short palindromic repeats (CRISPR)/Cas9 technology as previously described (Wang et al., 2020a). All the primers used for the construction of the knock-out strains are listed in the Supplementary Table S1. Briefly, the specific CRISPR plasmids of the corresponding PSPs were engineered by substituting the UPRT targeting guide RNA in pSAG1:CAS9-U6:sgUPRT with SgRNA of each PSP, as previously described (Wang et al., 2020a). To construct 5HR-DHFR-3HR homologous templates of PSPs, the 5′ and 3′ homologous arms of genes flanking PSPs were amplified from *T. gondii* genomic DNA, and the DHFR gene was amplified from pUPRT-DHFR-D plasmid according to the Gibson assembly kit protocol (New England Biolabs, USA). The three fragments were then ligated into plasmid pUC19 to replace the coding region of the PSPs, as previously described (Wang et al., 2020a). The corresponding gene-specific CRISPR plasmid (∼40 μg) and the homologous template fragment of 5HR-DHFR-3HR (∼15 μg) were co-transfected into freshly egressed tachyzoites. The transfectants were selected by 3 μM pyrimethamine using limiting dilution in 96-well tissue culture plates (Thermo Scientific) as previously described (Wang et al., 2020a). Verification of the mutant strains was performed using PCRs.

### Epitope tagging and immunofluorescence detection of protein serine/threonine phosphatases

To determine the distribution of PSPs in type Ⅰ *T. gondii* RH strain, C-terminal endogenous tagging was performed as previously described (Zhang et al., 2021). The specific primers of each TgPSP were designed, and a fragment containing approximately 42 bp of the 3′ region of the *psp* gene (without the STOP codon), 6 × HA tag products were amplified by using p6 × HA-LIC-DHFR as a template. Similarly, TgPSP-specific CRISPR plasmid (∼40 μg) and purified fragment (∼15 μg) were co-transfected into the RH strain. Primers used for the construction of epitope-tagged strains are listed in the Supplementary Table S2. The positive clones were identified by PCRs.

To identify where PSPs reside within the host cell, we used indirect immunofluorescence assay (IFA). Briefly, epitope-tagged strains were used to infect HFF monolayers for 24 h. Cells infected with the WT strain were used as controls. The infected cells were fixed in 4% paraformaldehyde (PFA) in phosphate-buffered saline (PBS), permeabilized with 0.2% Triton X-100 in PBS, and blocked with 3% bovine serum albumin in PBS. The cells were then incubated with primary antibodies, including rabbit anti-IMC1 (1:1,000), mouse anti-HA (1:1,000), rabbit anti-HA (1:1,000), rabbit anti-GRA12 (1:500), or rabbit anti-ISP1 (1:500) (prepared in our laboratory) overnight and washed five times with ice-cold PBS. Subsequently, secondary antibodies: Alexa Fluor 488 goat anti-rabbit IgG (H + L) (1:1,000) and Alexa Fluor 594 goat anti-mouse IgG (H + L) (1:1,000) (Invitrogen, USA) were incubated with the samples for 1 h at 37°C, followed by washing five times with ice-cold PBS. Nuclei were stained with DAPI (4′,6-diamidino-2-phenylindole). Images were captured using a Leica confocal microscope (TCS SP52, Leica, Germany) (Wang et al., 2020a).

### Effect of protein serine/threonine phosphatases genes deletion on plaque formation of *T. gondii* RH strain

The size of plaques (cell monolayer areas devoid of cells) produced in the cell monolayers post infection is commonly used as an indicator of growth, where parasite strains that produce few and small plaques are considered less energetic. Here, we compared the growth rate of RHΔ*psp* and wild-type (WT) strains in HFFs using plaque assays. Briefly, ∼200 tachyzoites of each of RHΔ*psp* and WT strains, and ∼500 and 5,000 tachyzoites of PruΔ*pp7*, PruΔ*slp*, or WT strains were, respectively, inoculated into confluent HFF monolayers grown in 12-well tissue culture plastic plates (Thermo Scientific) and incubated at 37°C in a humidified environment with 5% CO_2_ for 7 days. The culture medium was then discarded and infected cells were fixed in 4% PFA for 30 min. To visualize plaques, fixed cells were stained with 2% crystal violet in PBS for 30 min at ambient temperature. The size and number of each plaque were determined by using a scanner as previously described (Wang et al., 2020a). The experiments were performed three independent times, each with three technical replicates.

### Effect of protein serine/threonine phosphatases genes deletion on intracellular replication of *T. gondii* RH strain

We examined whether *psp* genes play any role in the intracellular replication rate of *T. gondii*. Briefly, freshly egressed 10^5^ tachyzoites of RHΔ*psp* and WT strains were added to a confluent HFF monolayer growing on six-well plates (Thermo Scientific). Tachyzoites were allowed to infect HFFs for 1 h and then infected monolayers were washed several times with ice-cold PBS to remove unbound tachyzoites. The plates were incubated at 37°C and 5% CO_2_ for further 23 h. Subsequently, the samples were fixed with 4% PFA and stained with mouse anti-SAG1, followed by Alexa Fluor 488 goat-anti mouse IgG. At least 200 PVs were counted in each well to determine the number of intracellular tachyzoites produced by each strain. The data were obtained from three independent experiments.

### Effect of protein serine/threonine phosphatase gene deletion on egress of *T. gondii* RH strain

Approximately 10^5^ of RHΔ*psp* or WT tachyzoites per well were added into 12-well culture plastic plates containing monolayers of HFFs. The plates were maintained at 37°C and 5% CO_2_ for 1 h to allow tachyzoites to invade host cells. Then unbound parasites were removed with PBS, and fresh DMEM medium was added. After 30–36 h, the cells were treated with 3 μM calcium ionophore A23187 in DMEM (preheated at 37°C). Once the parasites started to exit the host cell, the coverslips were immediately fixed and the percentage of PVs was counted (Li et al., 2020). The experiments were performed three times.

### Effect of *pp7* deletion on parasite invasion and parasitophorous vacuole formation

About 2 × 10^6^ freshly egressed tachyzoites of RHΔ*pp7* and WT strains were added into HFFs grown on coverslips for 30 min and then were washed with sterile PBS three times to remove unattached tachyzoites followed by fixation with 4% PFA. The primary antibody rabbit anti-IMC1 (1:1,000 dilution for 1 h) and secondary antibody Alexa Fluor 488 goat anti-rabbit IgG (H + L) (1:1,000 dilution for 1 h) were added. The cells were washed three times with PBS to remove unbound antibodies, and permeabilized with 0.1% Triton X-100 in PBS. A second round of immunolabeling was carried out using a primary antibody (rabbit anti-IMC1, 1:1,000 dilution for 1 h) and a secondary antibody (Alexa Fluor 594 goat anti-rabbit IgG (H + L), 1:1,000 dilution for 1 h). The parasite invasion efficiency was determined by calculating the ratio of green-labeled/total tachyzoites detected in 20 microscopic fields per sample for each strain, as described previously (Leung et al., 2019).

The PV formation assay was performed by adding ∼5 × 10^5^ freshly egressed tachyzoites of RHΔ*pp7* or WT strain into confluent HFF monolayers grown on 12 wells for 30 min. Unattached tachyzoites were discarded by washing three times with PBS. Cells were incubated for 24 h before fixation with 4% PFA. Rabbit anti-IMC1 was used as a primary antibody, followed by secondary antibody Alexa Fluor 594 goat anti-rabbit IgG (H + L). For each sample, the number of PVs was randomly determined in at least 20 microscopic fields.

### Role of protein serine/threonine phosphatases in acute and chronic infection

To examine the role of *psps* in acute infection, freshly egressed tachyzoites were used to infect mice (6 mice/strain) by injecting 100 tachyzoites of RHΔ*psp* strains or WT strain in 200 μl PBS intraperitoneally (i.p.). Control mice were inoculated i.p. with 200 μl PBS only. We performed plaque assays in parallel to ensure that an accurate number of viable tachyzoites of each strain was used to infect the mice. To investigate the role of two *psps* (*pp7* and *slp*) in chronic infection, the mice were i.p. inoculated by low (500 tachyzoites) or high (5,000 tachyzoites) dose of PruΔ*pp7* and PruΔ*slp* (10 mice/strain). The infected mice were monitored at least twice daily for the clinical signs of toxoplasmosis, and the mice that have reached their humane endpoint were euthanized immediately. Thirty days post infection of mice by Pru, PruΔ*pp7,* or PruΔ*slp*, mouse brains were harvested, homogenized in 1 ml of PBS, and the parasite cyst’s burden in the brain homogenates was determined as described previously (Wang et al., 2018).

### Bioinformatics analysis of protein serine/threonine phosphatases

Genomic data (the number of exons, phenotype, and signal peptide) and transcriptomic data [cell cycle expression profiles (RH), tachyzoite transcriptome during invasion (RH), and oocyst, tachyzoite, and bradyzoite developmental expression profiles] of 17 PSPs were obtained from https://toxoDB.org. *PSP* gene expression analysis was performed using Robust Multiarray Average (RMA) algorithm of the Partek Genomics Suite package (Partek, Inc., St Louis, MO, United States).

### Statistical analysis

Statistical analysis was performed using GraphPad Prism version 8.4.0 for MacOS (GraphPad Software, La Jolla, CA, USA). Two-tailed unpaired Student’s *t*-test and one-way analysis of variance (ANOVA) were used to analyze the difference between infected and control groups. Error bars in the figures represent the standard deviation. *p* values <0.05 were considered statistically significant.

## Results

### Localization of 17 protein serine/threonine phosphatases proteins in type I RH strain

**FIGURE 1:**
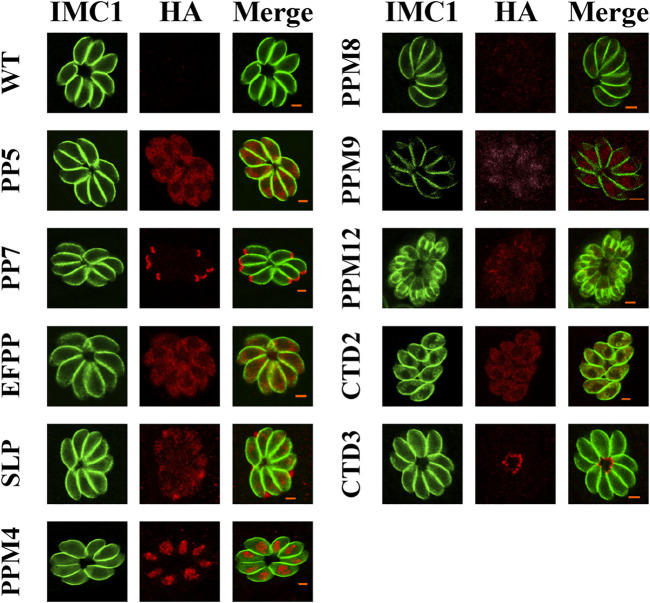
Subcellular localization of the protein serine/threonine phosphatases (PSPs) in *Toxoplasma gondii* RH strain. Human foreskin fibroblast (HFF) cells were infected with tachyzoites expressing HA tagged PSPs (PP5, PP7, EFPP, SLP, PPM4, PPM8, PPM9, PPM12, CTD2, and CTD3) for 24 h, and stained with antibodies against the HA epitope (red) and IMC1, a component of the inner membrane complex (green). The results showed that PP5, EFPP, PPM8, and CTD2 were located in the parasite cytoplasm. PP7, SLP, PPM4/PPM9, PPM12, and CTD3 were located in the parasite apical pole, dense granules, nucleus, inner membrane complex (IMC), and basal complex, respectively. Wild type (WT) tachyzoites did not show any staining. Scale bars = 2 μm.

**FIGURE 2:**
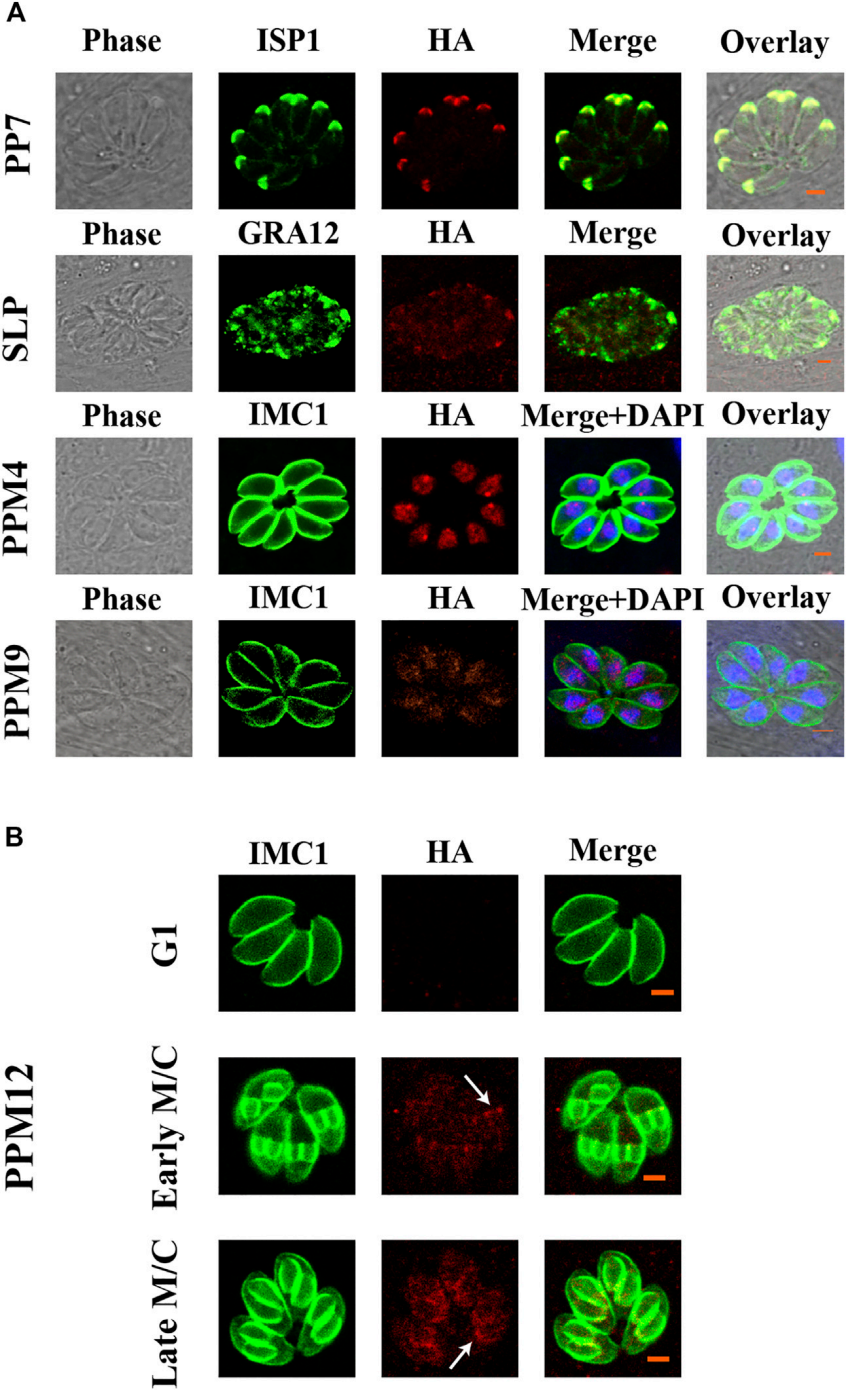
Verification of the localization of PP7, SLP, PPM4, PPM9, and PPM12. **(A)** PP7 was located in the apical pole and associated with the apical marker (ISP1). SLP appeared as several small puncta in the dense granules of tachyzoites. PPM4 and PPM9 were located in the nucleus as indicated by 4′,6-diamidino-2-phenylindole (DAPI) staining. **(B)** The expression of PPM12 in cell cycle G1, early M/C, and late M/C. PPM12 was expressed in M/C and localized in the plasma membrane, mainly in the basal complex. G1, G1 phase of cell cycle; M/C, completion of mitosis and budding. Arrows indicate basal complexes. Scale bars = 2 μm.

To investigate the subcellular localization of 17 PSPs, a 6 × HA tag was introduced into the C-terminus of these genes. All 17 *psp* genes were successfully tagged with 6 × HA as confirmed by PCR and DNA sequencing. The expression of seven PSPs, including PPM3F, PPM5A, PPM5B, PPM6, PPM14, PPM18, and CTD1 were low or undetectable in *T. gondii* RH tachyzoites. IFA results showed that PP5, EFPP, PPM8, and CTD2 were detected in the tachyzoite cytoplasm (Figure 1). However, the fluorescence of PPM8 was extremely dim and the accurate subcellular localization was not determined. SLP was localized in the cytoplasm and also appeared in dense granules (Figure 1). To further investigate the localization of this protein, we co-localized SLP with dense granules protein 12 (GRA12), and found that SLP was partly co-localized with GRA12 (Figure 2). PPM4 and PPM9 were detected in the nucleus of tachyzoites as revealed by DAPI staining (Figure 2). CTD3 accumulated in the tachyzoite basal complex in a punctate manner, which was similar to the partial localization of *T. gondii* Centrin2 (TgCEN2) (Figure 1) (Leung et al., 2019). As shown in Figure 1, the localization of PP7 coincides with the boundary between the apical cap of the parasite and the inner membrane complex (IMC). However, the localization of PP7 was predicted to be in the cytosol through hyperLOPIT in ToxoDB (https://toxodb.org/toxo/app) (Barylyuk et al., 2020). Further co-localization of PP7 with the IMC sub-compartment proteins (ISPs) (ISP1) showed that it is associated with ISP1 (Beck et al., 2010), confirming that PP7 is indeed located in the apical pole of tachyzoites (Figure 2). Interestingly, PPM12 was highly expressed when it was in endogenous division, located in the IMC, and mainly in the basal complex, which is consistent with its hyperLOPIT prediction and cell cycle expression (Figures 1 and 2; Supplementary Figure S1) (Behnke et al., 2010; Barylyuk et al., 2020).

### Disruption of protein serine/threonine phosphatases in type I and type II *T. gondii* strain

**FIGURE 3:**
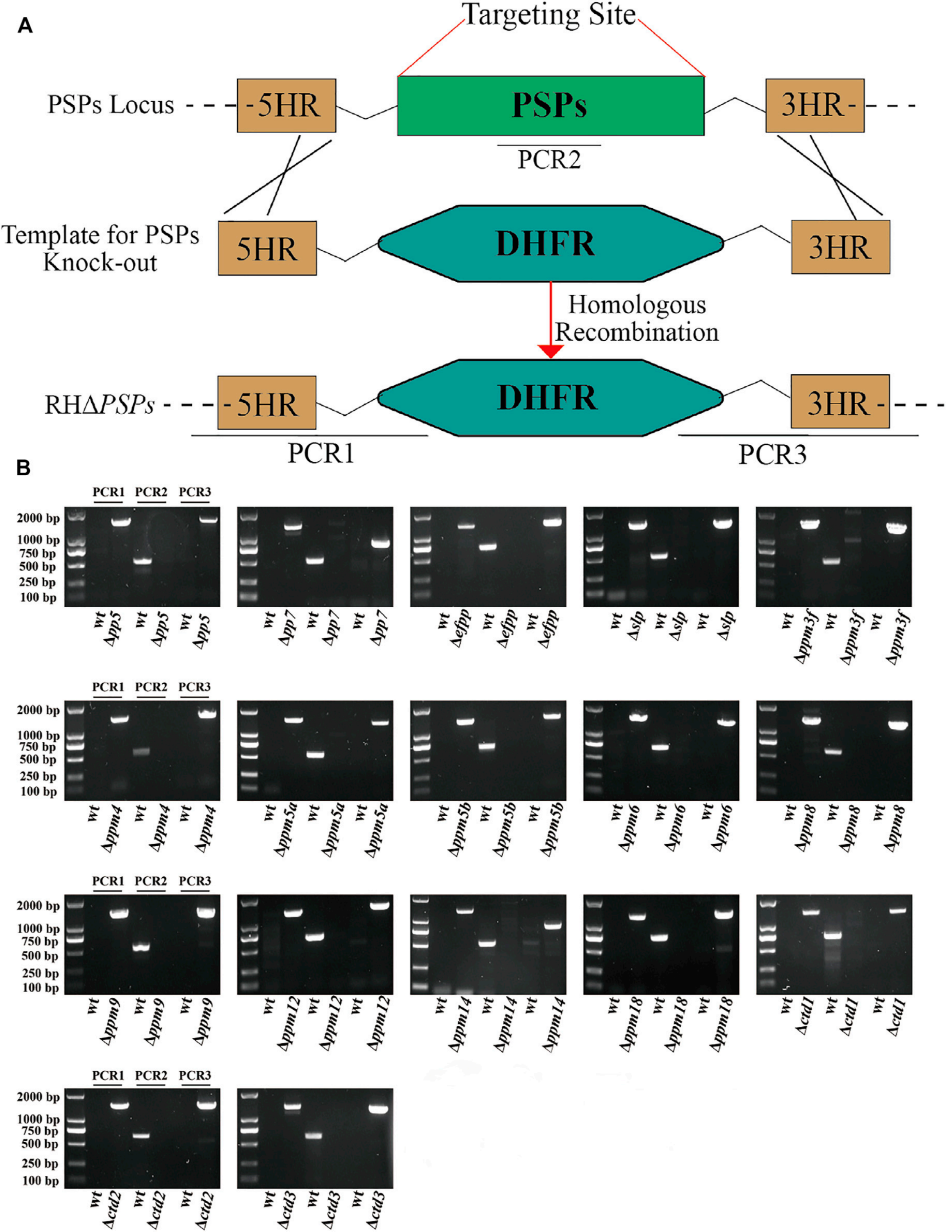
Construction of 17 protein serine/threonine phosphatases (PSPs) knock-out mutant of *T. gondii* RH strain. **(A)** Schematic illustration showing the replacement of the coding sequence of *psp* genes by CRISPR-Cas9 method. **(B)** PCR1 and PCR3 were used to detect 5′ and 3′ integration of homologous fragment, respectively, while PCR2 was used to detect whether the *PSP* genes were successfully replaced.

CRISPR-Cas9 system-mediated homologous recombination technology was used to knock-out the 17 *psp* genes (Figure 3A). The PSPs targeting the CRISPR-Cas9 plasmids and the templates 5HR-DHFR-3HR were co-transfected into RH tachyzoites so that the coding region of PSPs was homologously replaced by 5HR-DHFR-3HR, and single clones were obtained by using drug selection and limiting dilution. The expected small fragments (∼600–700 bp) were amplified using diagnostic PCR in the WT strain, while in the KO strains, these fragments were not amplified (Figure 3B). The replacement of homologous fragments was also examined by PCR, which amplified an ∼1,000–1,500 bp fragment, which was not detected in the WT strain (Figure 3B). The results showed that 17 *psp* genes were completely knocked-out using CRISPR-Cas9, and that RHΔ*psps*, PruΔ*pp7*, and PruΔ*slp* were successfully constructed.

### Effect of protein serine/threonine phosphatase gene deletion on plaque formation

**FIGURE 4:**
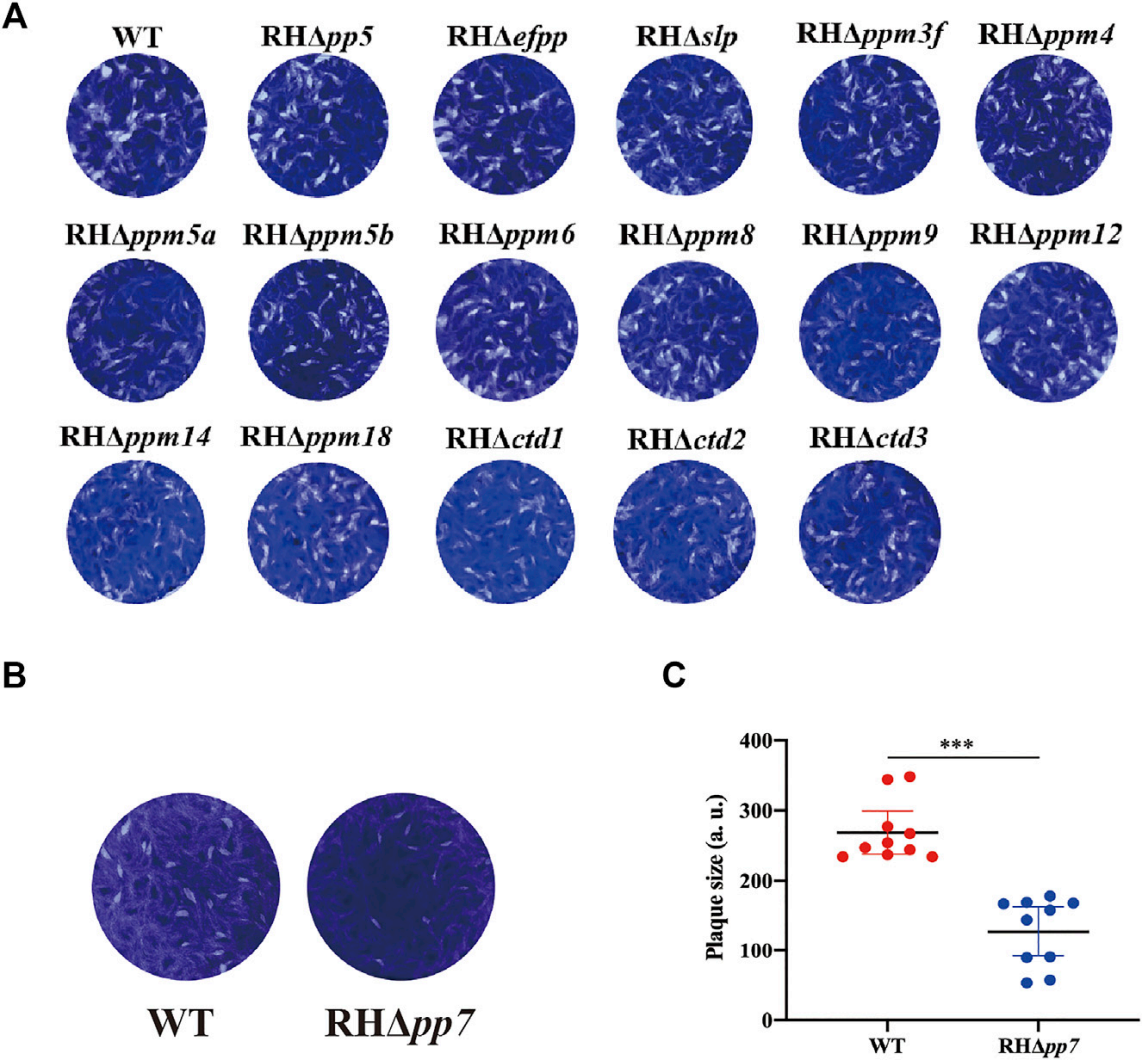
The lytic cycle of RHΔ*psp* and wild type (WT) strains. **(A)** Representative photographs of plaques observed in HFFs infected by 16 RHΔ*psp* and the WT strains. No significant differences in the number or size of plaques produced by any of the RHΔ*psp* strains compared with WT strain. **(B)** Representative photographs of plaques observed in HFFs infected RHΔ*pp7* tachyzoites compared with those produced by WT strain and their corresponding plaque sizes (**C**). Results showed a significant reduction in the number and size of plaques produced by the RHΔ*pp7* compared with WT strain. Each symbol represents a plaque. ***p < 0.001. All images are representative of results from three independent experiments.

Tachyzoites of RHΔ*psp* and WT strains were allowed to infect confluent HFF monolayers in 12-well culture plates. Following incubation for 7 days, plaques produced by the growth and proliferation of the parasite were visualized by staining with crystal violet. As shown in Figure 4A, there was no significant difference in the size and number of plaques between the 16 RHΔ*psp* strains and WT strain, while the size and number of plaques produced by RHΔ*pp7* were significantly reduced (Figures 4B, C).

### Effect of protein serine/threonine phosphatases genes deletion on parasite replication and egress

**FIGURE 5:**
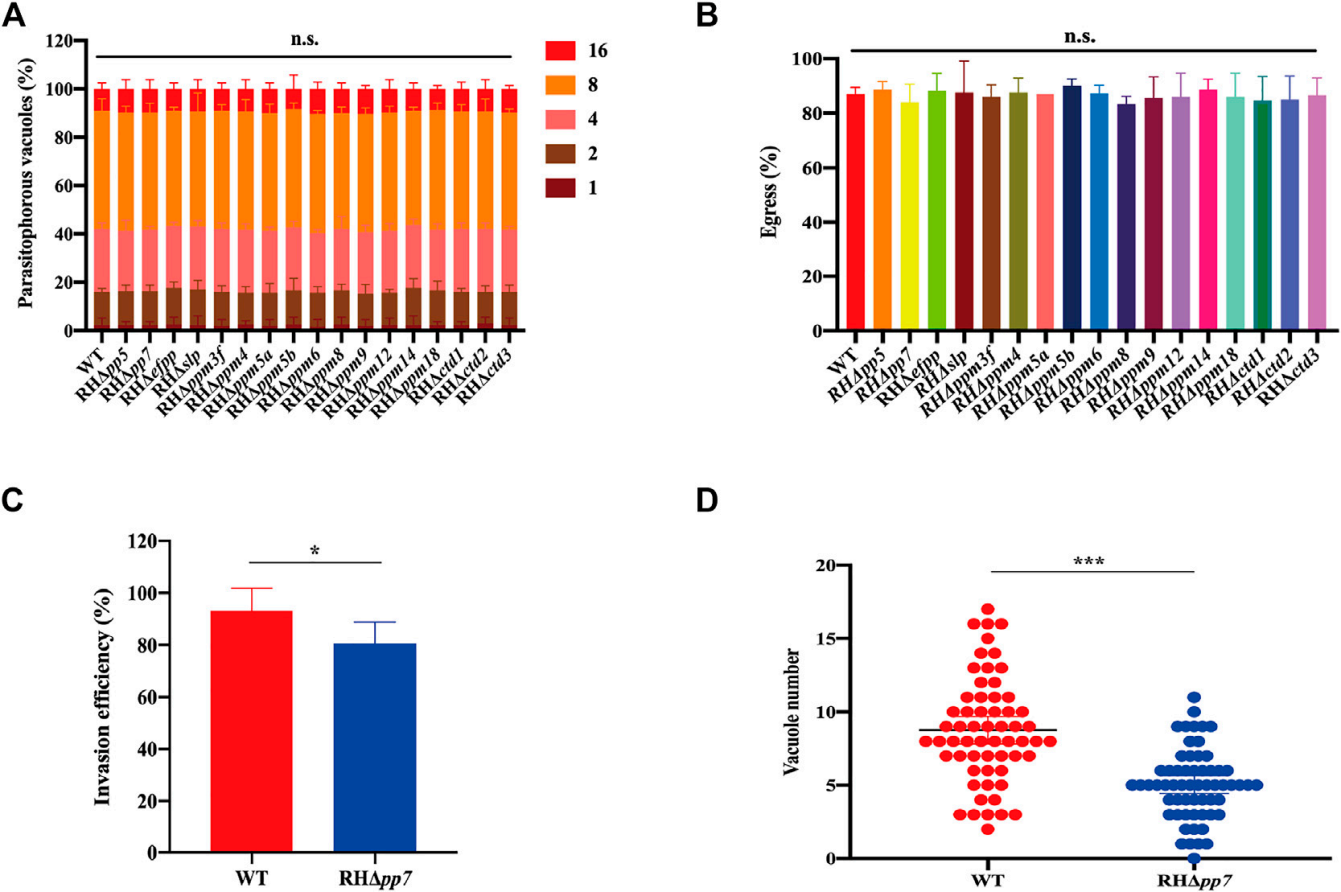
Intravacuolar replication, egress, invasion and parasitophorous vacuole formation in of RHΔ*psp* and WT strains. **(A)** Parasite replication was evaluated by counting the number of parasitophorous vacuole (PV) containing 1, 2, 4, 8, or 16 tachyzoites. At least 200 PVs were analyzed for each strain. RHΔ*psp* and WT strains have similar intracellular parasite replication kinetics. **(B)** No differences were detected in tachyzoites egress of the PVs between WT and RHΔ*psp* strains after adding 3 µM calcium carrier A23187 to the culture medium. **(C)** RHΔ*pp7* had significantly reduced invasion efficiency compared with the WT strain. **(D)** RHΔ*pp7* produced significantly less PV compared with WT strain. Each symbol represents a PV. n.s., not significant; *p < 0.05; ***p < 0.001, compared with the WT control.

To evaluate the role of PSPs in intracellular replication of tachyzoites, HFFs were infected with RHΔ*psp* and WT strains, and after 24 h, the numbers of tachyzoites inside the PVs were counted. The results showed no significant differences in the replication rate of tachyzoites within the PV between any of the 17 RHΔ*psp* strains and the WT strain (Figure 5A). We further investigated whether PSPs play a role in the parasite egress. HFFs were infected with equal number of tachyzoites of the RHΔ*psp* strains and WT strain. When the number of tachyzoites inside the PVs exceeded 32, the infected cells were treated with 3 µM calcium ionophore A23187 and fixed as soon as the egress process started. The results demonstrated that there were no significant differences in the percentages of parasite egress between the 17 RHΔ*psp* strains and WT strain (Figure 5B). These results show that infection by any of the 17 RHΔ*psp* strains did not cause any significant alteration in the ability of the parasite to proliferate or exit the host cells.

### Early stages of *T. gondii* RH infection are affected by deletion of *pp7*

Given that RHΔ*pp7* generates smaller and fewer plaques compared with the WT strain, we hypothesized that deletion of *pp7* reduces parasite invasion. To test this hypothesis, we first determined whether early steps of the parasite infection cycle (e.g., invasion) are affected by *pp7* deletion. HFF cell monolayers were infected by equal number of tachyzoites of RHΔ*pp7* and WT strains for 30 min. Then the ratio of intracellular tachyzoites to total number of tachyzoites was determined for each strain. We found that the ratio of intracellular to total number of tachyzoites was significantly lower in cells infected by RHΔ*pp7* compared with the WT strain (*p* < 0.05) (Figure 5C). We also determined number of PVs formed by each strain 24 h after infection. The number PVs formed by RHΔ*pp7* were significantly lower than that WT (*p* < 0.001) (Figure 5D).

### Virulence analysis of protein serine/threonine phosphatases in mice

**FIGURE 6:**
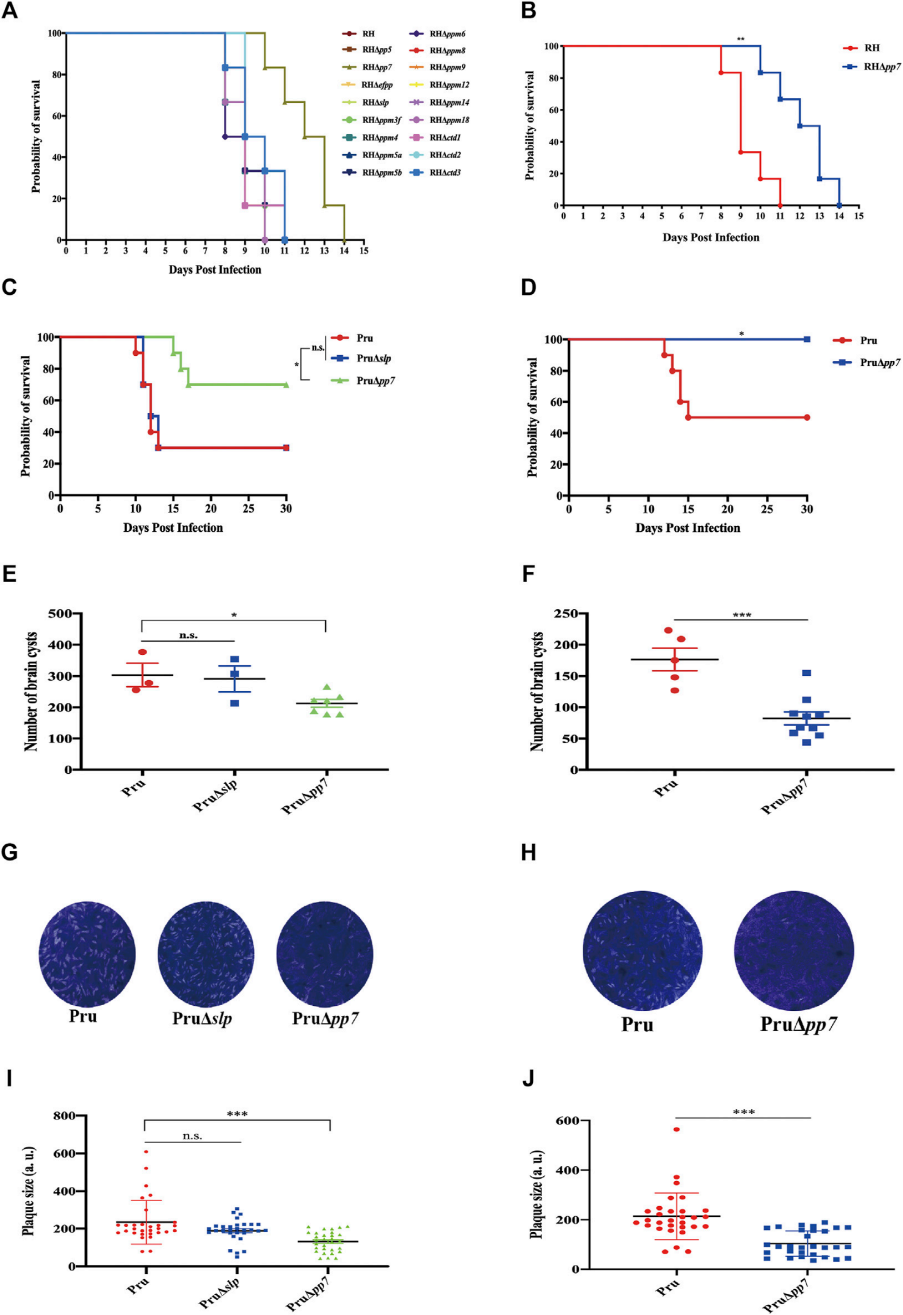
Survival curves of Kunming mice infected with *T. gondii* WT strain or PSP-deficient strains. Mice were checked twice daily by two independent observers for clinical signs and a Kaplan–Meier survival curve plotted of mice that have reached their humane endpoints. **(A)** Each group (*n* = 6 mice) of mice were injected intraperitoneally (i.p.) with 100 tachyzoites of the indicated strains. **(B)** Compared with the WT strain, the probability of survival of RHΔ*pp7*-infected mice was significantly increased, indicating the attenuation of RHΔ*pp7*. **(C,D)** Each group of mice (= 10) were injected i.p. with 5,000 (Pru, PruΔ*slp*, or PruΔ*pp7*), or 500 (Pru, or PruΔ*pp7*) tachyzoites of the indicated strains. **(E,F)** Parasite cyst burdens were quantified in the brains of mice that remained alive at 30 days of infection with 5,000 (Pru, PruΔ*slp*, or PruΔ*pp7*), and 500 (Pru or PruΔ*pp7*) tachyzoites. **(G,H, I,J)** Plaque assay using 5,000 (Pru, PruΔ*slp,* and PruΔ*pp7*) and 500 (Pru and PruΔ*pp7*) tachyzoites revealed no significant differences in the number or size of plaques produced by PruΔ*slp* vs. Pru strain. However, the PruΔ*pp7* strain has produced significantly less plaques compared with Pru and PruΔ*slp*. Infection with 500 tachyzoites showed that plaques formed by PruΔ*pp7* were significantly lower than those produced by Pru strain. n.s., not significant; *p < 0.05; **p < 0.01; ***p < 0.001, compared with the control Pru strain.

We assessed whether the deletion of 17 *psp* genes could alter the virulence of *T. gondii* RH strain. Approximately 100 tachyzoites of each RHΔ*psp* strain and WT strain were injected i.p. into Kunming mice, and the mice were monitored for the development of illness. Mice infected by each of the 16 RHΔ*psps* (PP5, EFPP, SLP, PPM3F, PPM4, PPM5A, PPM5B, PPM6, PPM8, PPM9, PPM12, PPM14, PPM18, CTD1, CTD2, CTD3) and WT strain reached their humane endpoint within 8–11 days, indicating that these genes do not contribute to parasite virulence in mice (Figure 6A). However, the mice infected with RHΔ*pp7* strain had better survival than the mice infected with the WT strain (*p* < 0.01) (Figure 6B).

We also investigated the role of *pp7* and *slp* in chronic infection. We evaluated the effects of i.p. inoculation by high dose (5,000 tachyzoites) of PruΔ*pp7* and PruΔ*slp* on mice. The results showed that deletion of *slp* in Pru strain did not reduce the parasite virulence. However, PruΔ*pp7*, similar to RHΔ*pp7*, significantly reduced the virulence of *T. gondii* in mice infected with 5,000 tachyzoites compared with WT and PruΔ*slp* (Figure 6C). Results of the parallel plaque assay showed that the size and number of plaques formed by PruΔ*pp7* were significantly lower than that produced by WT and PruΔ*slp* (*p* < 0.05) (Figures 6G, I).

Kunming mice in Figure 6C that remained alive at 30 days after infection were euthanized, and the number of cysts in the mouse brain was determined. As shown in Figure 6E, there was no significant difference between the brain cysts formed by WT and PruΔ*slp*; however, the brain cyst burden formed by PruΔ*pp7* was significantly lower than that detected in mice infected by WT or PruΔ*slp* (*p* < 0.05).

In view of the attenuated virulence of PruΔ*pp7*, we performed a low dose infection with 500 tachyzoites. As shown in Figures 6D, F, H, ,J no death was observed in mice infected with 500 PruΔ*pp7*, and the parasite cyst burden and number of plaques of PruΔ*pp7* were significantly lower than that of WT, suggesting that loss of *pp7* gene in Pru strain markedly attenuated the virulence of the tachyzoites.

### Expression analyses of protein serine/threonine phosphatases in *T. gondii*

To investigate the transcription levels of 17 *PSP* genes in different genotypes, cell cycles and life cycle stage of *T. gondii*, we analyzed microarray transcriptomic data in the ToxoDB (https://toxodb.org/toxo/app). Based on the transcriptional profiles of different genotypes (Type I, II, and III) of *T. gondii*, we found that *pp5*, *efpp*, *ppm3f*, *ppm5b*, *ppm8*, and *ppm9* were significantly different between the three *T. gondii* genotypes (Supplementary Figure S1A). Next, we analyzed the expression profiles of 17 PSPs per cell cycle phase and found that *ppm3f*, *ppm12*, *ppm18,* and *ctd3* followed a specific cell cycle pattern, while most of them did not have a particular expression pattern, and the expression levels of *slp*, *ppm5a*, *ppm6,* and *ctd2* were low (Supplementary Figure S1B) (Behnke et al., 2010). The expression levels of the 17 *psp* genes in each developmental stage of *T. gondii* are shown in the Supplementary Figure S1C (Fritz et al., 2012). Some *psp* genes, including *efpp*, *slp*, *ppm3f*, *ppm5a, ppm5b*, *ppm8,* and *ppm12* were differentially expressed, while *pp5*, *pp7*, *ppm4*, *ppm6*, *ppm9*, *ppm14*, *ctd1*, *ctd2*, and *ctd3* were constitutively expressed. A summary of bioinformatics characteristics of the *psp* genes, including the number of exons, CRISPR phenotype, and signal peptides are provided in Table 1. Most of the *psp* genes are encoded with multiple exons, with pp7 having the highest number of exons, and most of the *psp* genes did not have a transmembrane domain and signal peptide.

**TABLE 1:** Bioinformatic features of protein serine/threonine phosphatases (PSPs) of *Toxoplasma gondii*.

Name	Gene ID	Product description	Exons	Phenotype value	TMHMM^a^	Molecular weight (kD^a^)	Predicted signal peptide
PP5	TGGT1_312200	Serine/threonine protein phosphatase	8	0.71	No	61.55	No
PP7	TGGT1_251850	Serine/threonine protein phosphatase	17	0.05	No	117.32	No
EFPP	TGGT1_269460	Ser/Thr phosphatase family protein	13	-0.81	No	218.51	No
Shewanella-like phosphatases (SLP)	TGGT1_254770	Ser/Thr phosphatase family protein	1	0.81	No	46.43	No
PPM3F	TGGT1_278510	Protein phosphatase 2C domain-containing protein	10	1.01	Yes	47.19	Yes
PPM4	TGGT1_208500	PPM-type phosphatase domain-containing protein	10	0.84	No	78.28	No
PPM5A	TGGT1_272280	Putative PP2C	8	−0.55	No	81.38	No
PPM5B	TGGT1_318660	Putative PP2C	3	0.83	No	59.39	No
PPM6	TGGT1_293450	Protein phosphatase 2c containing protein	2	1.75	No	27.59	No
PPM8	TGGT1_218590	PPM-type phosphatase domain-containing protein	7	0.43	No	97.68	No
PPM9	TGGT1_220610	PPM-type phosphatase domain-containing protein	7	1.23	No	58.12	No
PPM12	TGGT1_207590	Protein phosphatase	3	0.37	No	57.8	No
PPM14	TGGT1_232010	PPM-type phosphatase domain-containing protein	3	−0.65	No	440.42	No
PPM18	TGGT1_270190	PPM-type phosphatase domain-containing protein	5	−0.11	No	153.63	No
CTDSPL1 (CTD1	TGGT1_310660	FCP1 homology domain-containing protein	10	0.77	No	70.8	No
CTDSPL2 (CTD2)	TGGT1_263380	FCP1 homology domain-containing protein	5	0.65	No	26.68	No
CTDSPL3 (CTD3)	TGGT1_202550	FCP1 homology domain-containing protein	4	−0.39	No	232.95	No

Note. a Prediction of transmembrane helices was performed using the TMHMM, program version 2.0.

## Discussion

The phosphatases and their regulatory proteins can play key roles in mediating the interaction between the apicomplexan protozoa with their host cells (Cieśla et al., 2011; Yang and Arrizabalaga, 2017). In the present study, we investigated the subcellular location of 17 *PSPs* in *T. gondii* RH strain. Seven of these (PPM3F, PPM5A, PPM5B, PPM6, PPM14, PPM18, and CTD1) were not expressed or expressed at low levels in the tachyzoites. Four PSPs (PP5, EFPP, PPM8, and CTD2) were mainly located in the cytoplasm, and two reside in the nucleus (PPM4 and PPM9), and PPM12 and CTD3 were located in the IMC and basal complex. SLP was located partly at dense granule and PP7 was localized at the apical pole. Unlike most PPPs, PP7 has catalytic and regulatory domains in a single peptide, which explains why PP7 can be used as monomer phosphatase, similar to PP5 (Yang and Arrizabalaga, 2017).

With the exception of RHΔ*pp7* strain, there were no significant differences in the number and size of plaques produced by infection with any of the RHΔ*psp* strains compared with the WT stain. The size of plaques is directly related to the extent of damage upon infection of a cell monolayer, and thus, is considered a proxy measure of parasite virulence, suggesting that deletion of *pp7* gene has resulted in attenuation of the virulence or fitness of *T. gondii*. Likewise, no significant differences were detected in the egress ability of all 17 RHΔ*psp* and WT strains. Additionally, the intracellular replication assays showed no significant differences between 17 RHΔ*psp* strains and WT strain (*p* > 0.05).

Little is known about the biological functions of some PSPs, which were involved in various stages of the parasite lytic cycle (Yang and Arrizabalaga, 2017). PPM3C is a protein phosphatase secreted into the PVs and Δ*ppm3c* tachyzoites, which exhibits growth defects *in vitro*, is avirulent during acute infection and forms fewer cysts in chronically infected mice (Mayoral et al., 2020). When PPM3C is secreted into the PVs, vacuolar phosphoprotein substrates, such as GRA16 and GRA28, become de-phosphorylated, leading to exporting of other effectors, and maintaining of the host cell functions to sustain the growth and proliferation of the parasite (Mayoral et al., 2020). Calcineurin is known as PP2B or protein phosphatase (PP3), a protein phosphatase belonging to the PPPs family, which plays an indispensable role in the signal transduction in eukaryotes (Paul et al., 2015). Knocking out of the catalytic subunit (TgCnA) of *Plasmodium falciparum* and *T. gondii* PP2B significantly reduced the ability of the parasite to attach to and invade host cells, without affecting the parasite egress or the function of microneme and rhoptry (Paul et al., 2015). Similar results were reported in PPM5C, where Δ*ppm5c* was found to only affect the attachment to host cells during parasite lytic cycle (Yang et al., 2019).

Limited information is available about the PSPs that contribute to the virulence of *T. gondii*, such as PPM3C (Mayoral et al., 2020). PPM20 is a rhoptry-localized protein phosphatase and although RHΔ*ppm20* exhibits slow growth *in vitro*, its virulence in mice is not altered (Gilbert et al., 2007). We examined whether any of the 17 PSPs contributes to the virulence of *T. gondii*. In 16 RHΔ*psps*, all mice died within 8–11 days. Only PP7 was unique in terms of subcellular localization and virulence, compared with other PSPs, as demonstrated by the significant reduction of RHΔ*pp7* pathogenicity in mice. This is not surprising, PP7 is regulated by calcium signaling (Kutuzov et al., 2001) and is involved in various cellular processes, such as cell survival and growth, and has a glycine (CAP-Gly) domain at the N-terminus before calmodulin-binding motif, which is a cytoskeletal-related protein (Andreeva and Kutuzov, 2009).

Given the significant role of PP7 in the parasite lytic cycle, we expanded our investigation to characterize the role of PP7 in the growth kinetics of *T. gondii* type II Pru strain *in vitro*. Results showed that PruΔ*pp7* strain exhibited substantial reduction in the size and number of plaques compared with the control strain. We also examined the effect of deletion of *pp7* on the virulence of Pru *in vivo*. Mice infected by PruΔ*pp7* showed a high survival rate and lower brain cyst burden compared with mice infected by the WT strain.

We examined the transcriptome data of 17 PSPs in ToxoDB (https://toxodb.org/toxo/app) and observed that the expression patterns vary according to the genotype, cell cycle, and life cycle form of *T. gondii*. For example, *slp* was mainly expressed in the sexual stage of parasite, and had low expression in the asexual stage. This finding concurs with a previous study which showed that deletion of *slp* in *P. falciparum* affects the development of ookinete and inhibits the formation of oocyst (Fernandez-Pol et al., 2013), suggesting that *slp* may have a role in the sexual stage of *T. gondii*. Although *slp* is more expressed in type II ME49 strain, its deletion in type II Pru strain did not have any significant impact on the parasite infectivity *in vitro* or pathogenicity *in vivo*.

## Conclusion

We characterized the subcellular location and the role of 17 PSPs in *T. gondii* RH strain and 2 PSPs in Pru strain, *in vitro* and *in vivo*. Epitope-tagging and immunofluorescence analysis showed that PP5, EFPP, PPM8, and CTD2 are located in the cytoplasm of tachyzoites, while PP7, SLP, CTD3, PPM12, and PPM4/PPM9 are located in the apical pole, dense granules, basal complex, IMC, and nucleus, respectively. The other PSPs (PPM3F, PPM5A, PPM5B, PPM6, PPM14, PPM18, and CTD1) were either not expressed or showed low expression in type I RH tachyzoites. The CRISPR/Cas9-mediated deletion of 16 *psp* genes did not significantly affect the *in vitro* growth ability of the RH strain. However, deletion of *pp7* caused a significant reduction in the growth and invasion of tachyzoites compared with those of the WT strain. Infection with RHΔ*pp7* significantly increased the survival of mice compared with infection by other RHΔ*psps* and WT strains. Infection by a low dose of PruΔ*pp7* showed a marked attenuation of virulence and significant reduction in brain cyst loads. Bioinformatics analysis of the 17 *psp* genes indicated that their expression patterns vary based on the parasite genotype, cell cycle phase, and developmental stage. These findings advance our understanding of the molecular basis of *T. gondii* virulence and the *in vivo* consequences of deletion of protein Ser/Thr phosphatase PP7.

## Data Availability

The original contributions presented in the study are included in the article/Supplementary Material, further inquiries can be directed to the corresponding authors.
