# Supplementary figures and images for: Functional Characterization of 17 Protein Serine/Threonine Phosphatases in Toxoplasma gondii Using CRISPR-Cas9 System (part 1 of 2)
Source: Front Cell Dev Biol. 2022 Jan 10;9:738794. doi: 10.3389/fcell.2021.738794 (PMC8785970; doi:10.3389/fcell.2021.738794)

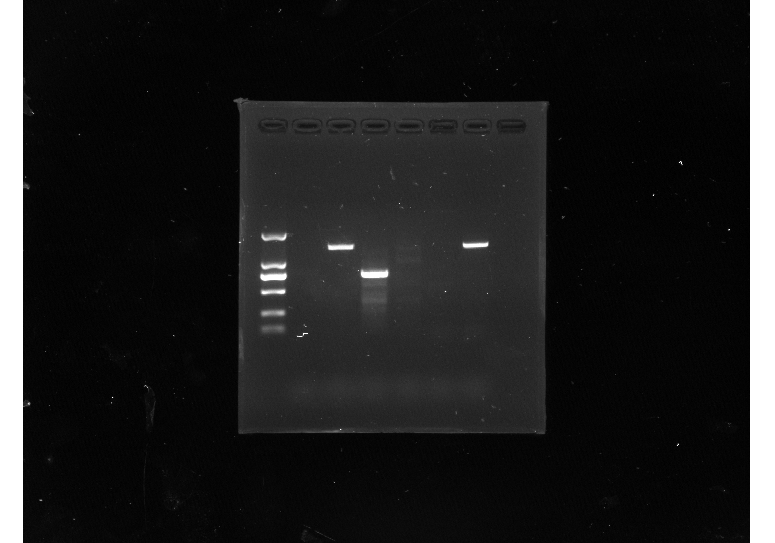

Supplement: Supplementary file 1 [file DataSheet3.ZIP › Others/Figure 3/ctd1.tif]

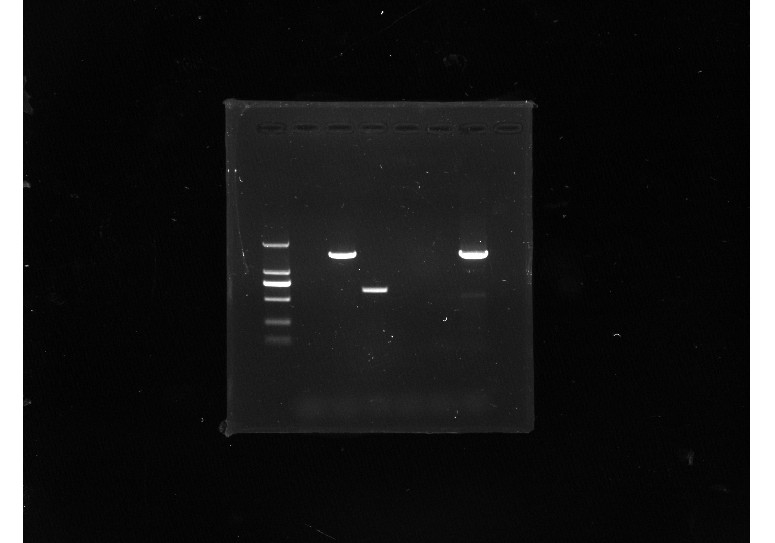

Supplement: Supplementary file 1 [file DataSheet3.ZIP › Others/Figure 3/ctd2.tif]

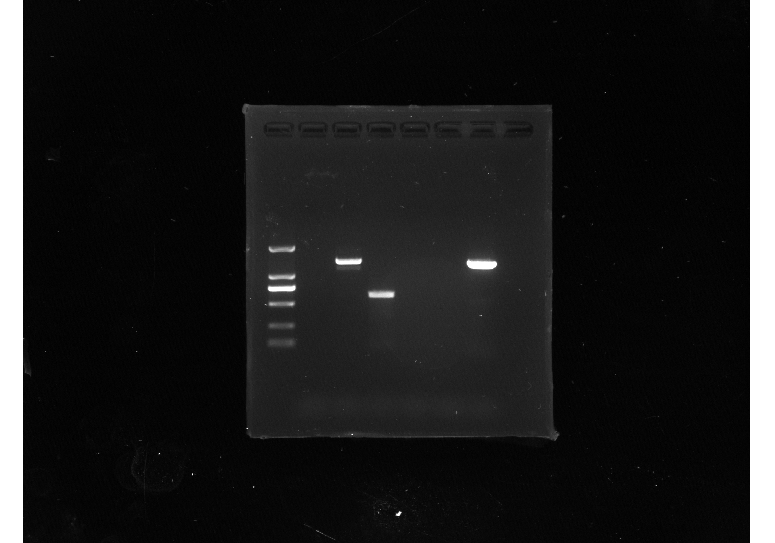

Supplement: Supplementary file 1 [file DataSheet3.ZIP › Others/Figure 3/ctd3.tif]

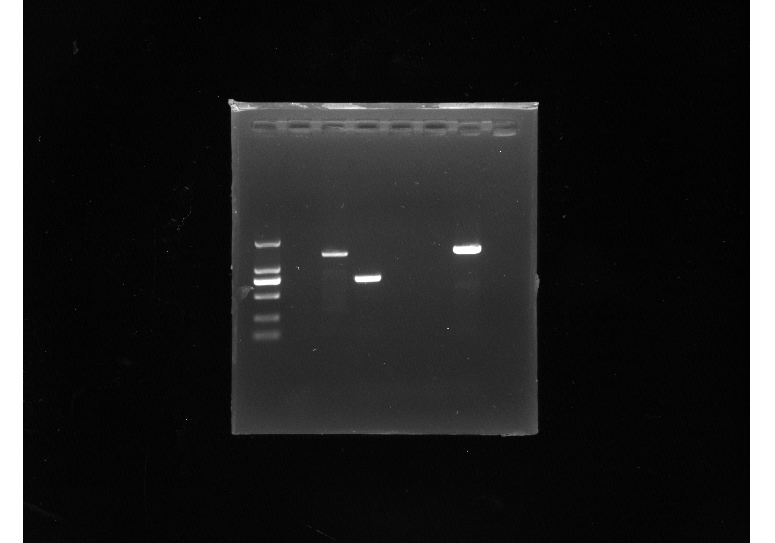

Supplement: Supplementary file 1 [file DataSheet3.ZIP › Others/Figure 3/efpp.tif]

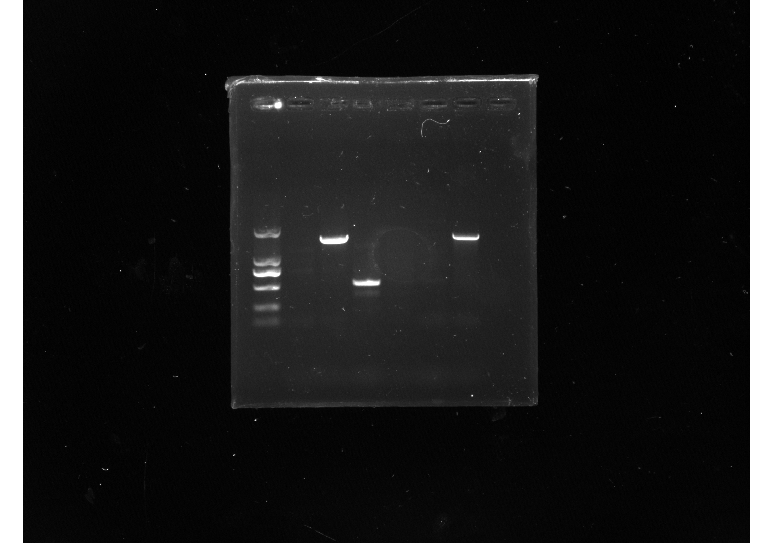

Supplement: Supplementary file 1 [file DataSheet3.ZIP › Others/Figure 3/pp5.tif]

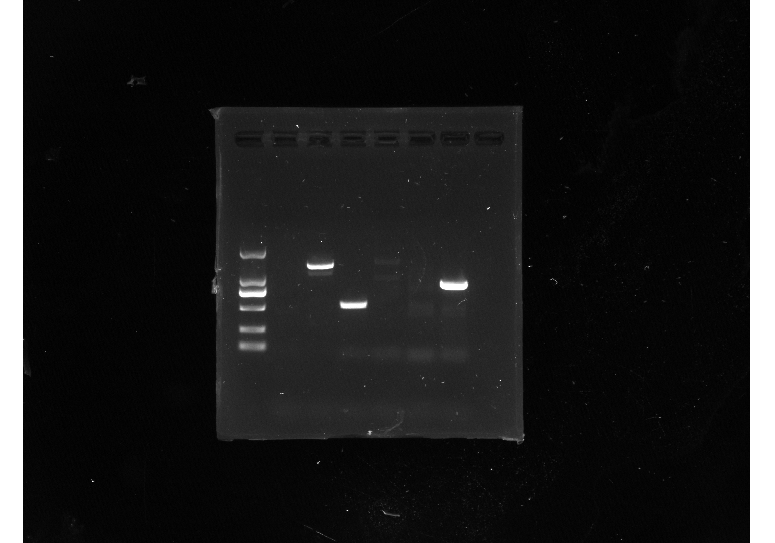

Supplement: Supplementary file 1 [file DataSheet3.ZIP › Others/Figure 3/pp7.tif]

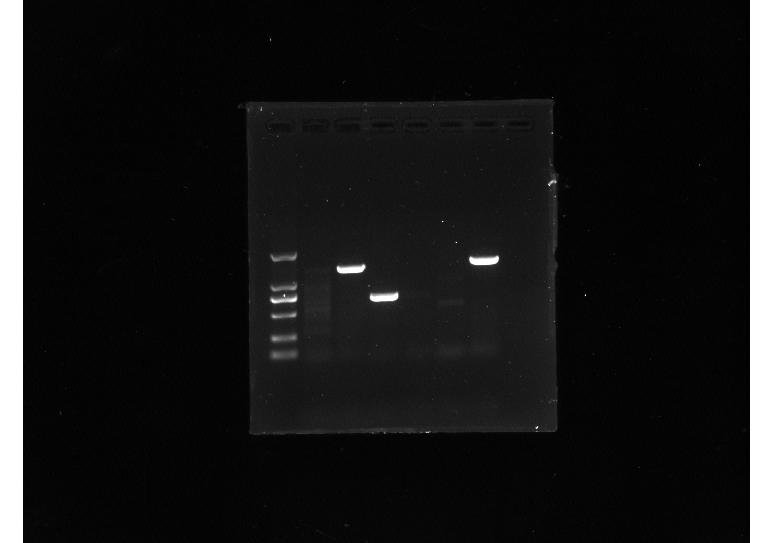

Supplement: Supplementary file 1 [file DataSheet3.ZIP › Others/Figure 3/ppm12.tif]

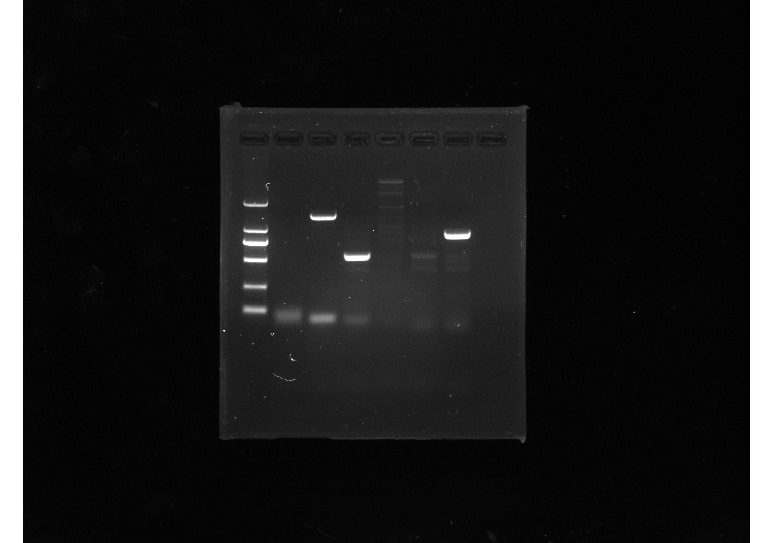

Supplement: Supplementary file 1 [file DataSheet3.ZIP › Others/Figure 3/ppm14.tif]

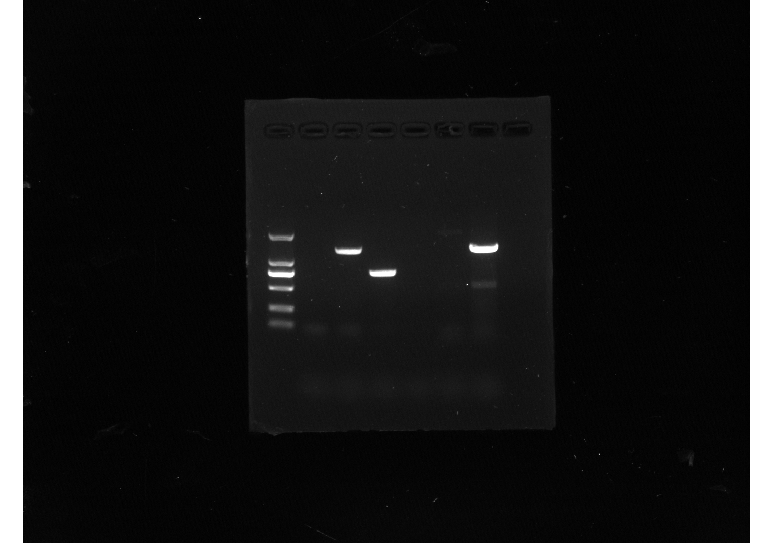

Supplement: Supplementary file 1 [file DataSheet3.ZIP › Others/Figure 3/ppm18.tif]

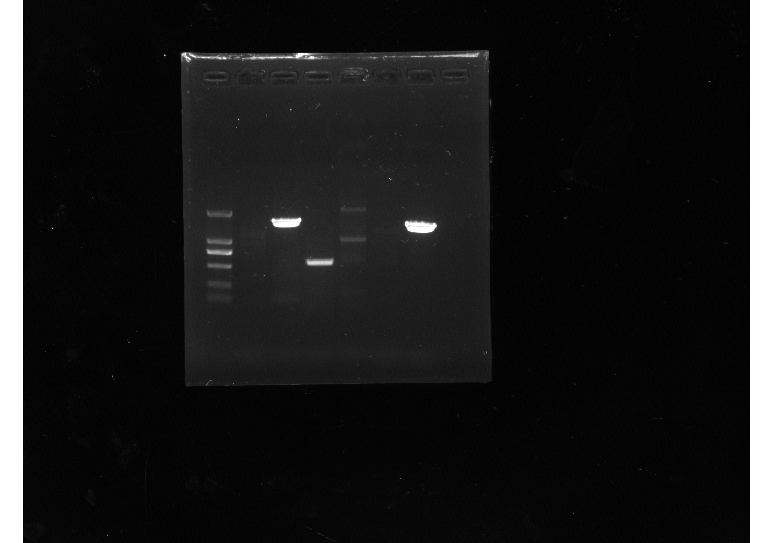

Supplement: Supplementary file 1 [file DataSheet3.ZIP › Others/Figure 3/ppm3f.tif]

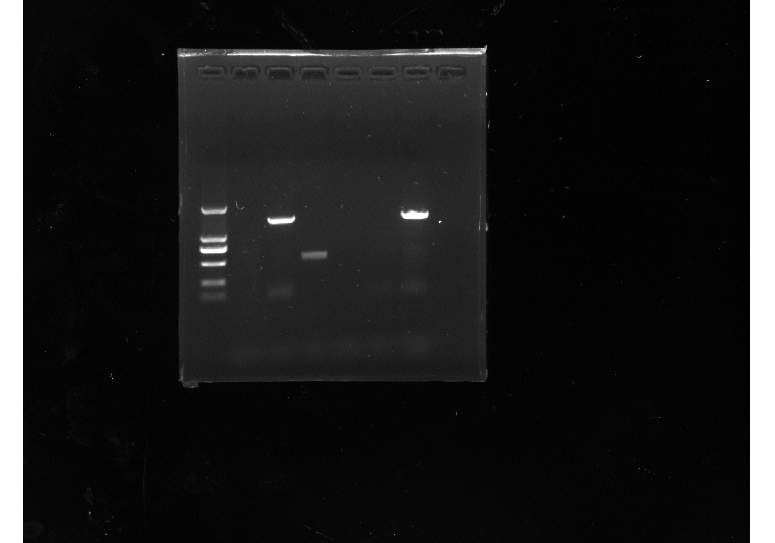

Supplement: Supplementary file 1 [file DataSheet3.ZIP › Others/Figure 3/ppm4.tif]

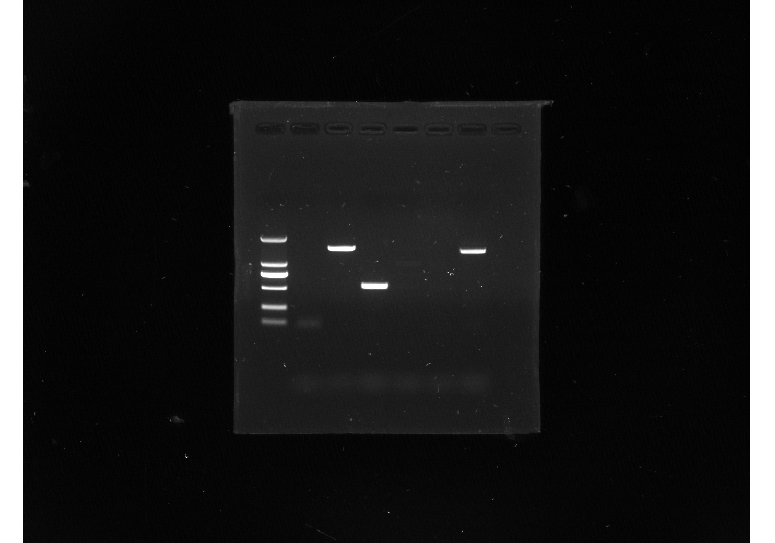

Supplement: Supplementary file 1 [file DataSheet3.ZIP › Others/Figure 3/ppm5a.tif]

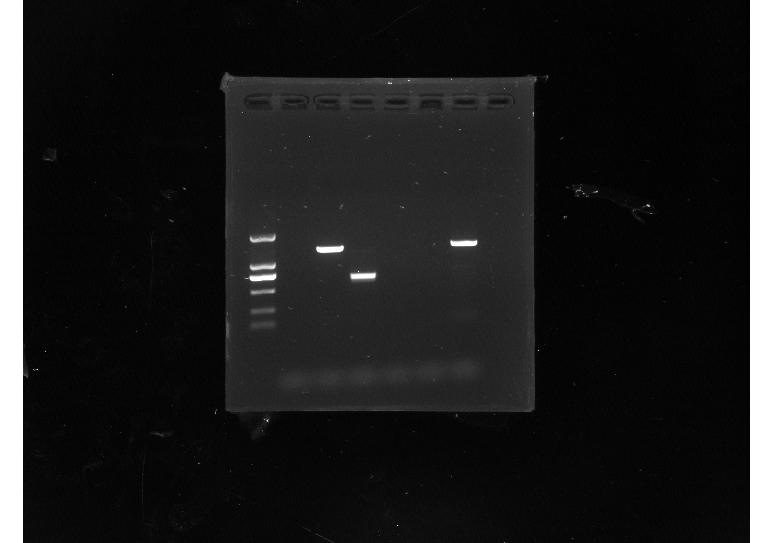

Supplement: Supplementary file 1 [file DataSheet3.ZIP › Others/Figure 3/ppm5b.tif]

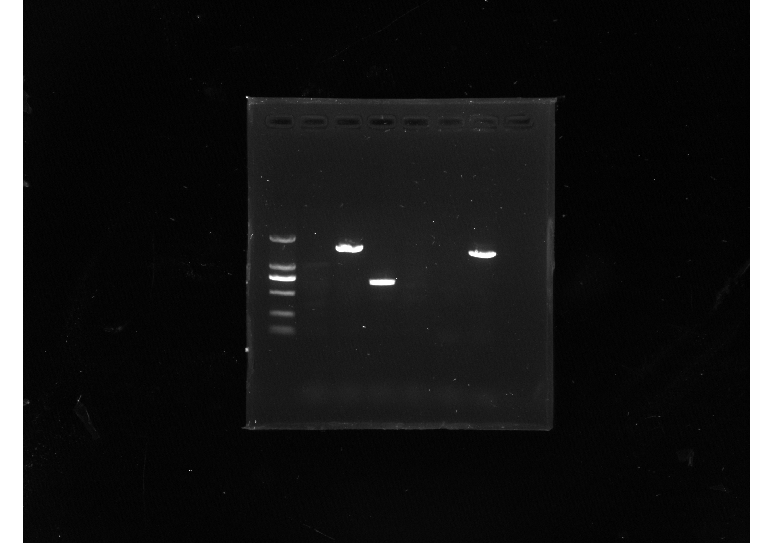

Supplement: Supplementary file 1 [file DataSheet3.ZIP › Others/Figure 3/ppm6.tif]

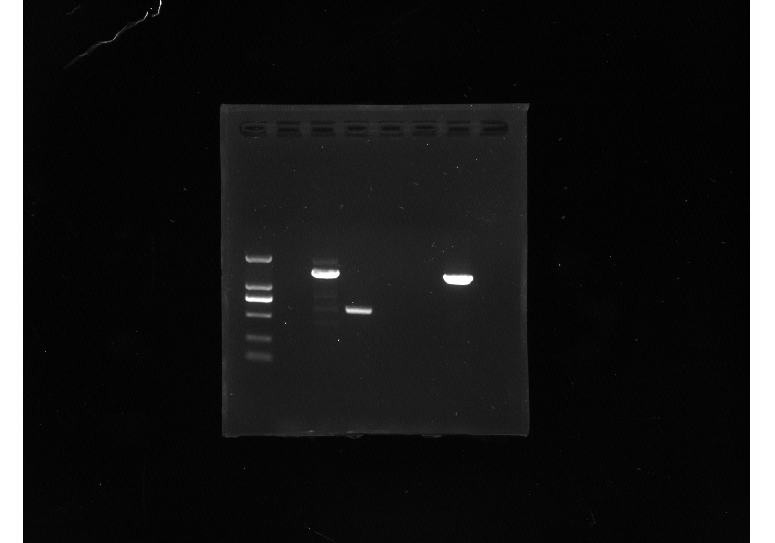

Supplement: Supplementary file 1 [file DataSheet3.ZIP › Others/Figure 3/ppm8.tif]

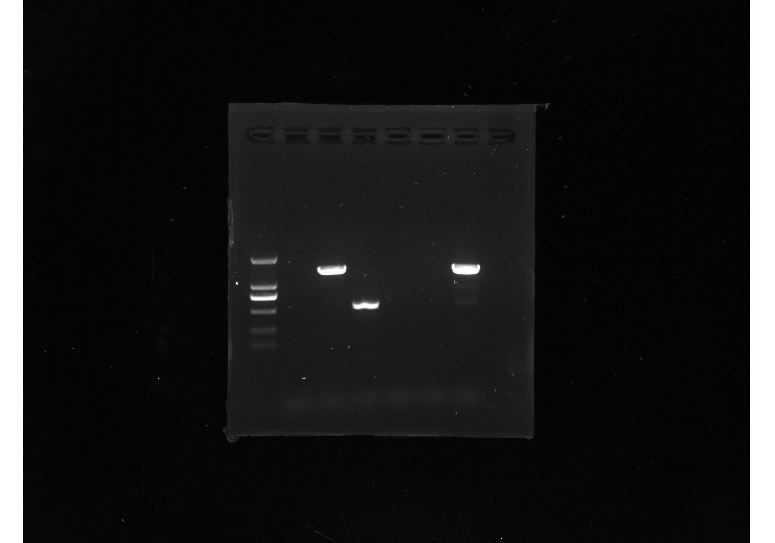

Supplement: Supplementary file 1 [file DataSheet3.ZIP › Others/Figure 3/ppm9.tif]

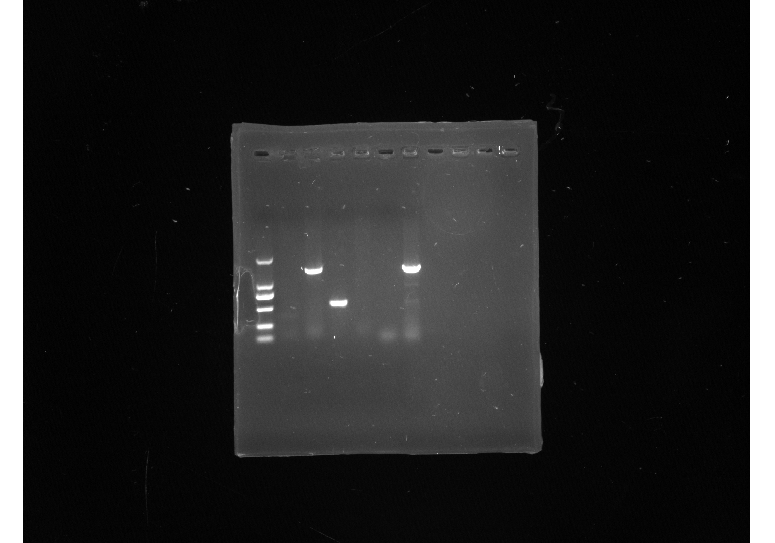

Supplement: Supplementary file 1 [file DataSheet3.ZIP › Others/Figure 3/Pruslp-ko.tif]

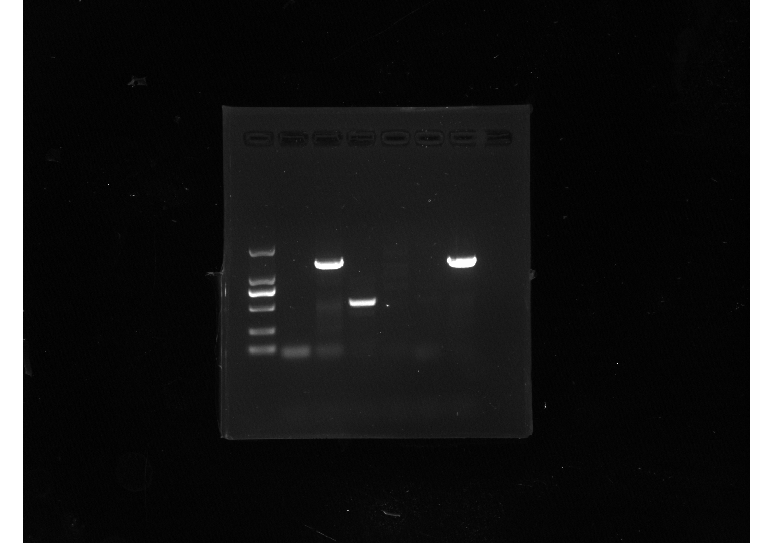

Supplement: Supplementary file 1 [file DataSheet3.ZIP › Others/Figure 3/slp.tif]

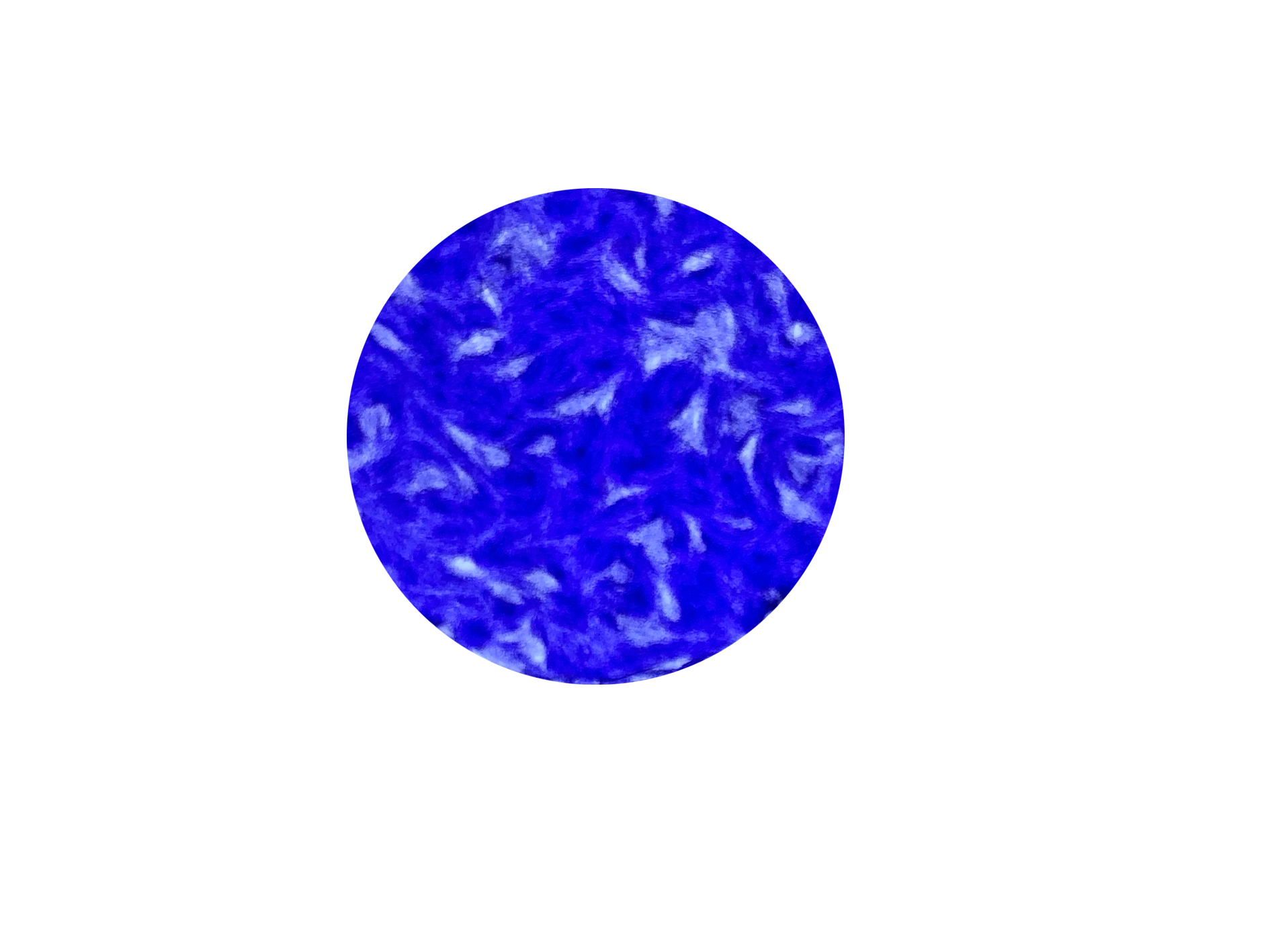

Supplement: Supplementary file 1 [file DataSheet3.ZIP › Others/plaque assay/ctd1.png]

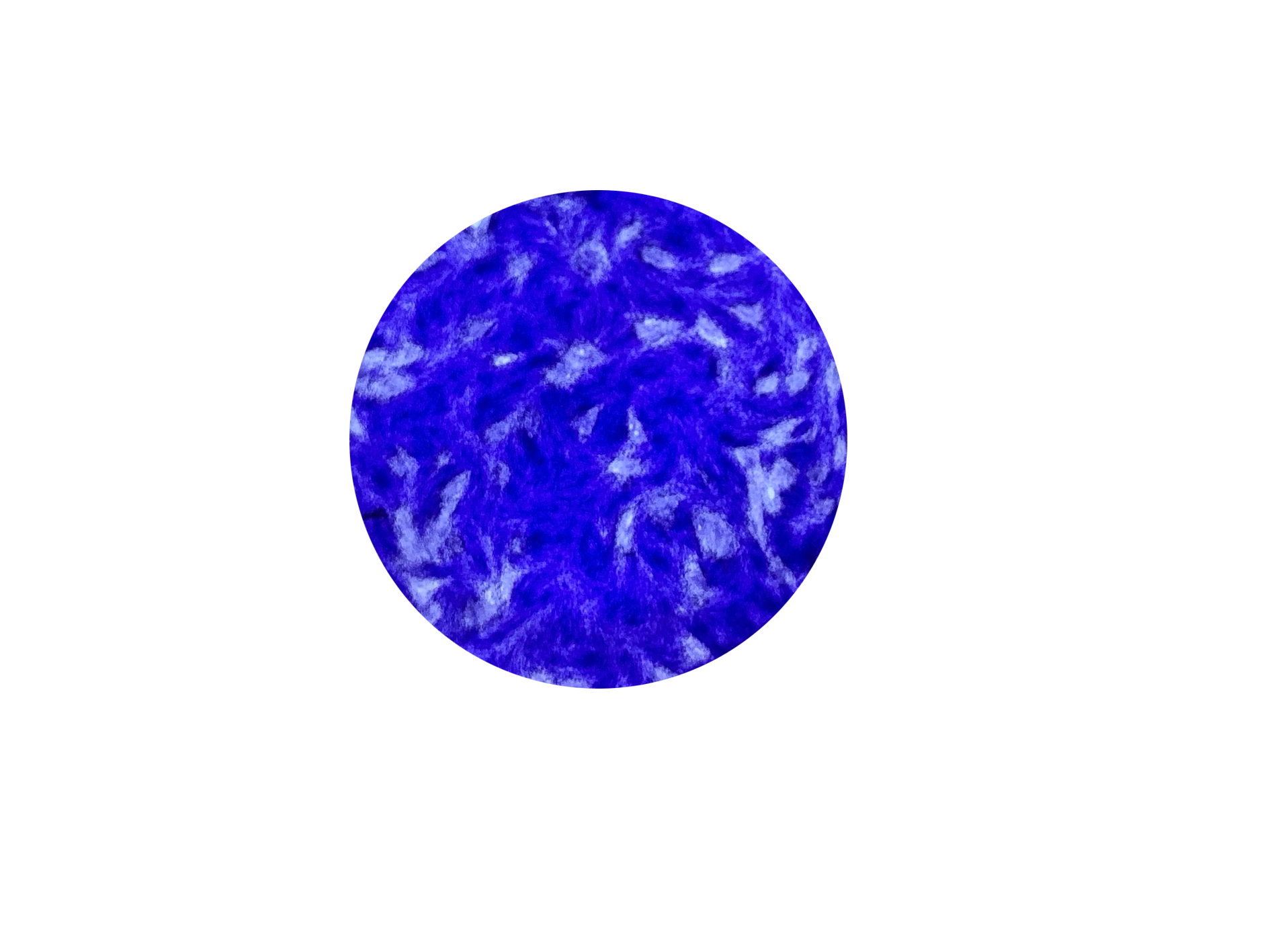

Supplement: Supplementary file 1 [file DataSheet3.ZIP › Others/plaque assay/ctd2.png]

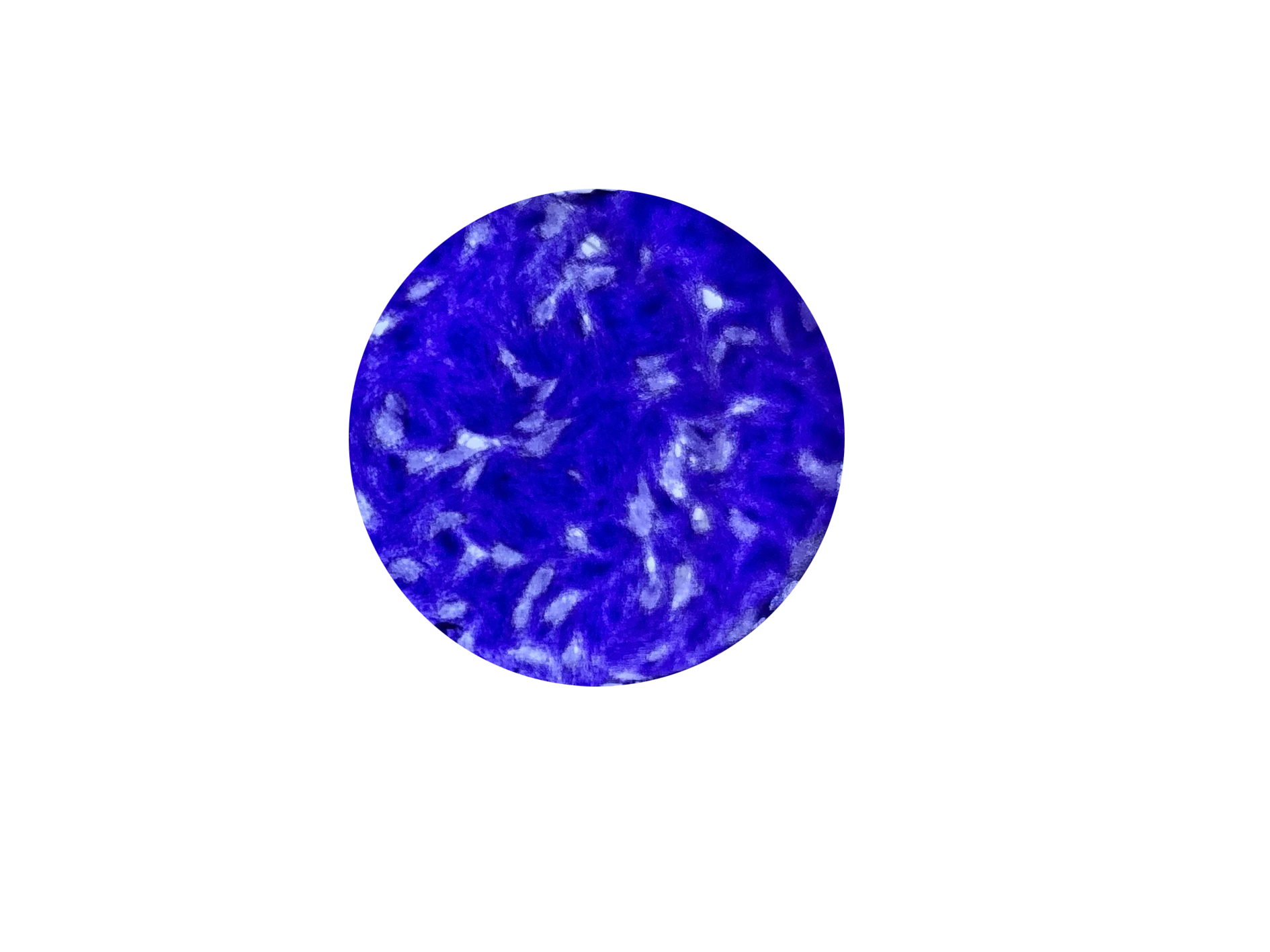

Supplement: Supplementary file 1 [file DataSheet3.ZIP › Others/plaque assay/ctd3.png]

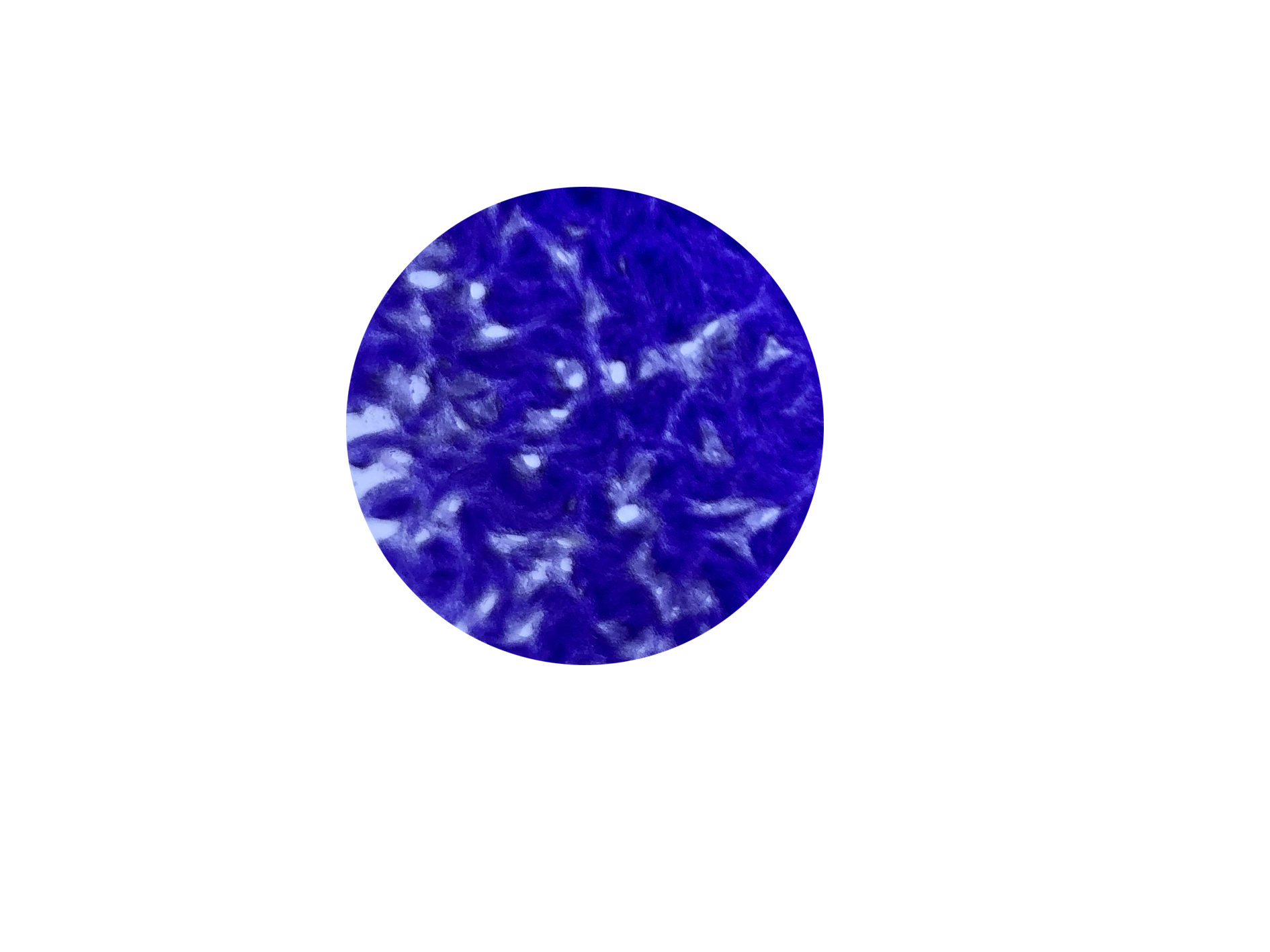

Supplement: Supplementary file 1 [file DataSheet3.ZIP › Others/plaque assay/efpp.png]

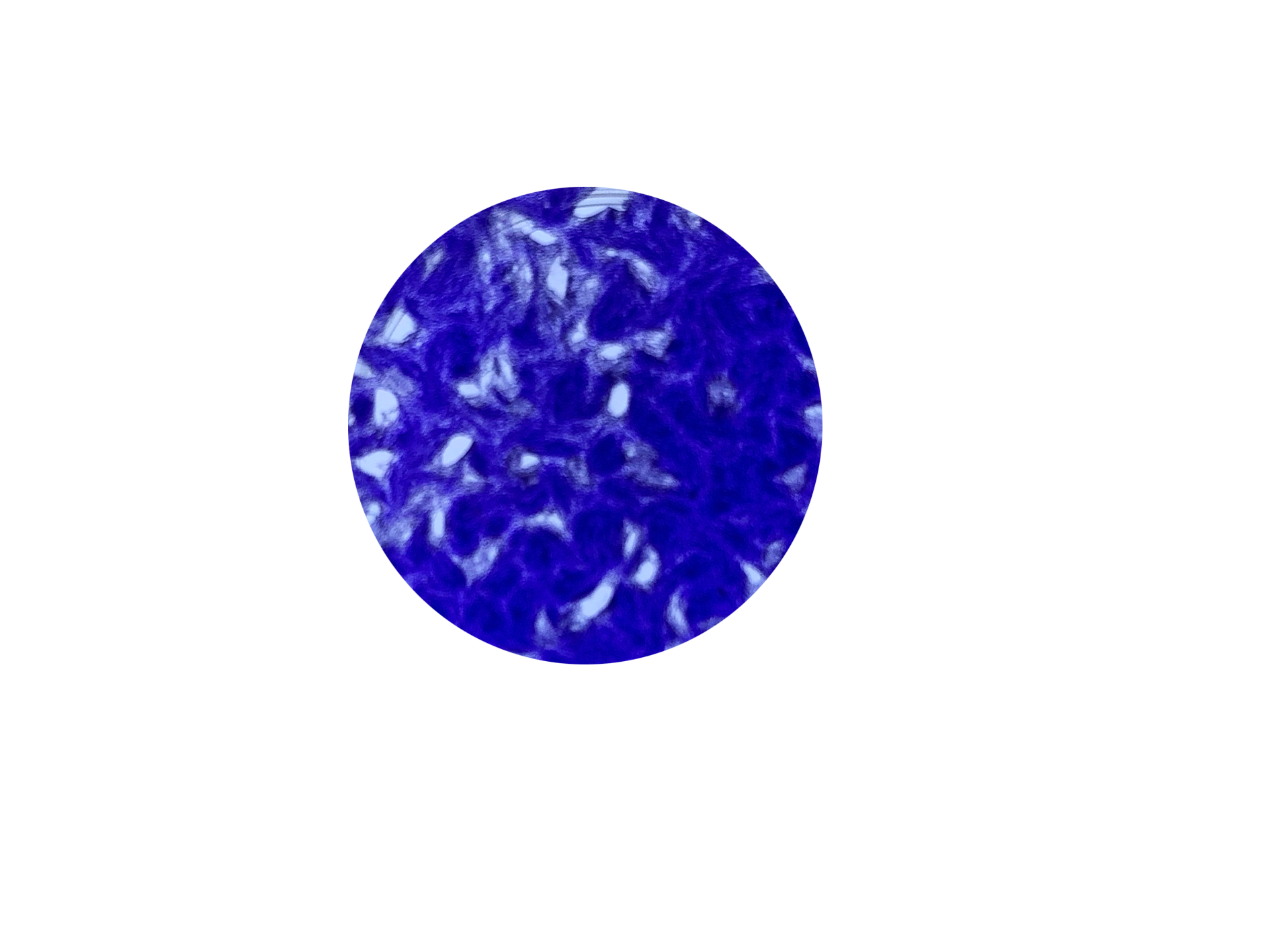

Supplement: Supplementary file 1 [file DataSheet3.ZIP › Others/plaque assay/pp5.png]

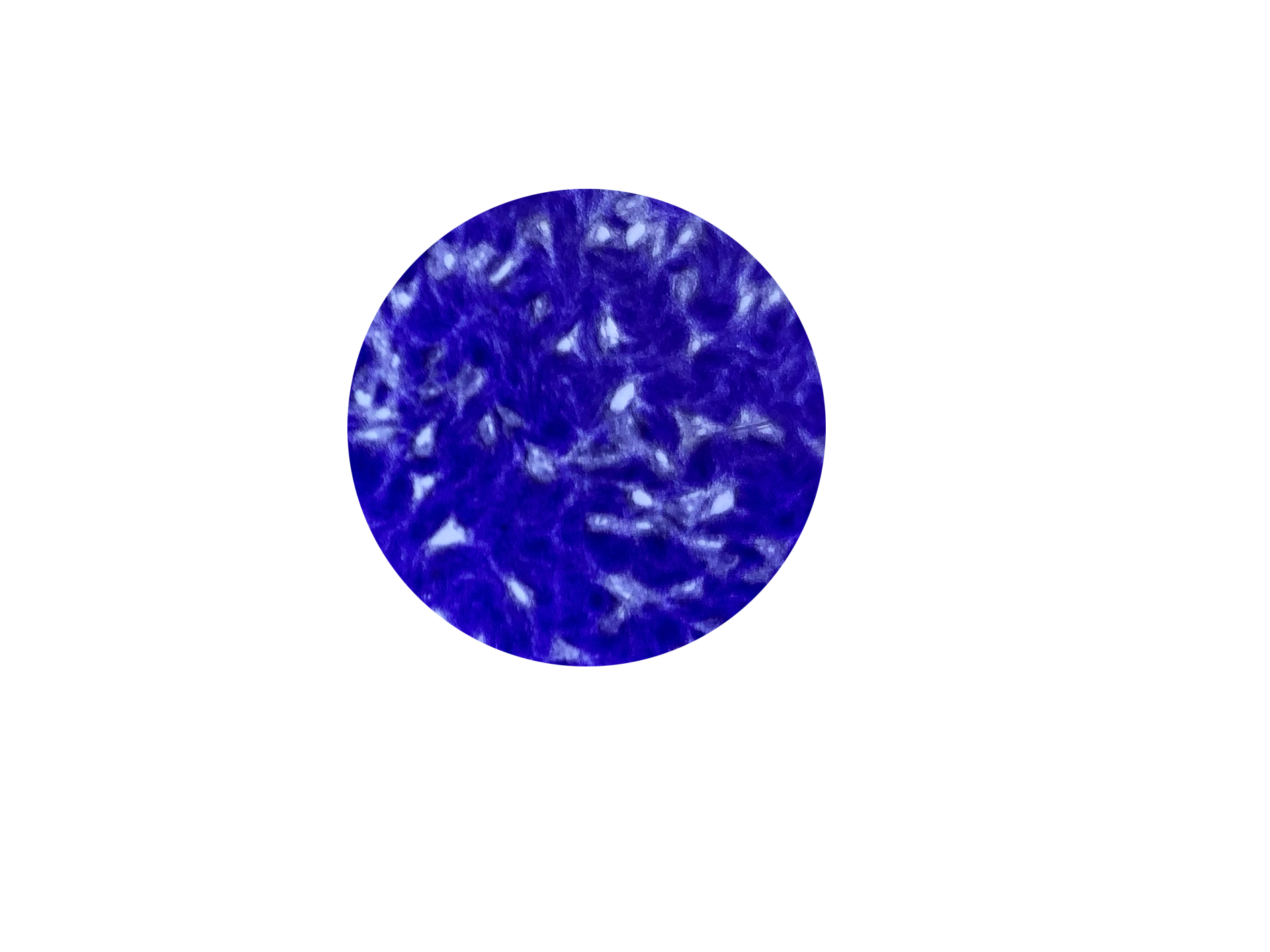

Supplement: Supplementary file 1 [file DataSheet3.ZIP › Others/plaque assay/pp7.png]

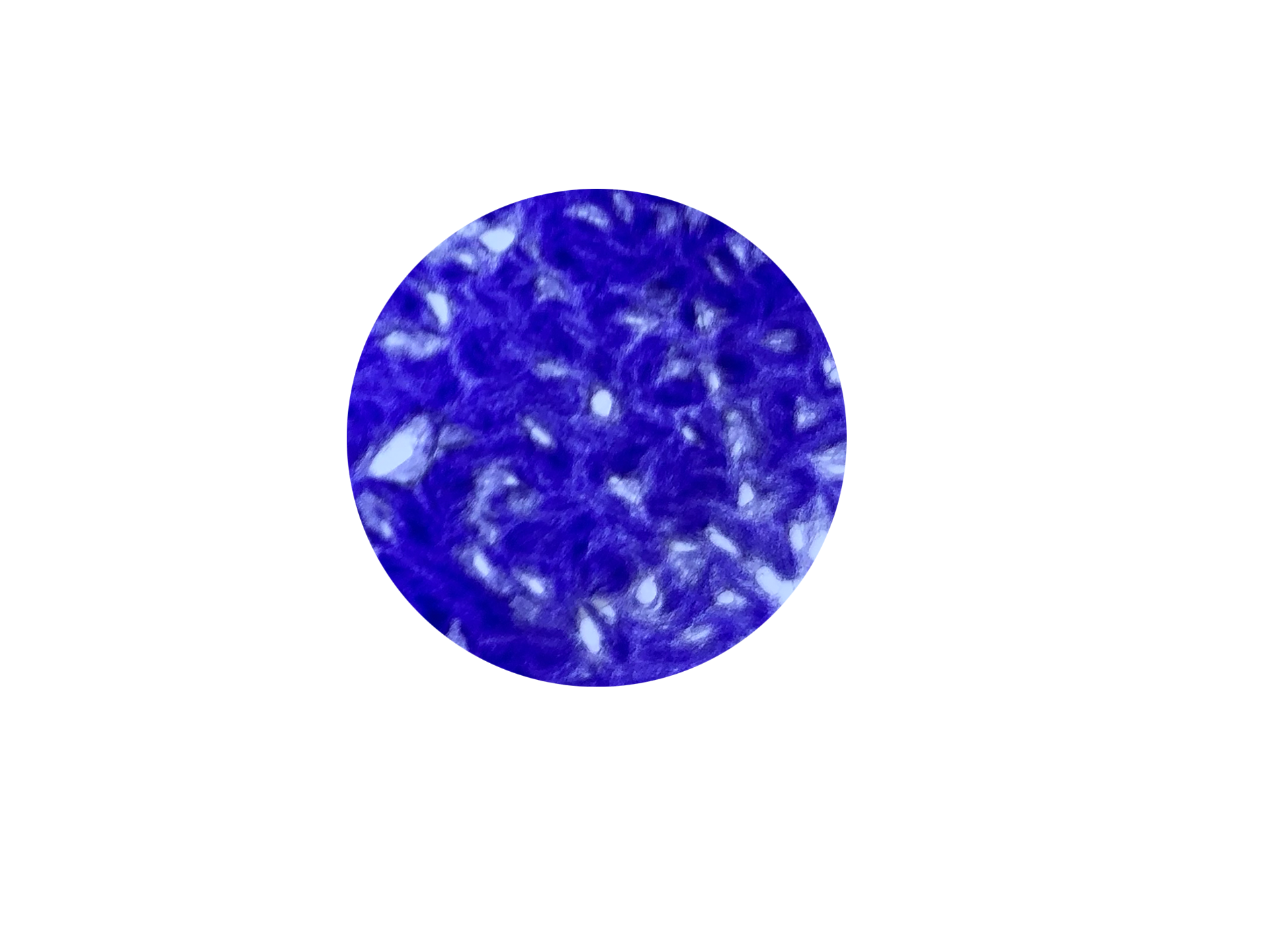

Supplement: Supplementary file 1 [file DataSheet3.ZIP › Others/plaque assay/ppm12.png]

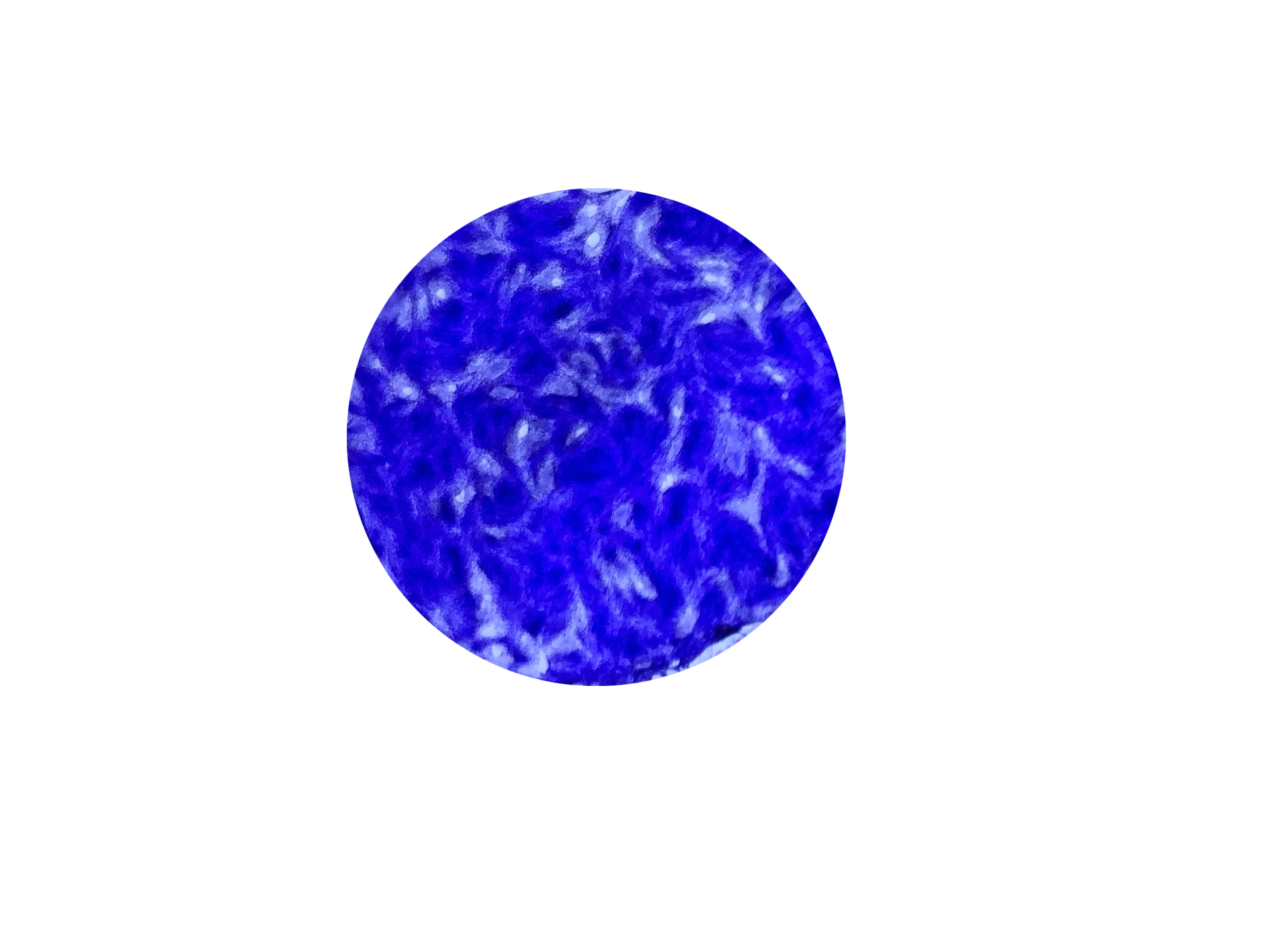

Supplement: Supplementary file 1 [file DataSheet3.ZIP › Others/plaque assay/ppm14.png]

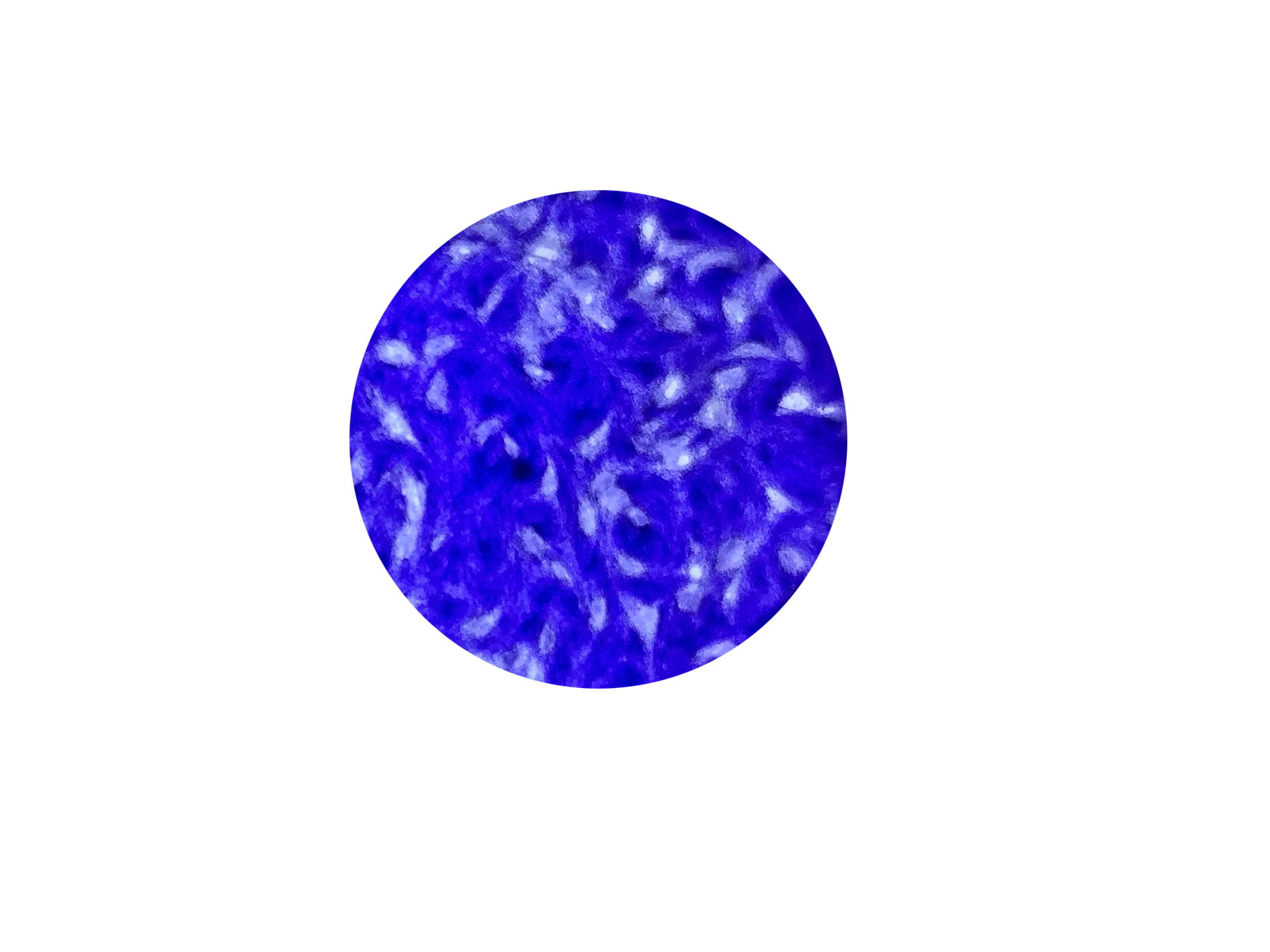

Supplement: Supplementary file 1 [file DataSheet3.ZIP › Others/plaque assay/ppm18.png]

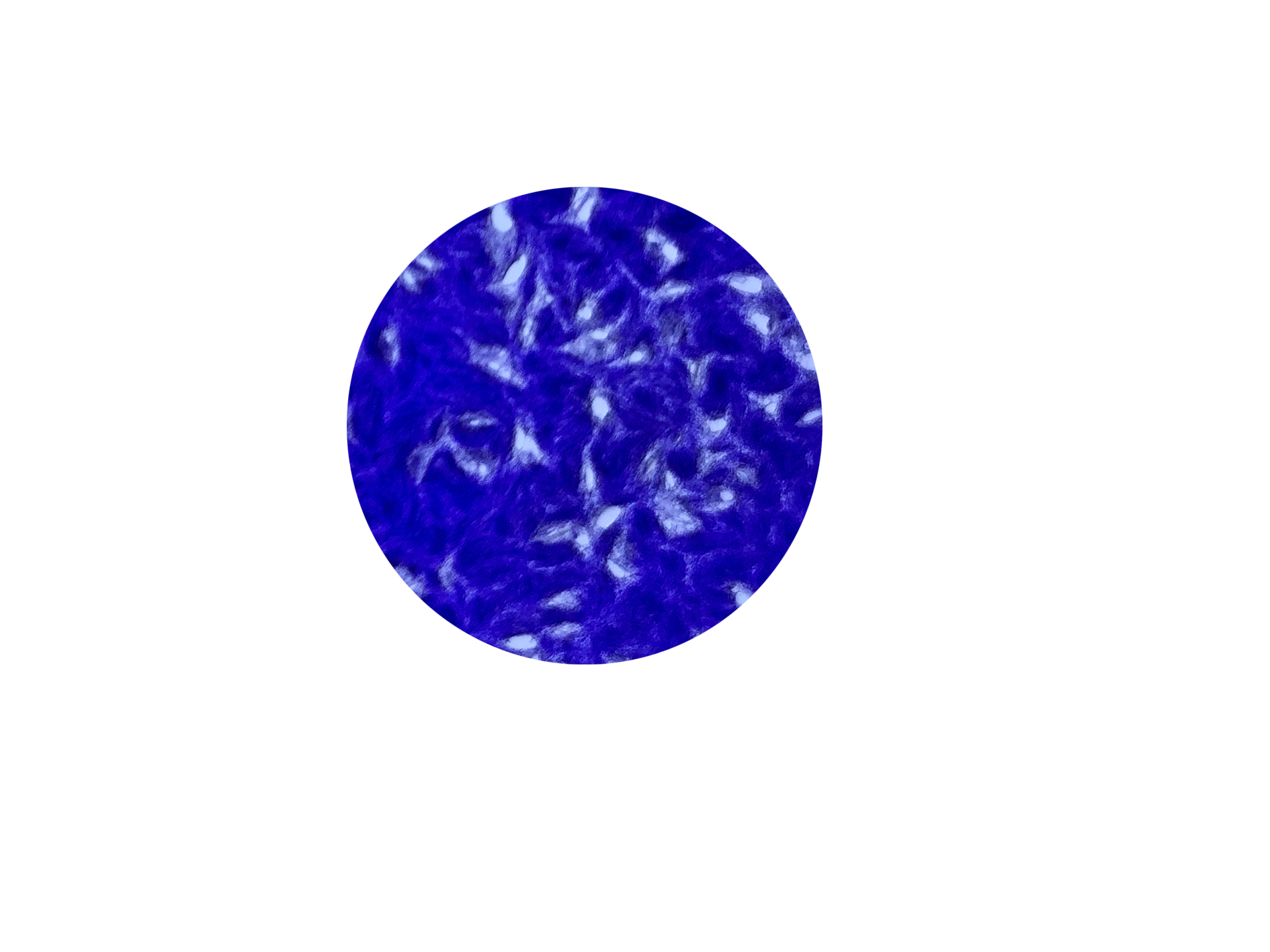

Supplement: Supplementary file 1 [file DataSheet3.ZIP › Others/plaque assay/ppm3f.png]

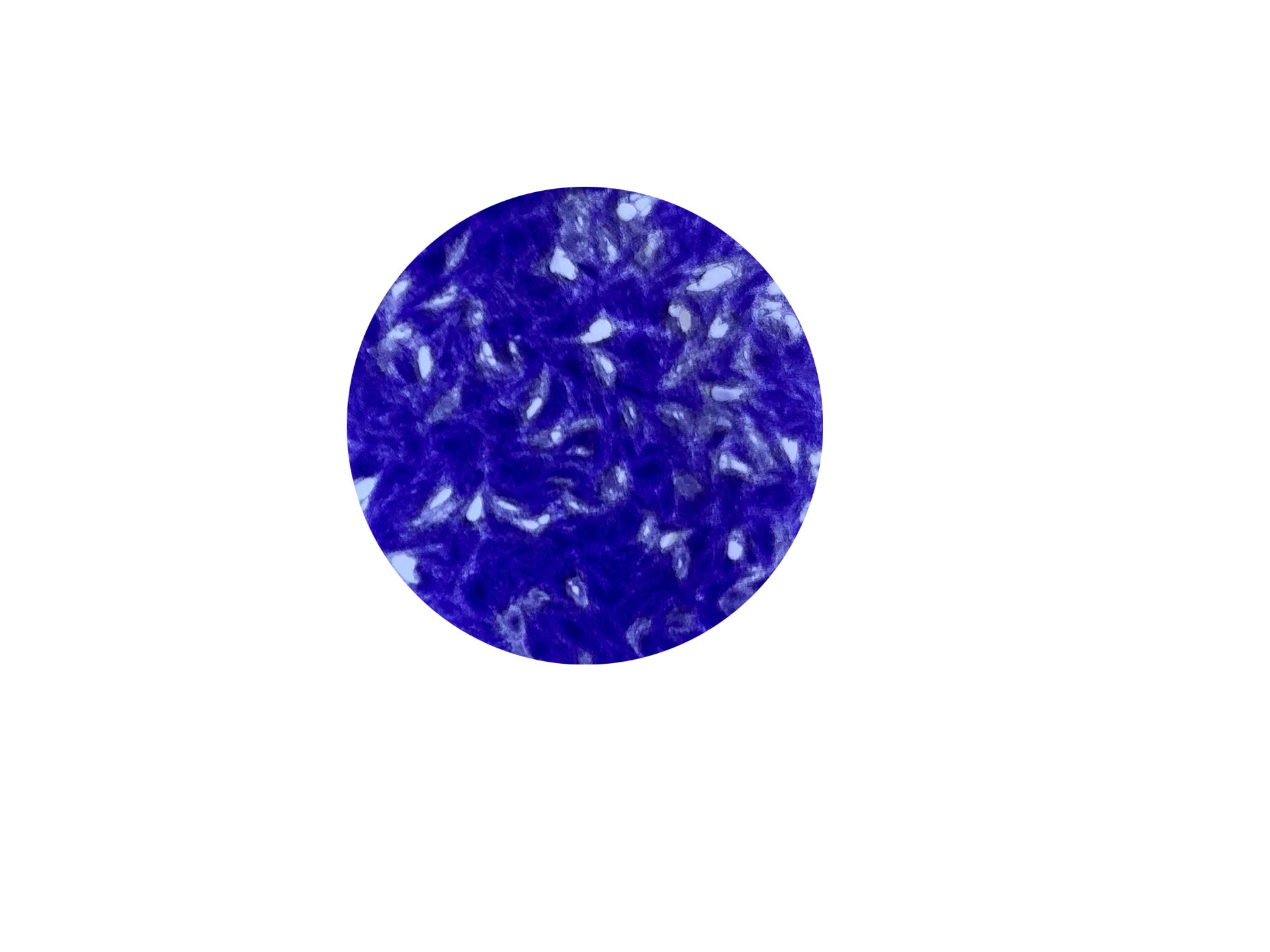

Supplement: Supplementary file 1 [file DataSheet3.ZIP › Others/plaque assay/ppm4.png]

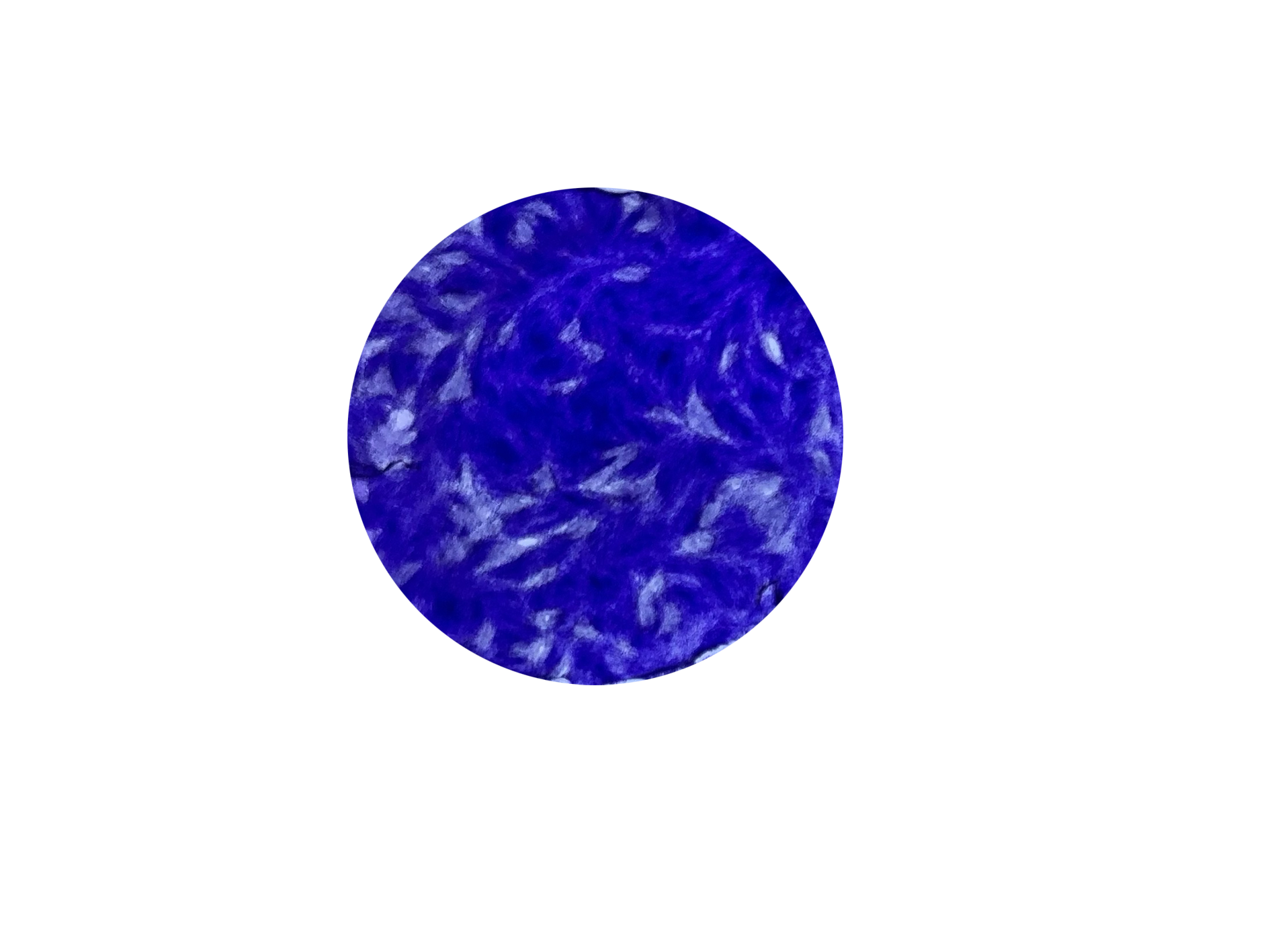

Supplement: Supplementary file 1 [file DataSheet3.ZIP › Others/plaque assay/ppm5a.png]

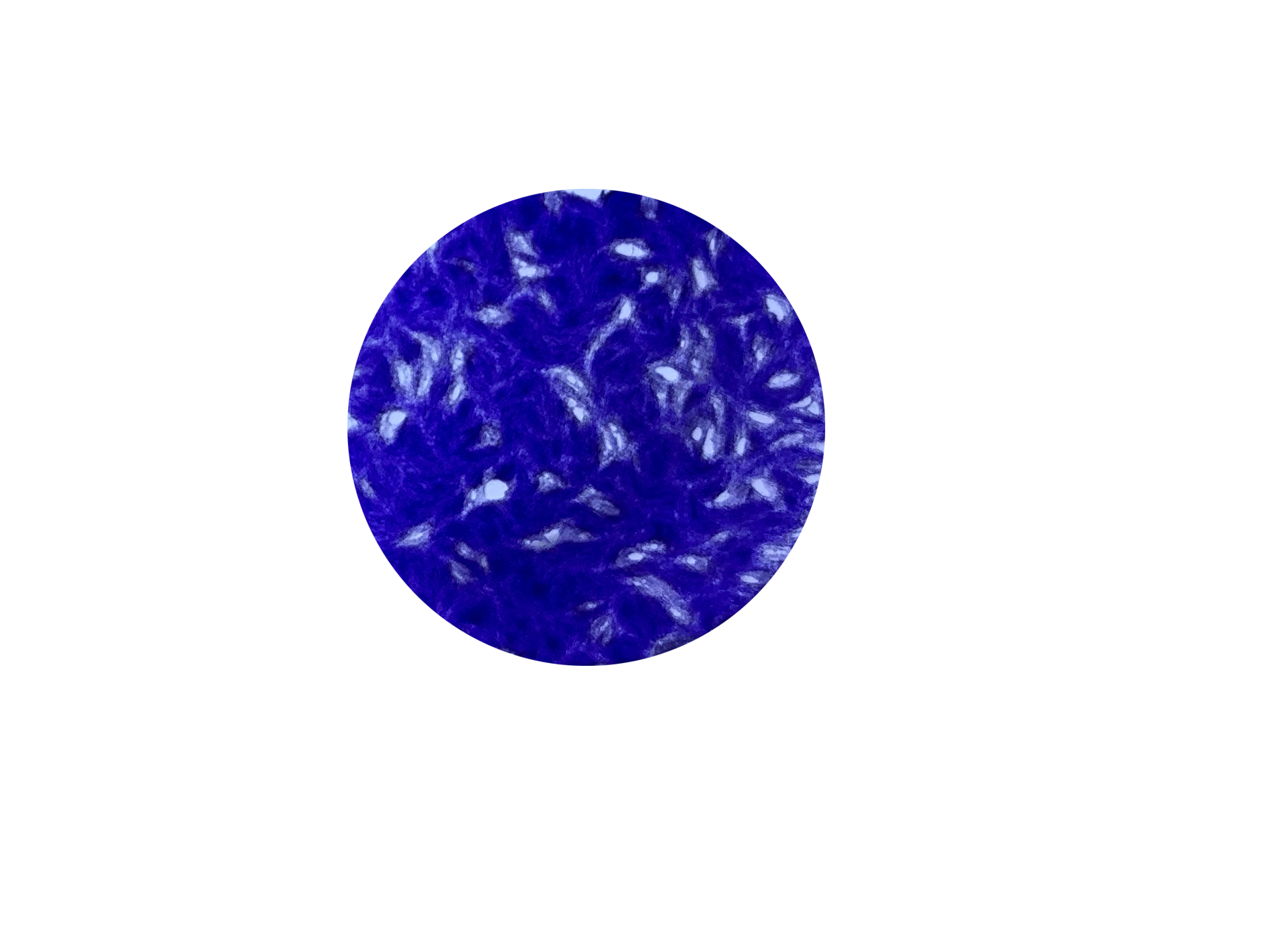

Supplement: Supplementary file 1 [file DataSheet3.ZIP › Others/plaque assay/ppm5b.png]

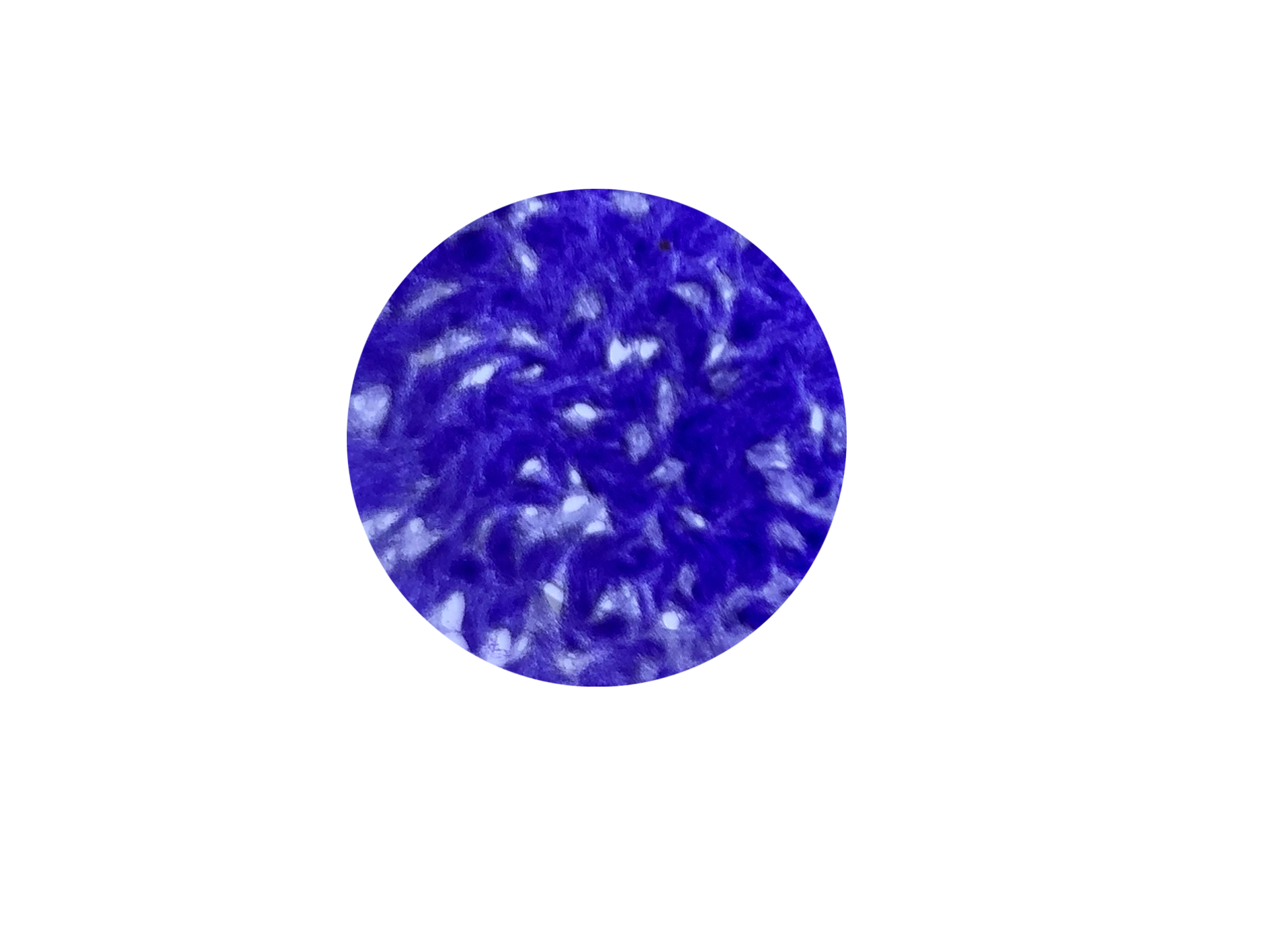

Supplement: Supplementary file 1 [file DataSheet3.ZIP › Others/plaque assay/ppm6.png]

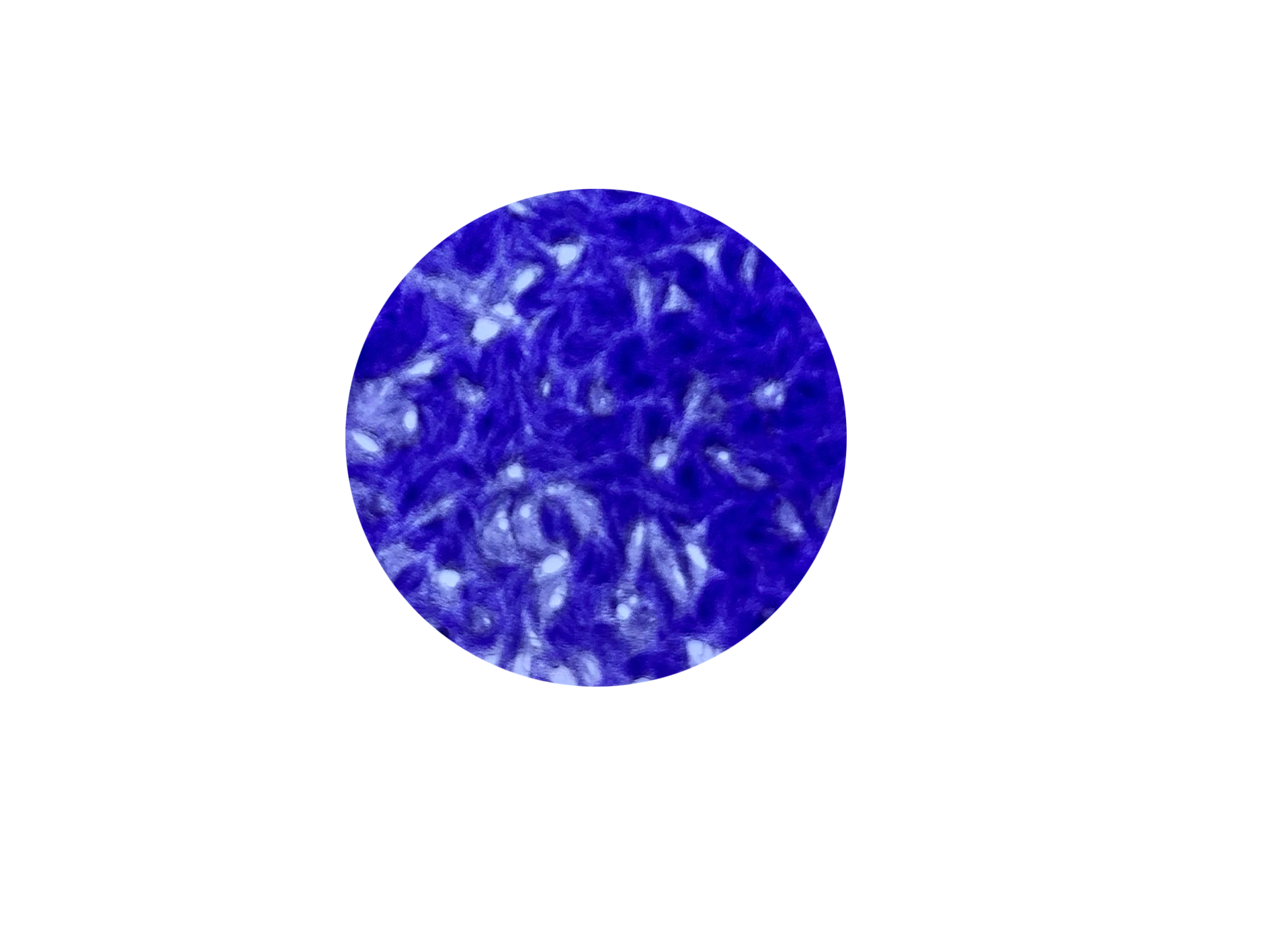

Supplement: Supplementary file 1 [file DataSheet3.ZIP › Others/plaque assay/ppm8.png]

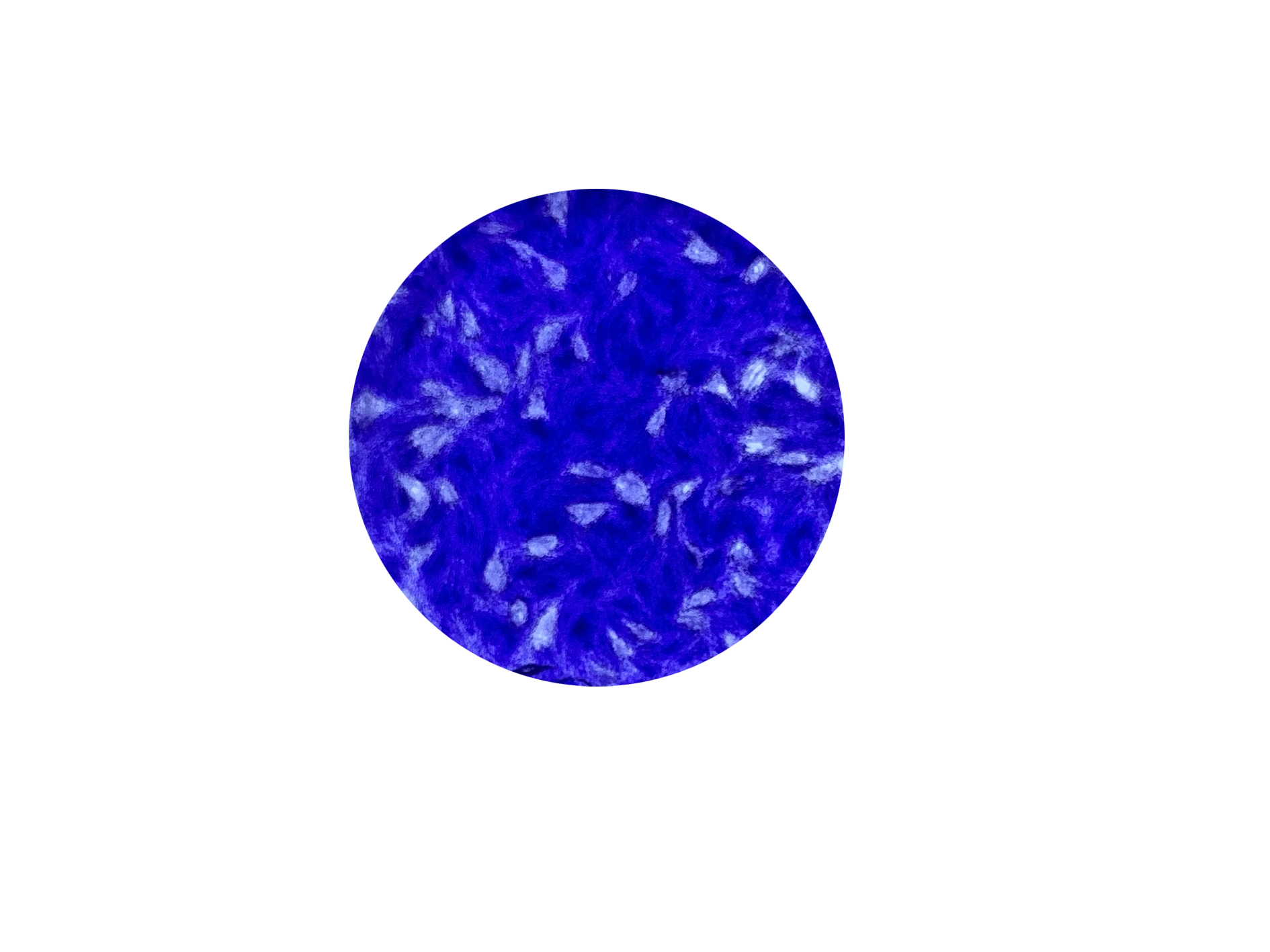

Supplement: Supplementary file 1 [file DataSheet3.ZIP › Others/plaque assay/ppm9.png]

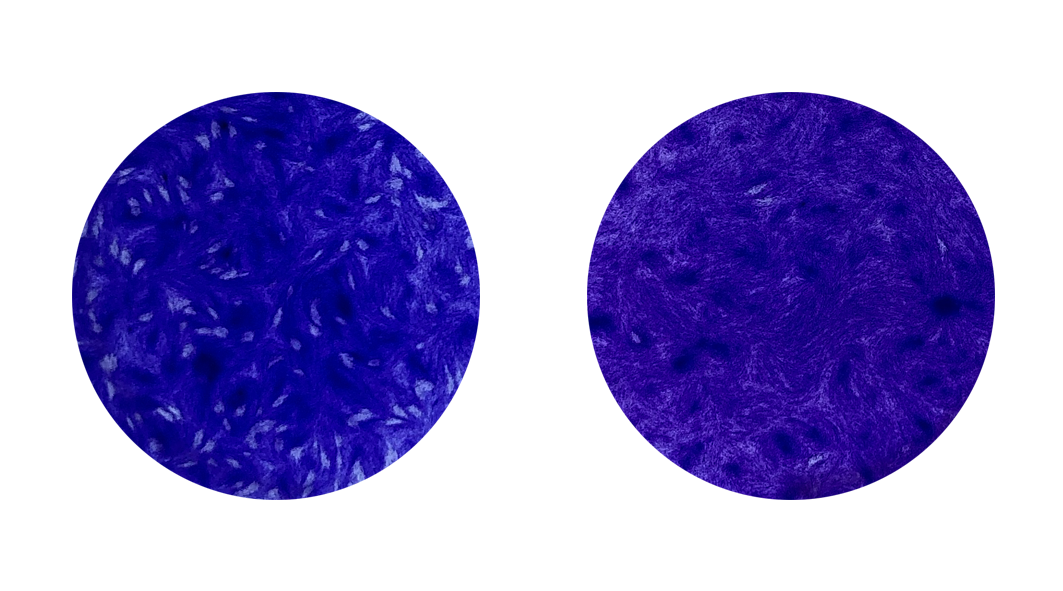

Supplement: Supplementary file 1 [file DataSheet3.ZIP › Others/plaque assay/pru-pp7-500.png]

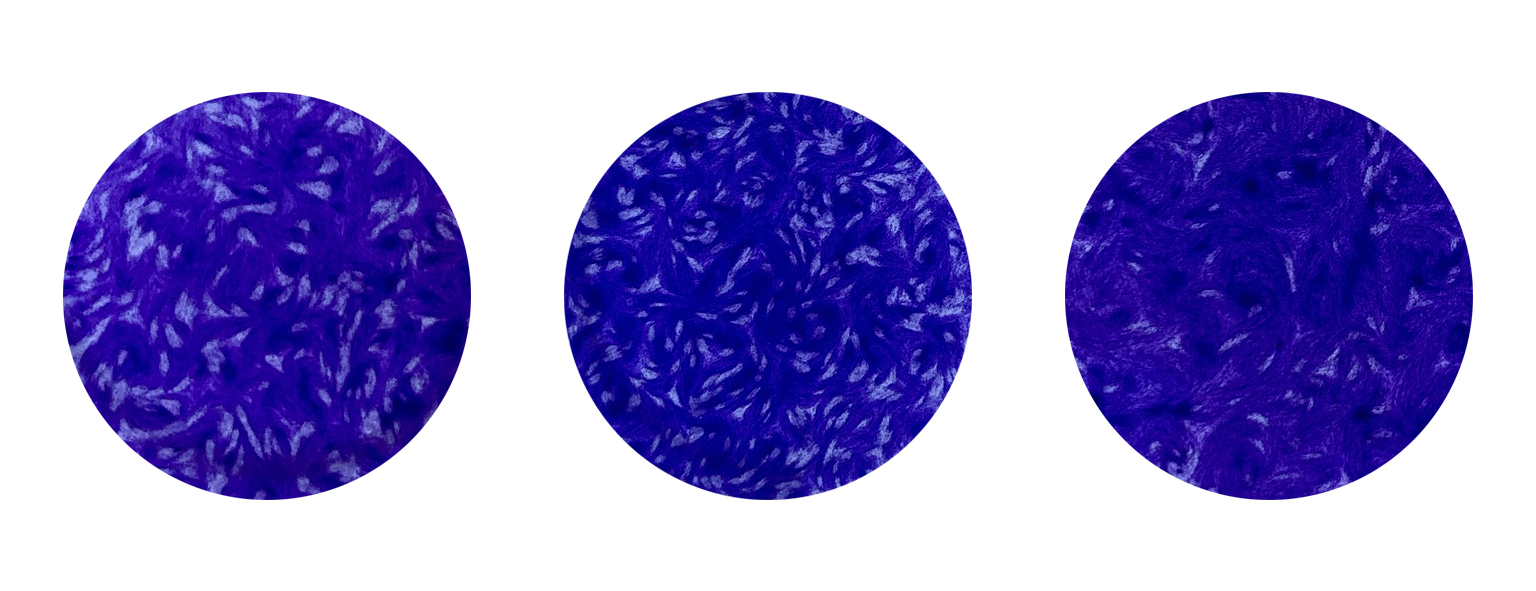

Supplement: Supplementary file 1 [file DataSheet3.ZIP › Others/plaque assay/pru-slp-pp7-5000.png]

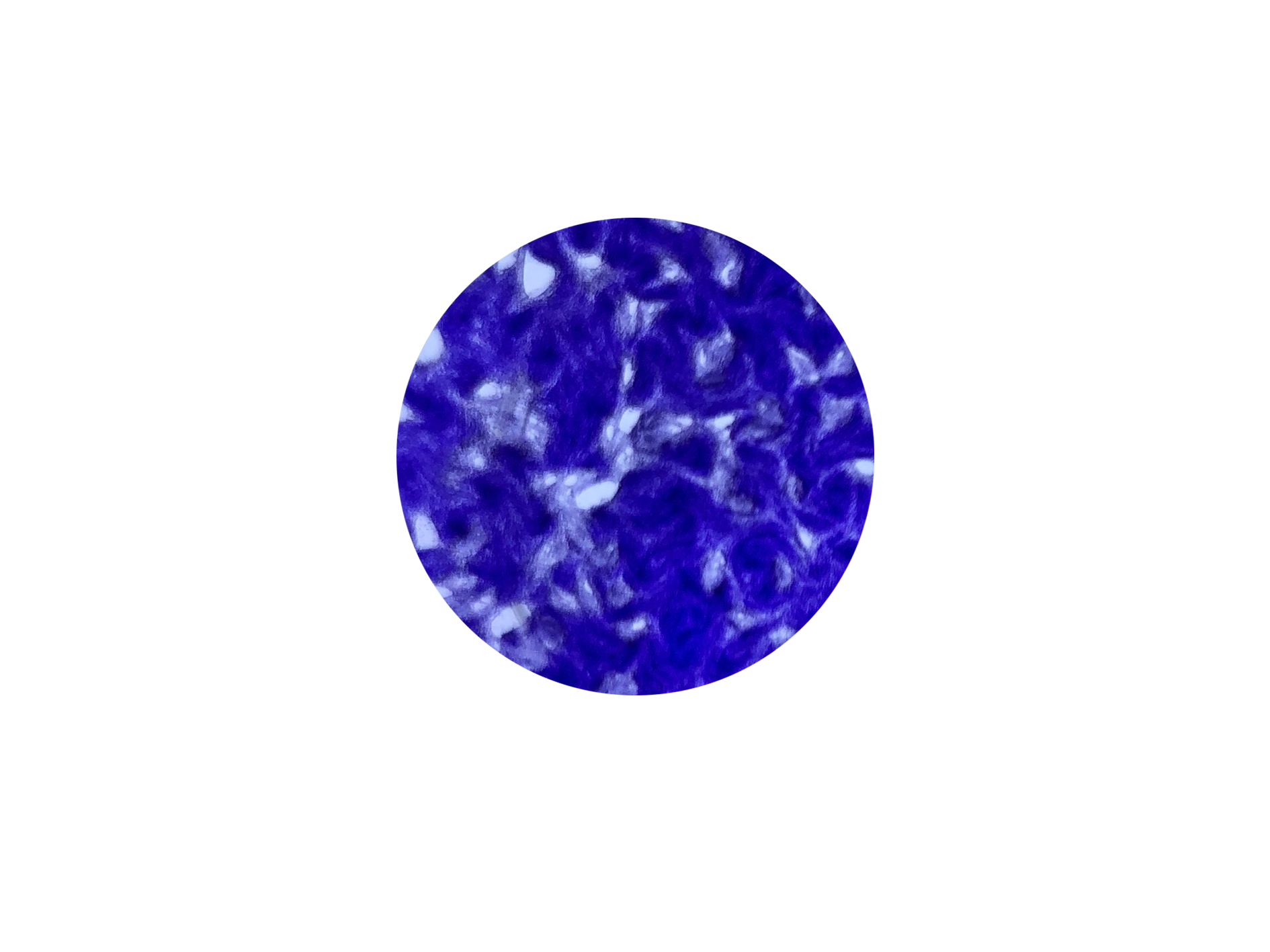

Supplement: Supplementary file 1 [file DataSheet3.ZIP › Others/plaque assay/RH.png]

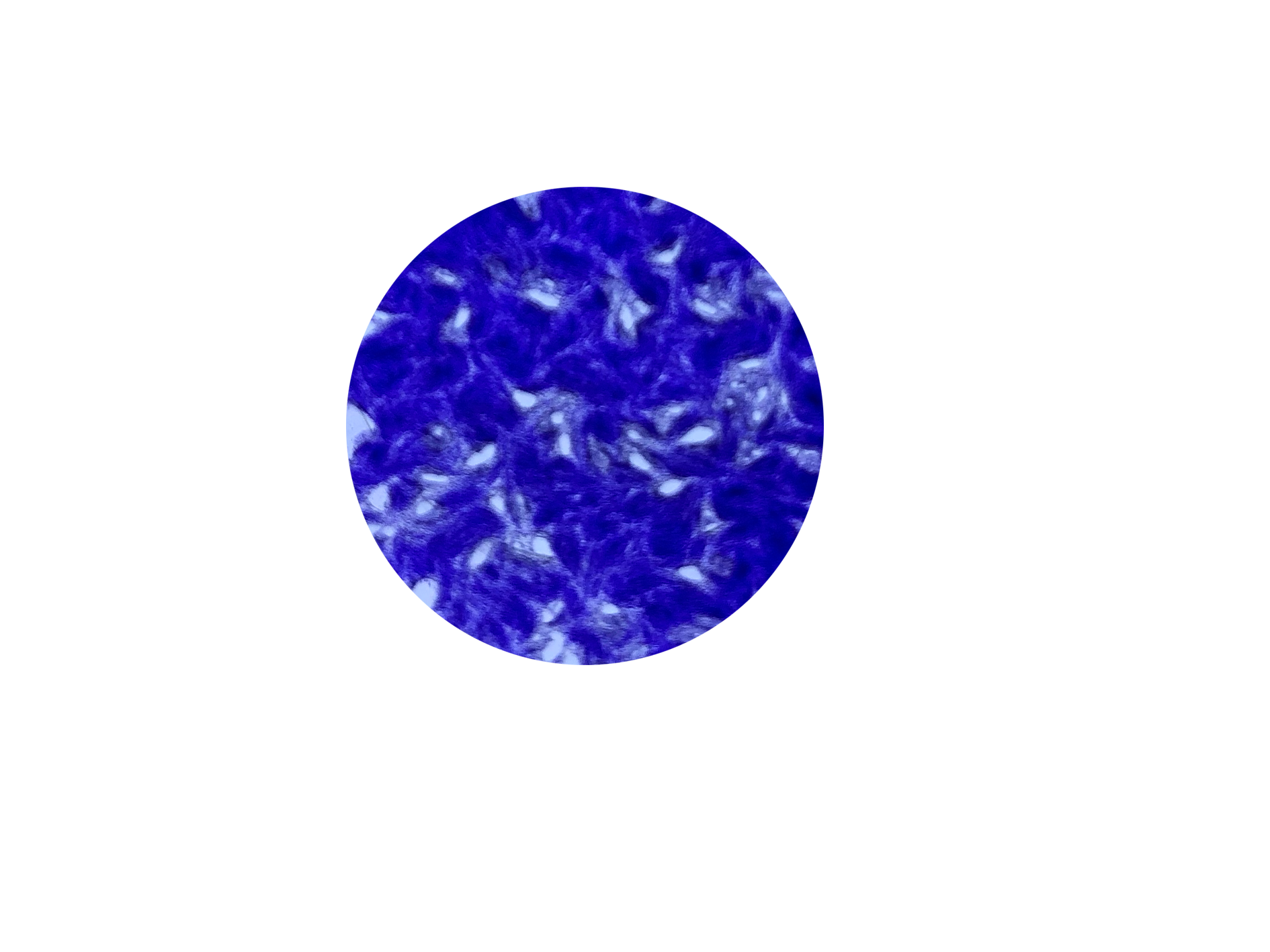

Supplement: Supplementary file 1 [file DataSheet3.ZIP › Others/plaque assay/slp.png]

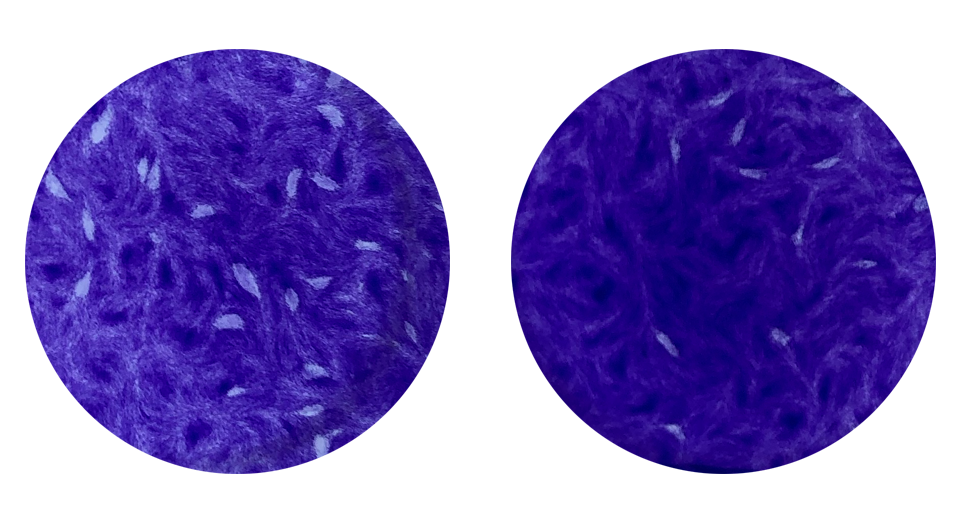

Supplement: Supplementary file 1 [file DataSheet3.ZIP › Others/plaque assay/WT-PP7.png]

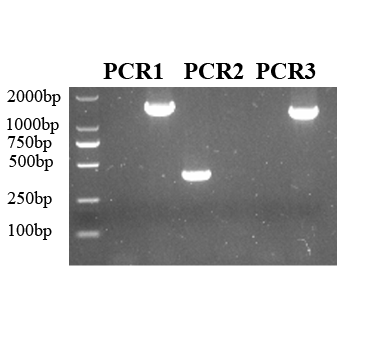

Supplement: Supplementary file 1 [file DataSheet3.ZIP › Others/Raw data/pp7-pru.tif]

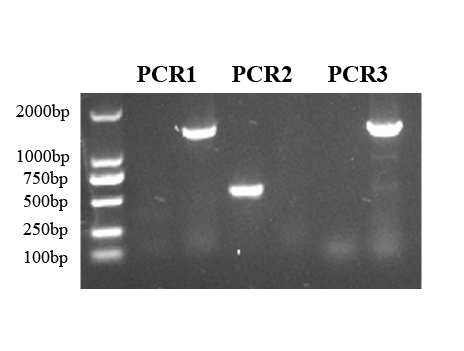

Supplement: Supplementary file 1 [file DataSheet3.ZIP › Others/Raw data/slp-pru.tif]

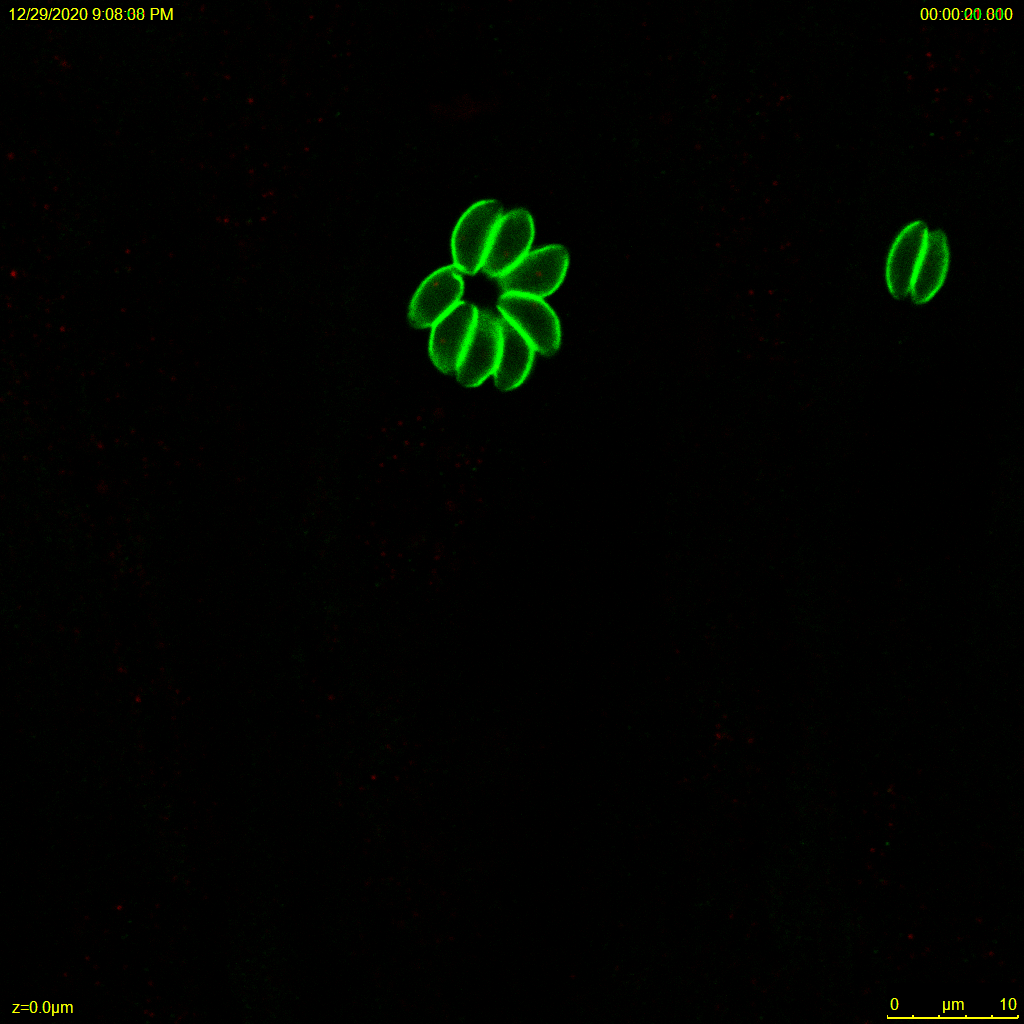

Supplement: Supplementary file 2 [file DataSheet1.ZIP › Figure 1/Composite-WT.tif]

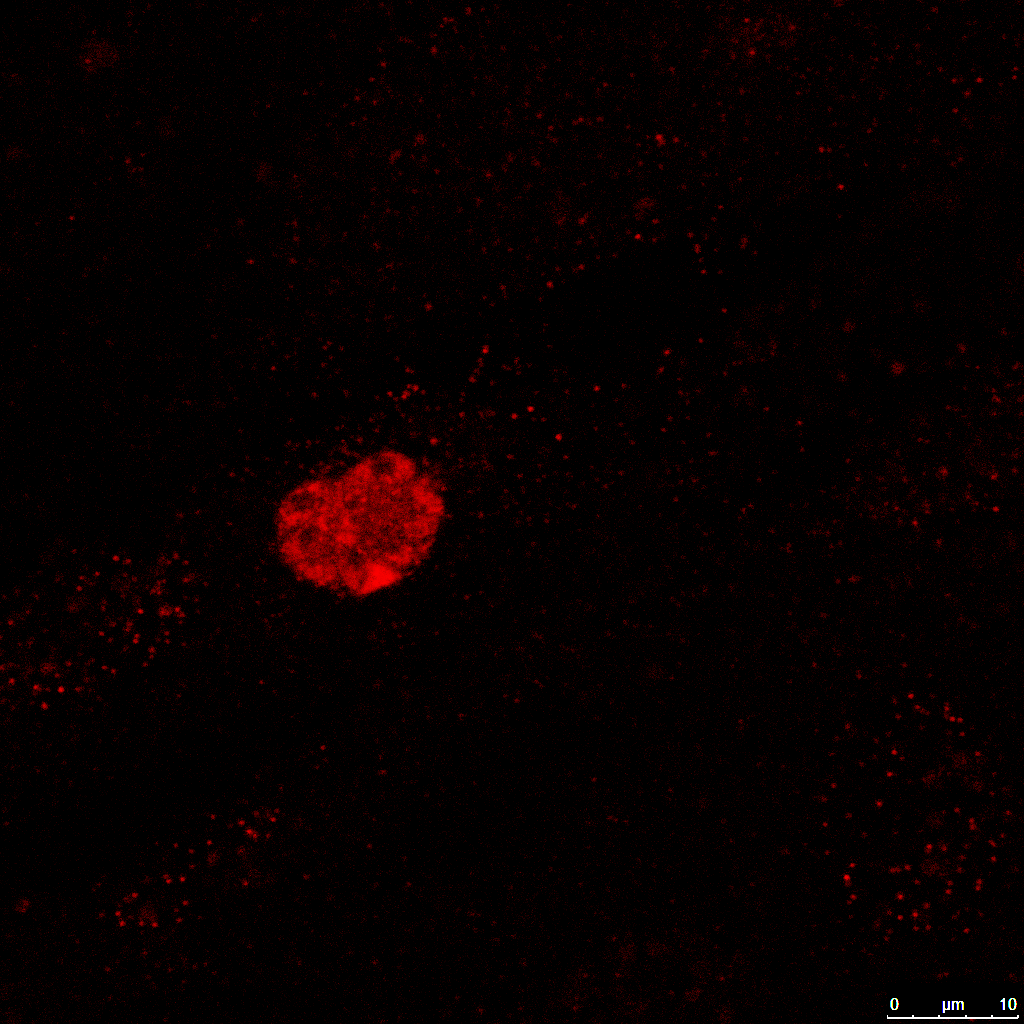

Supplement: Supplementary file 2 [file DataSheet1.ZIP › Figure 1/ctd2-ha.png]

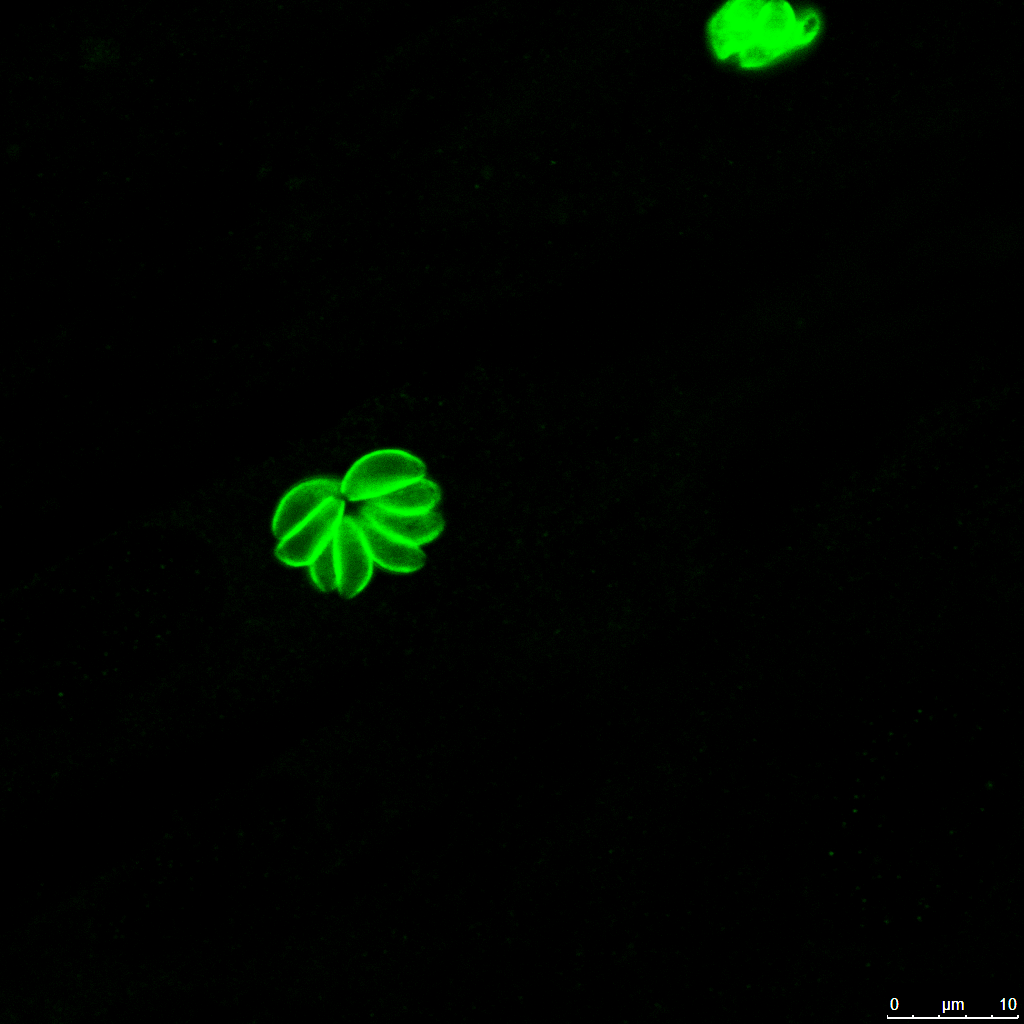

Supplement: Supplementary file 2 [file DataSheet1.ZIP › Figure 1/ctd2-imc.png]

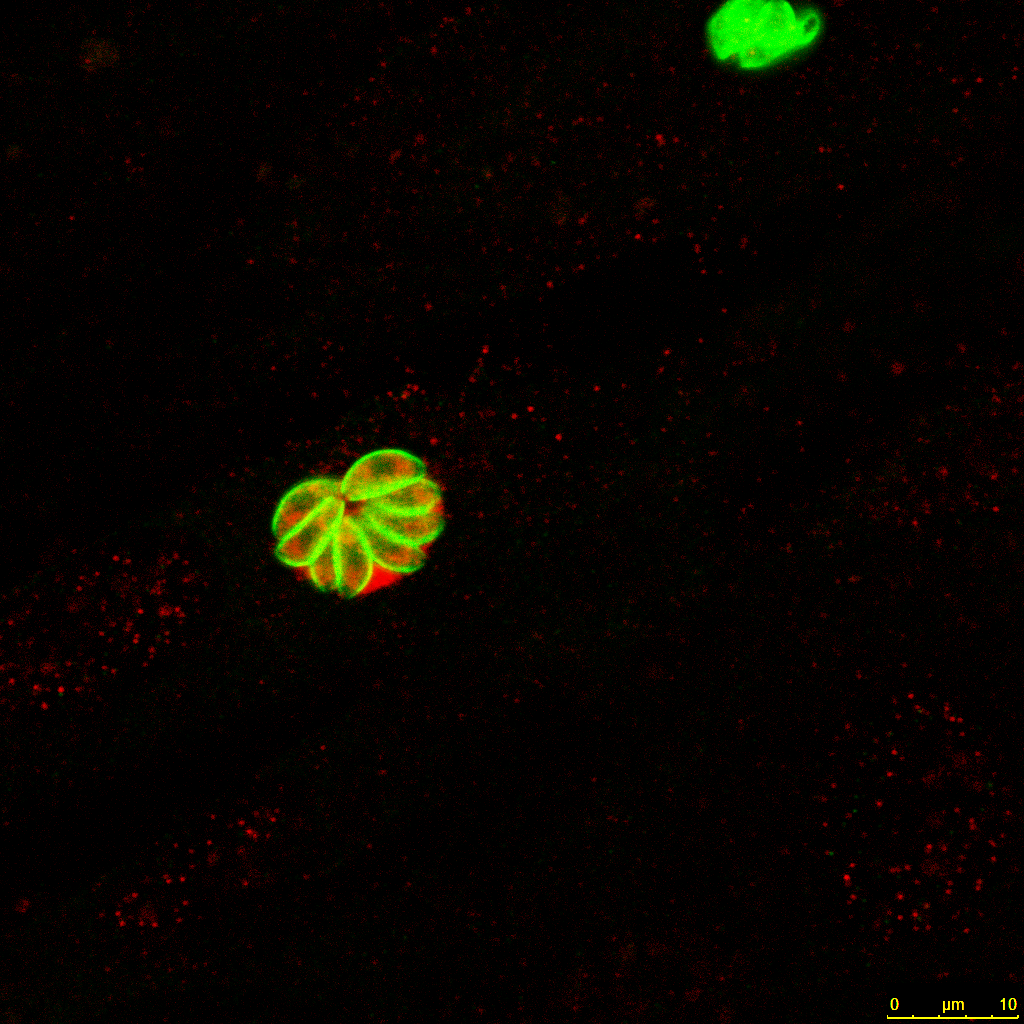

Supplement: Supplementary file 2 [file DataSheet1.ZIP › Figure 1/ctd2-merge.tif]

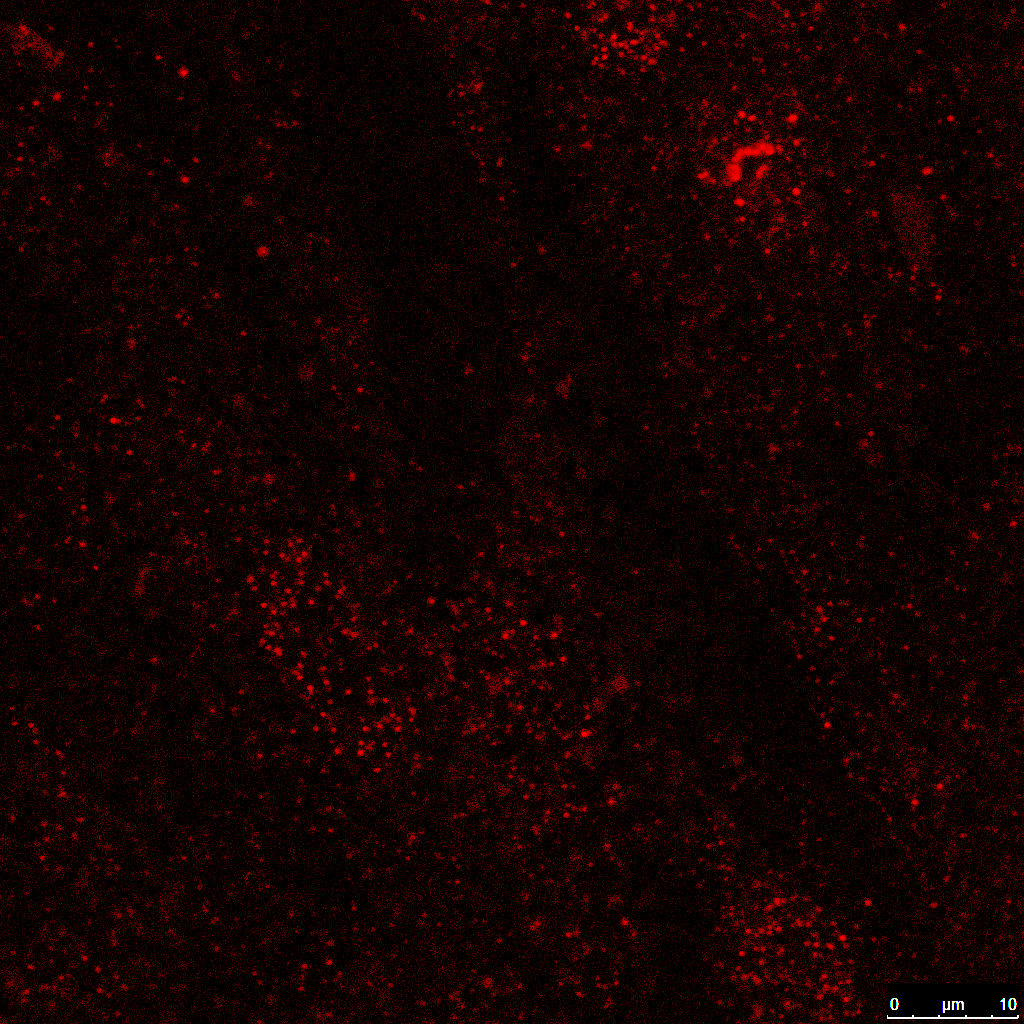

Supplement: Supplementary file 2 [file DataSheet1.ZIP › Figure 1/ctd3-ha.png]

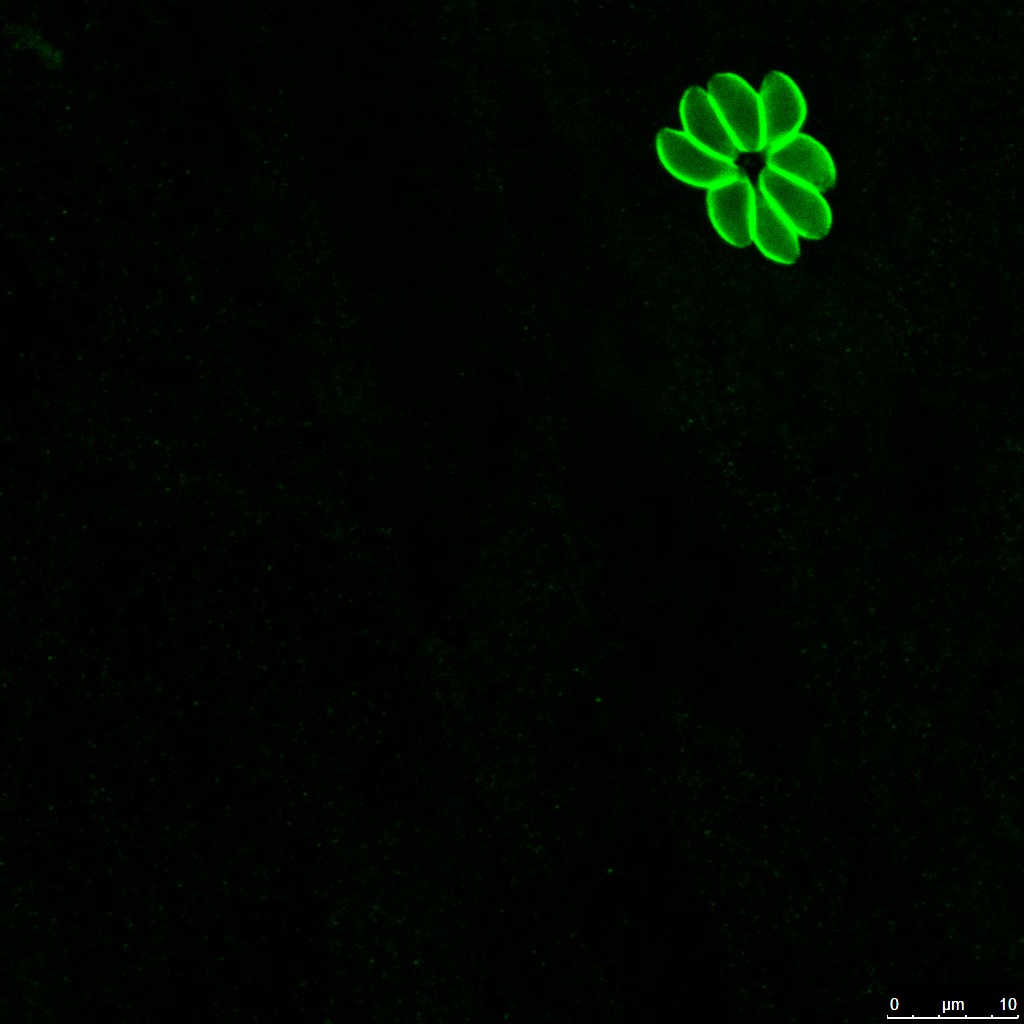

Supplement: Supplementary file 2 [file DataSheet1.ZIP › Figure 1/ctd3-imc.png]

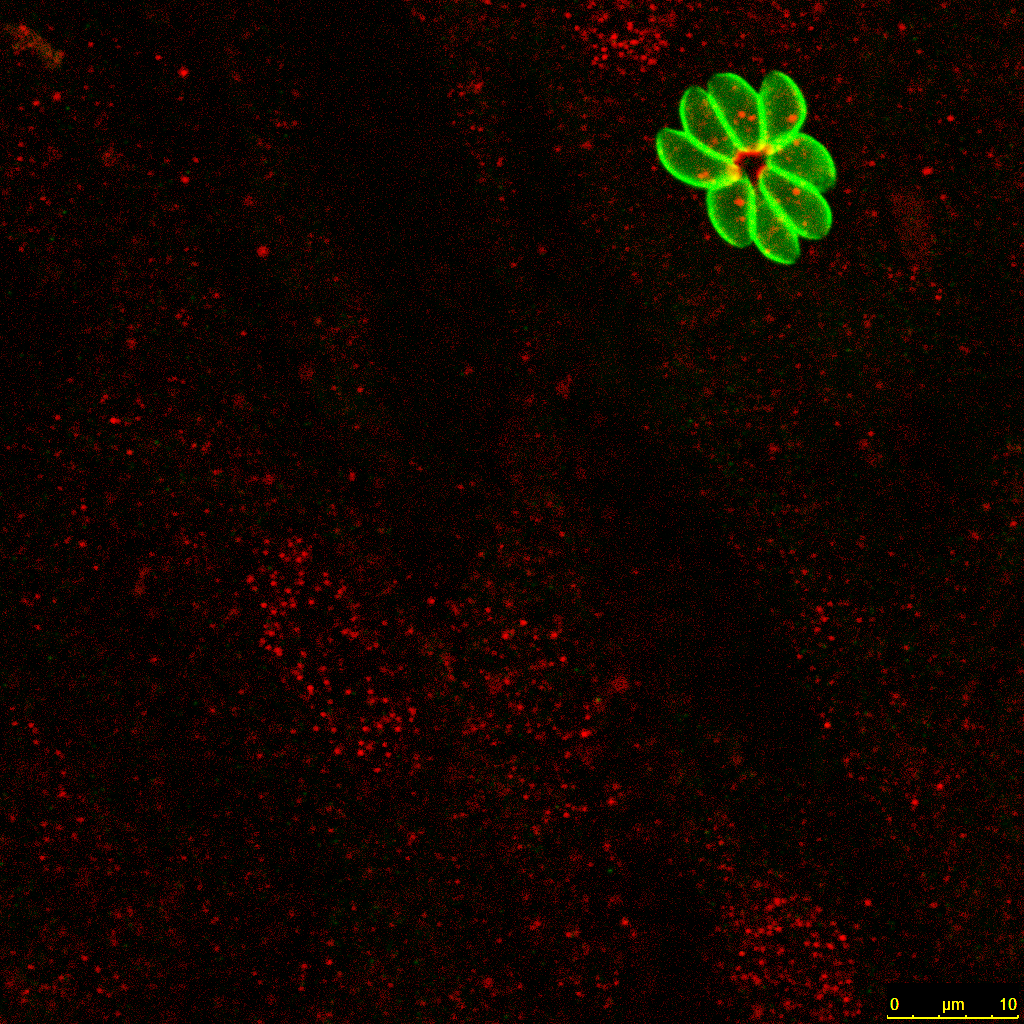

Supplement: Supplementary file 2 [file DataSheet1.ZIP › Figure 1/ctd3-merge.tif]

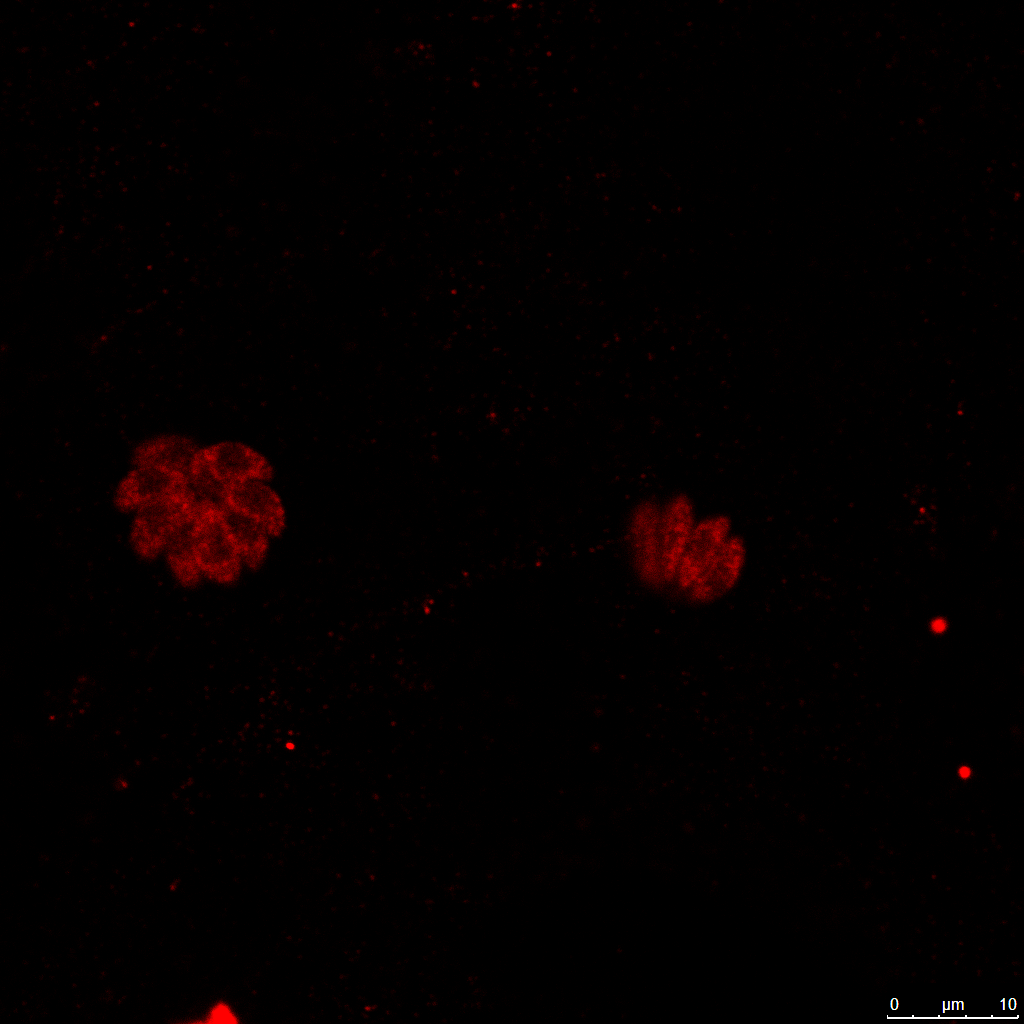

Supplement: Supplementary file 2 [file DataSheet1.ZIP › Figure 1/efpp-ha.png]

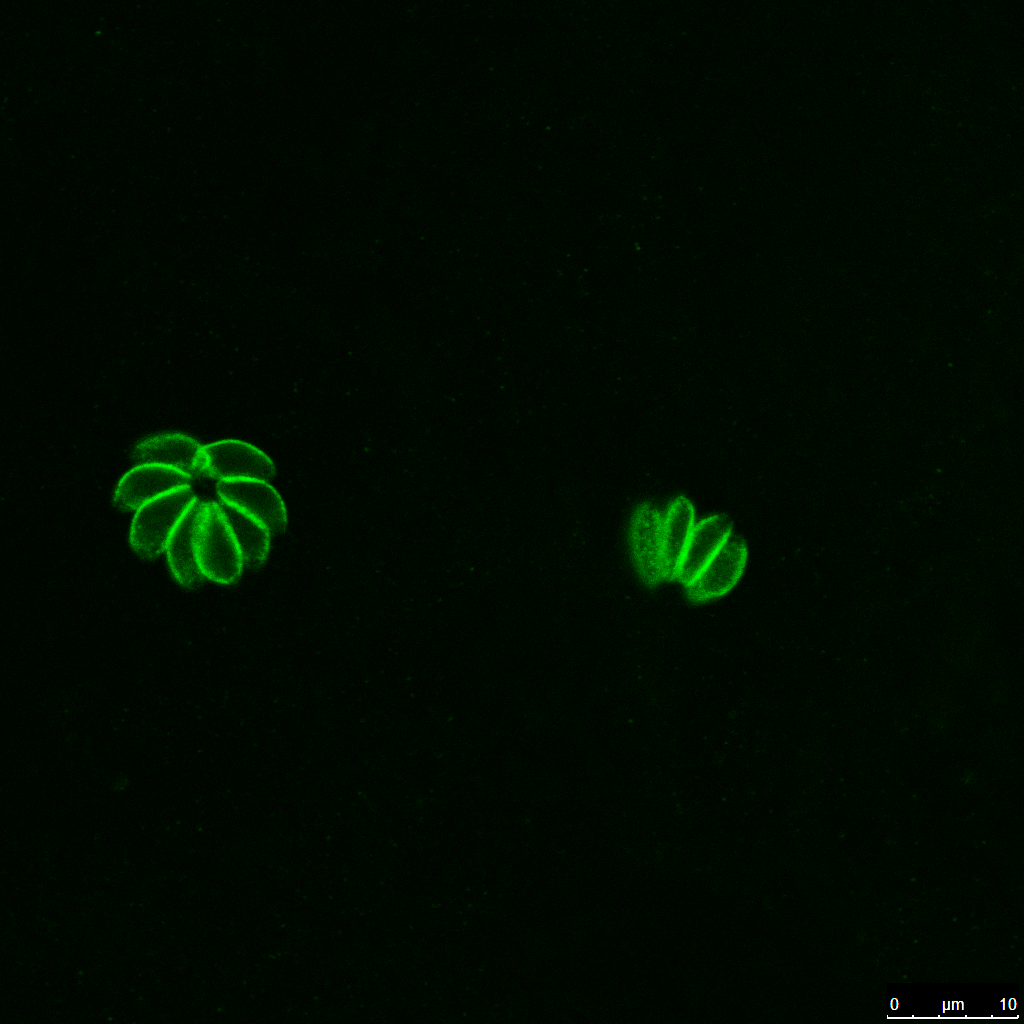

Supplement: Supplementary file 2 [file DataSheet1.ZIP › Figure 1/efpp-imc.png]

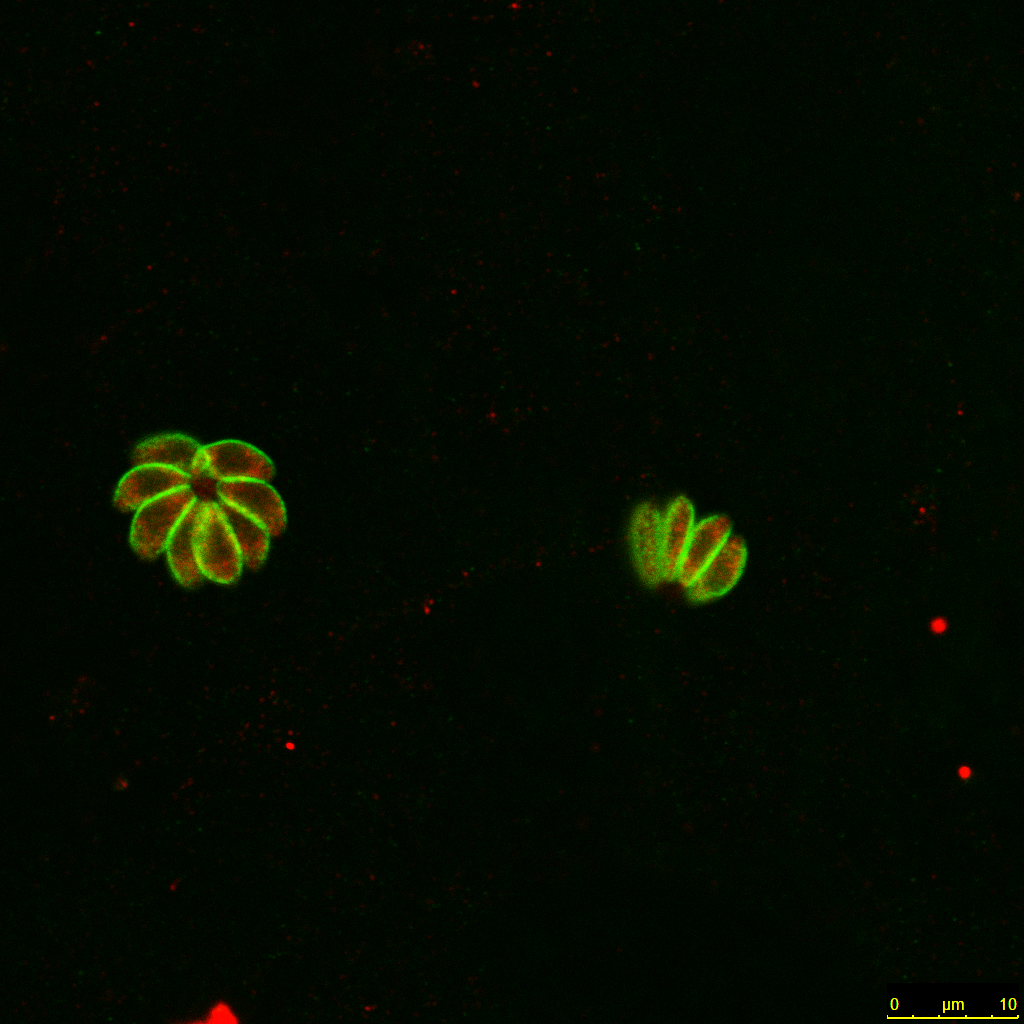

Supplement: Supplementary file 2 [file DataSheet1.ZIP › Figure 1/efpp-merge.tif]

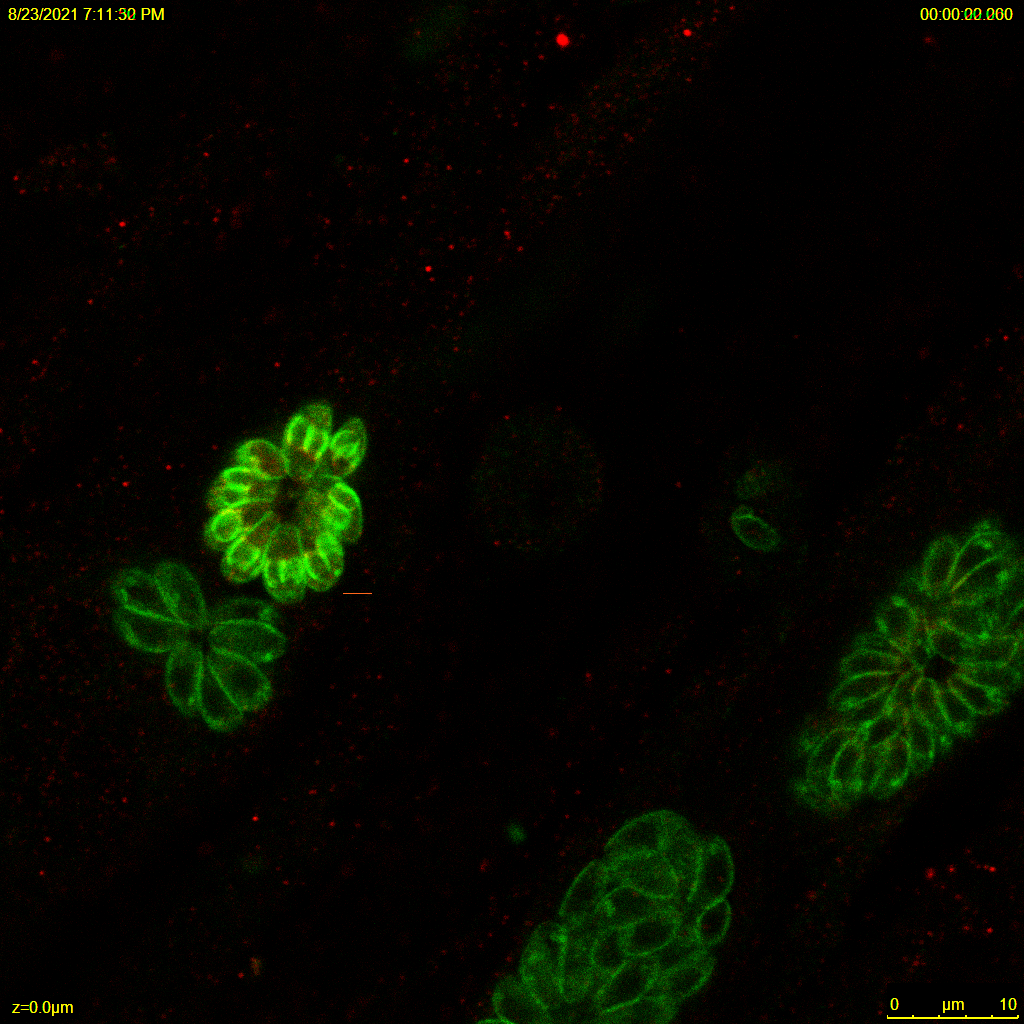

Supplement: Supplementary file 2 [file DataSheet1.ZIP › Figure 1/m12-1-Composite.tif]

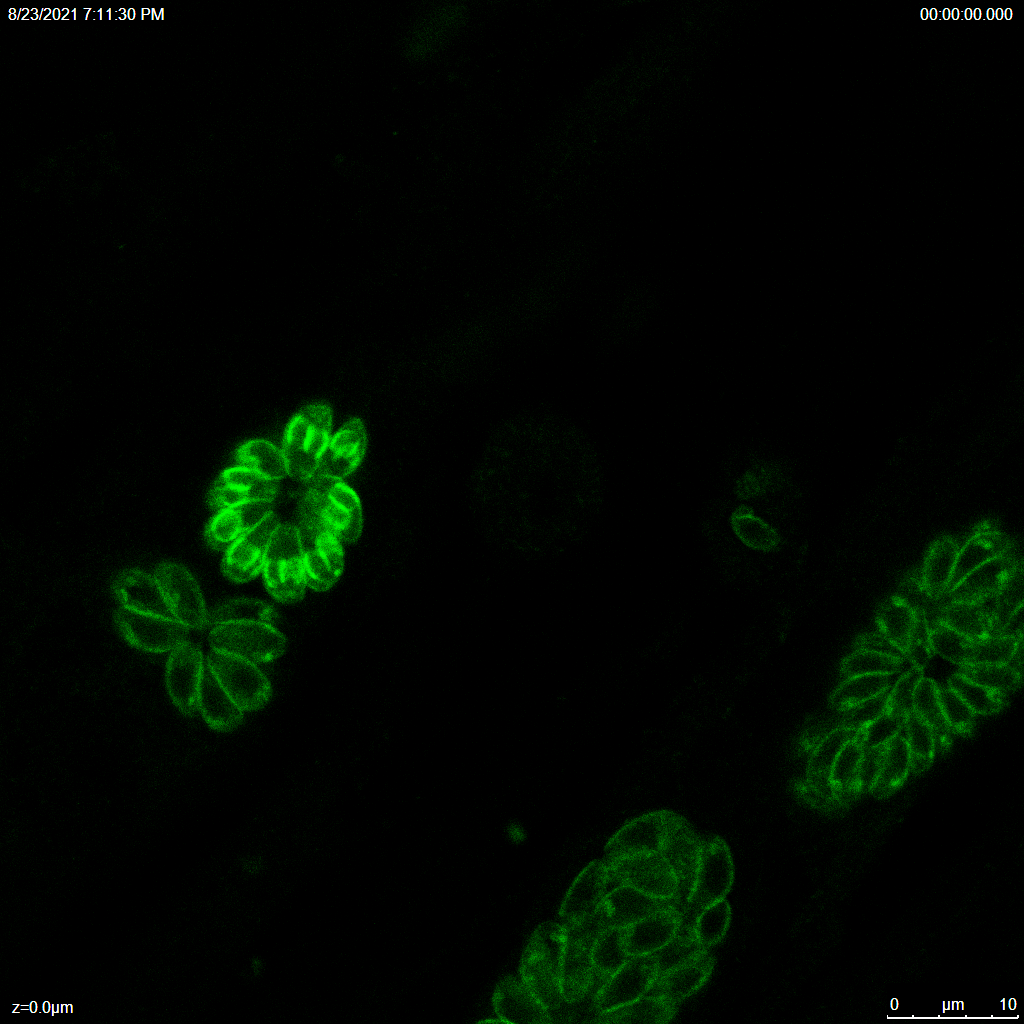

Supplement: Supplementary file 2 [file DataSheet1.ZIP › Figure 1/m12-1-green.png]

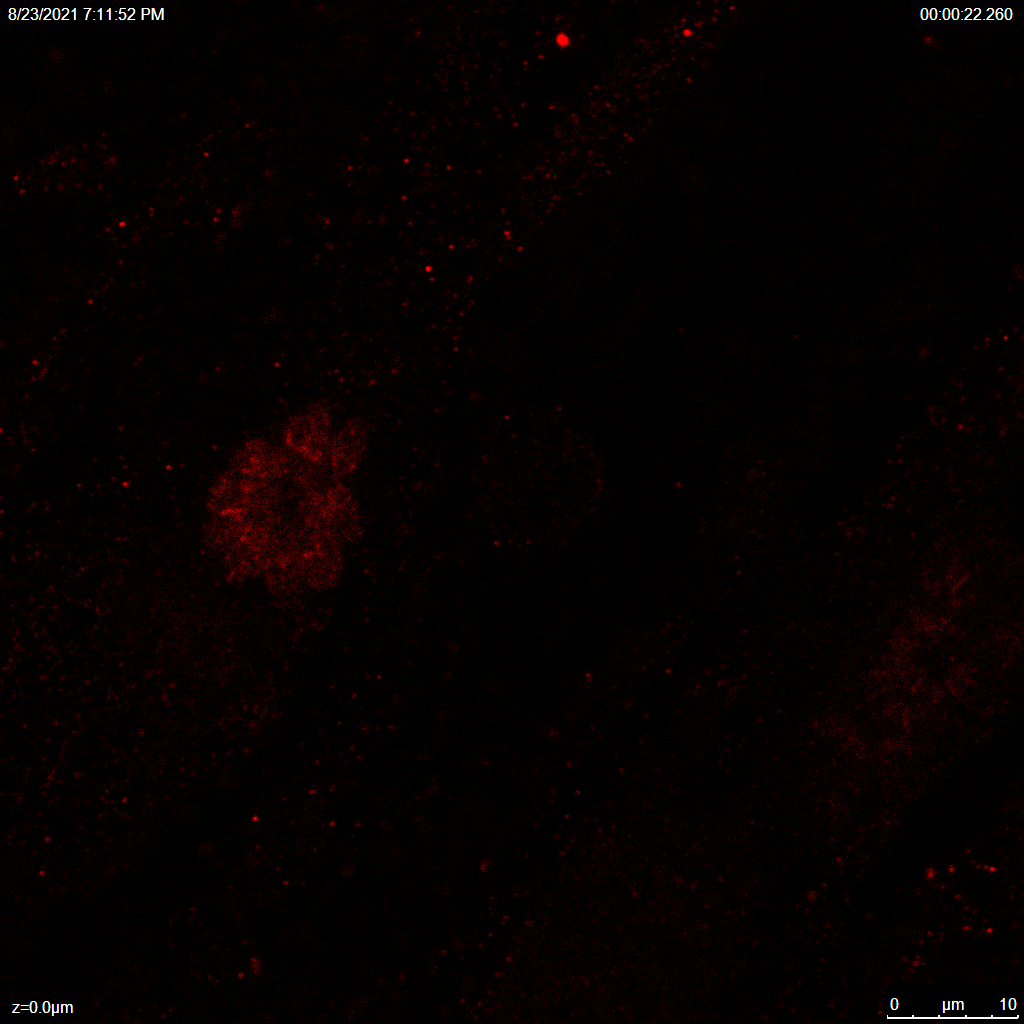

Supplement: Supplementary file 2 [file DataSheet1.ZIP › Figure 1/m12-1-red.png]

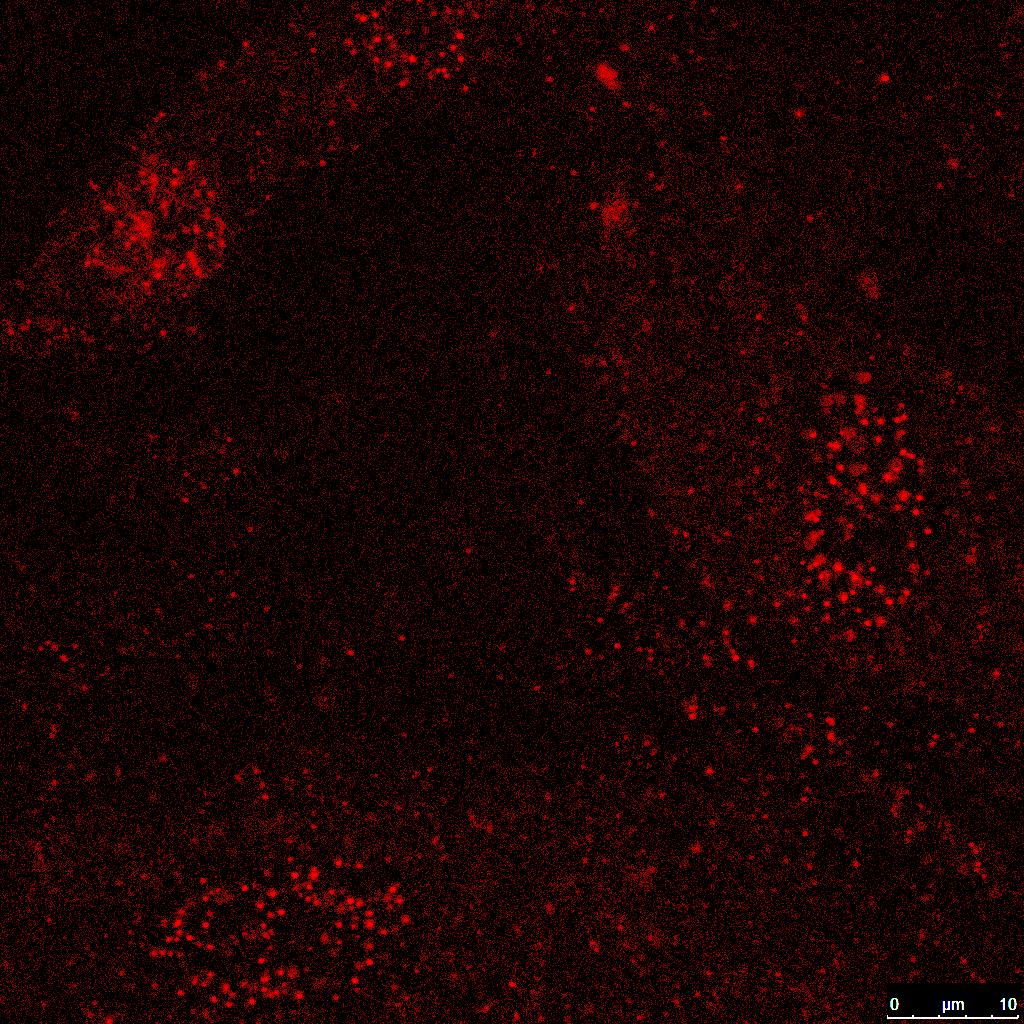

Supplement: Supplementary file 2 [file DataSheet1.ZIP › Figure 1/m12-ha.png]

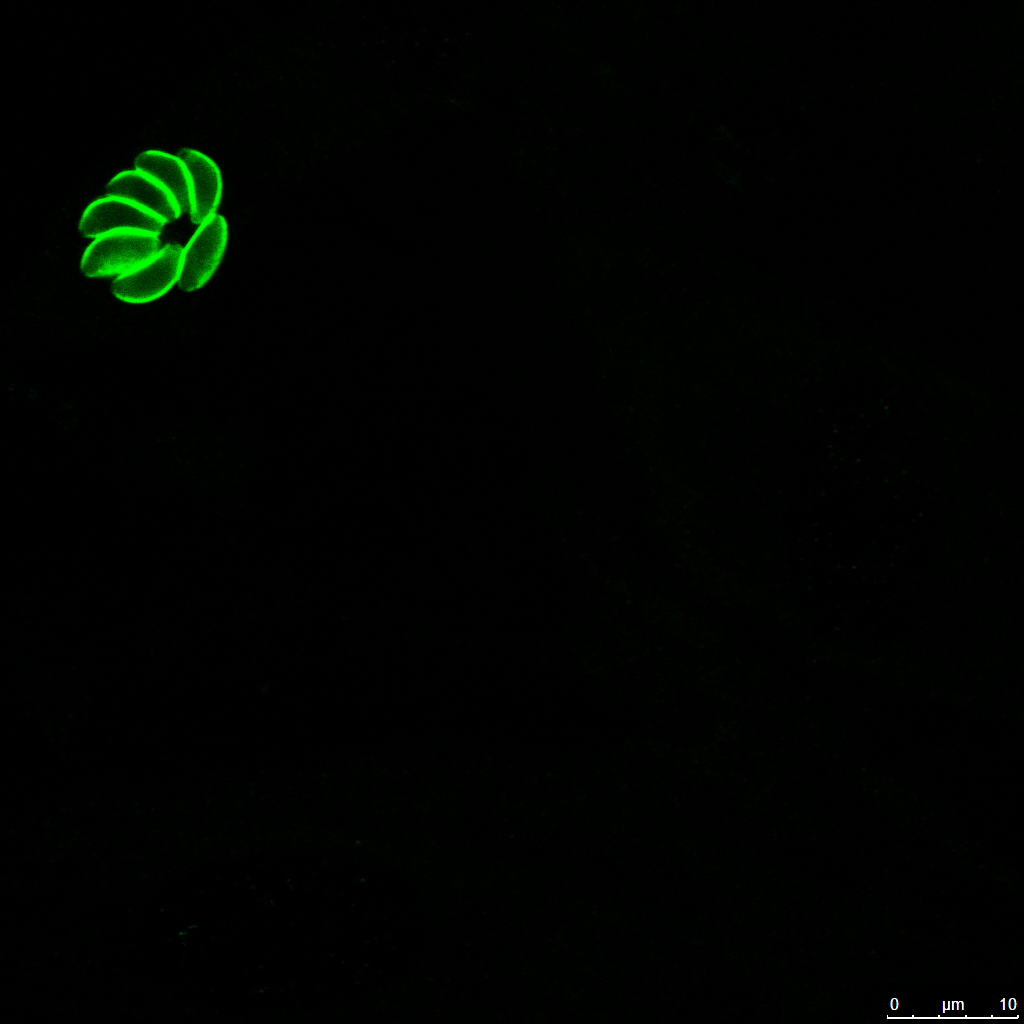

Supplement: Supplementary file 2 [file DataSheet1.ZIP › Figure 1/m12-imc.png]

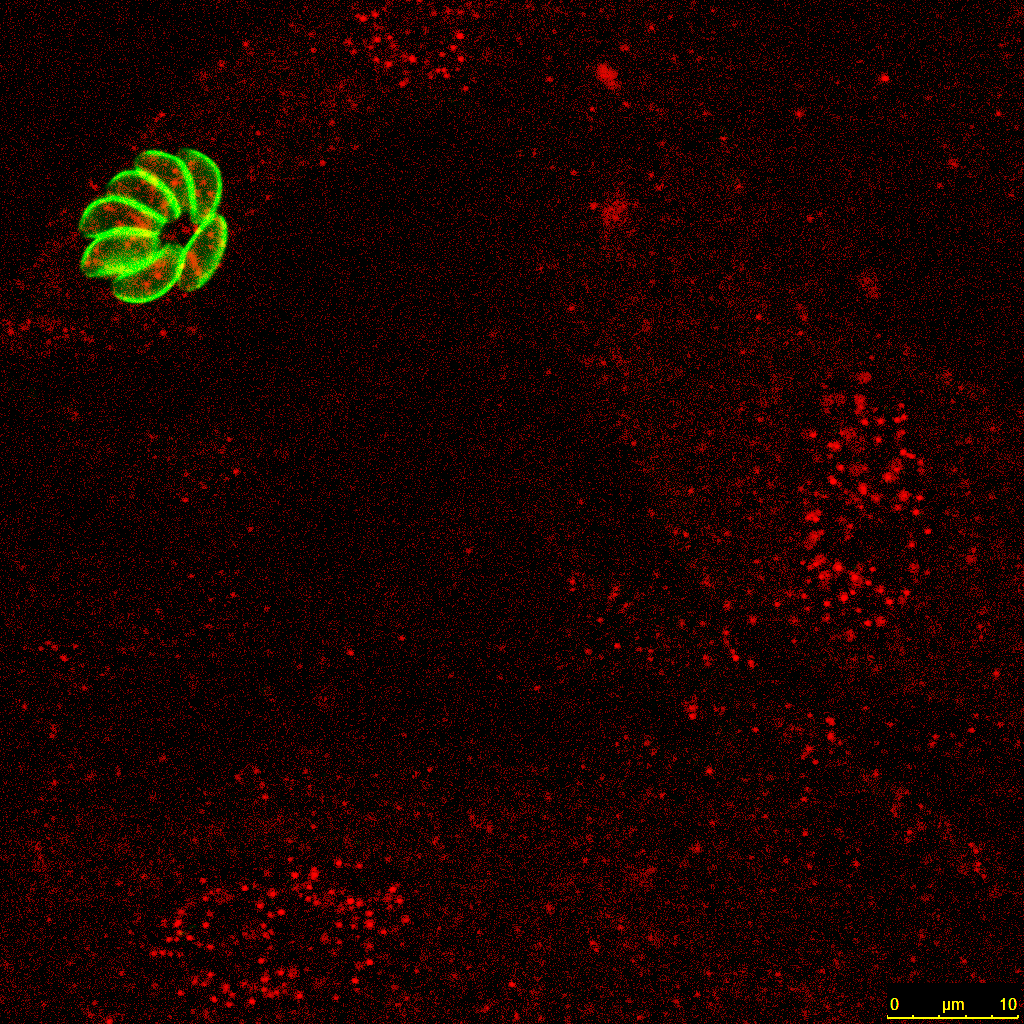

Supplement: Supplementary file 2 [file DataSheet1.ZIP › Figure 1/m12-merge.tif]

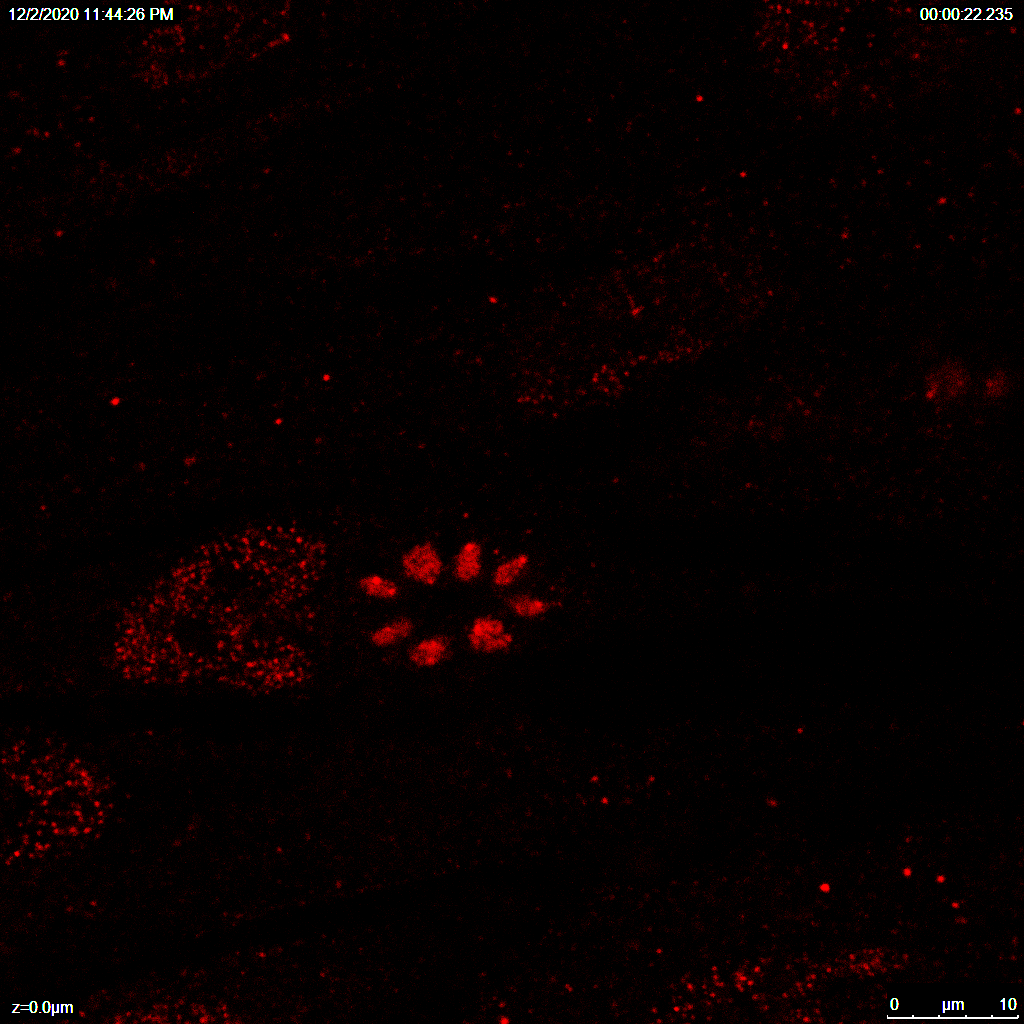

Supplement: Supplementary file 2 [file DataSheet1.ZIP › Figure 1/m4-ha.png]

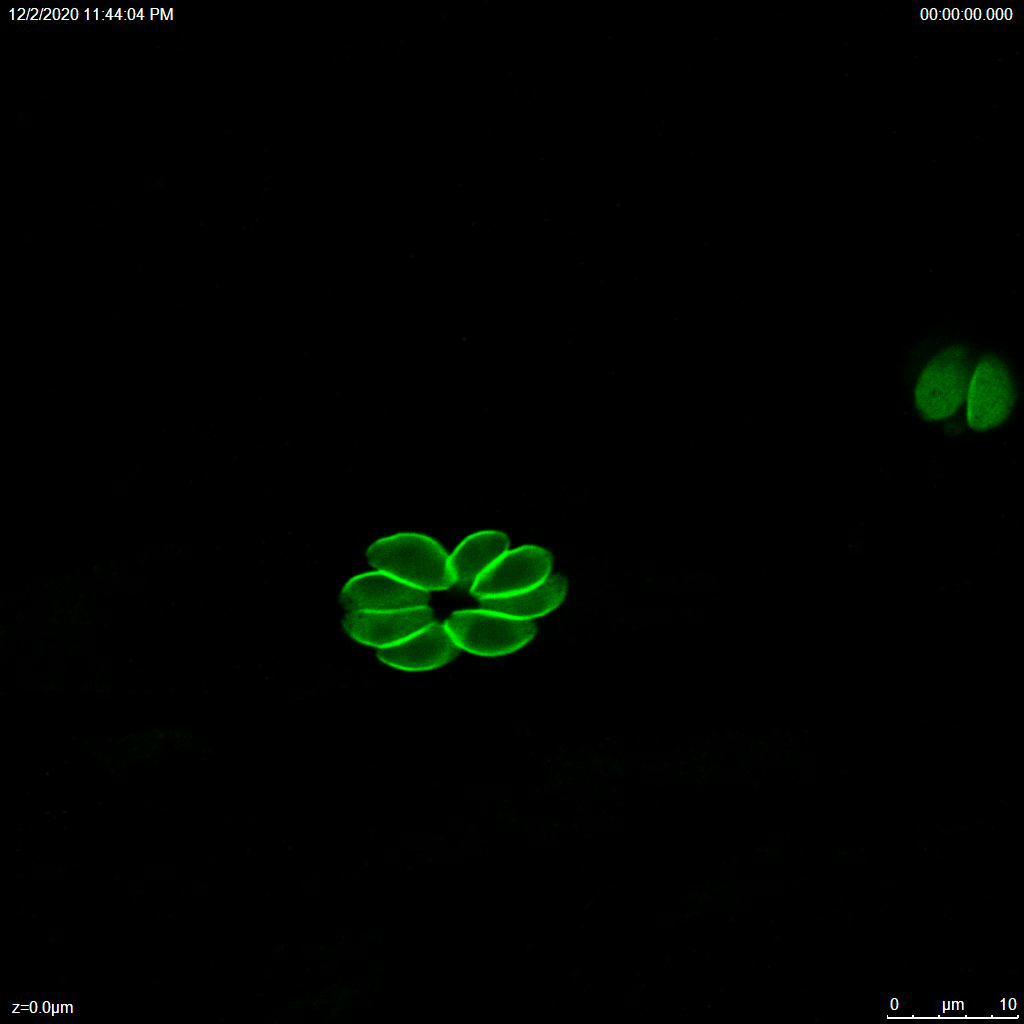

Supplement: Supplementary file 2 [file DataSheet1.ZIP › Figure 1/m4-imc.png]

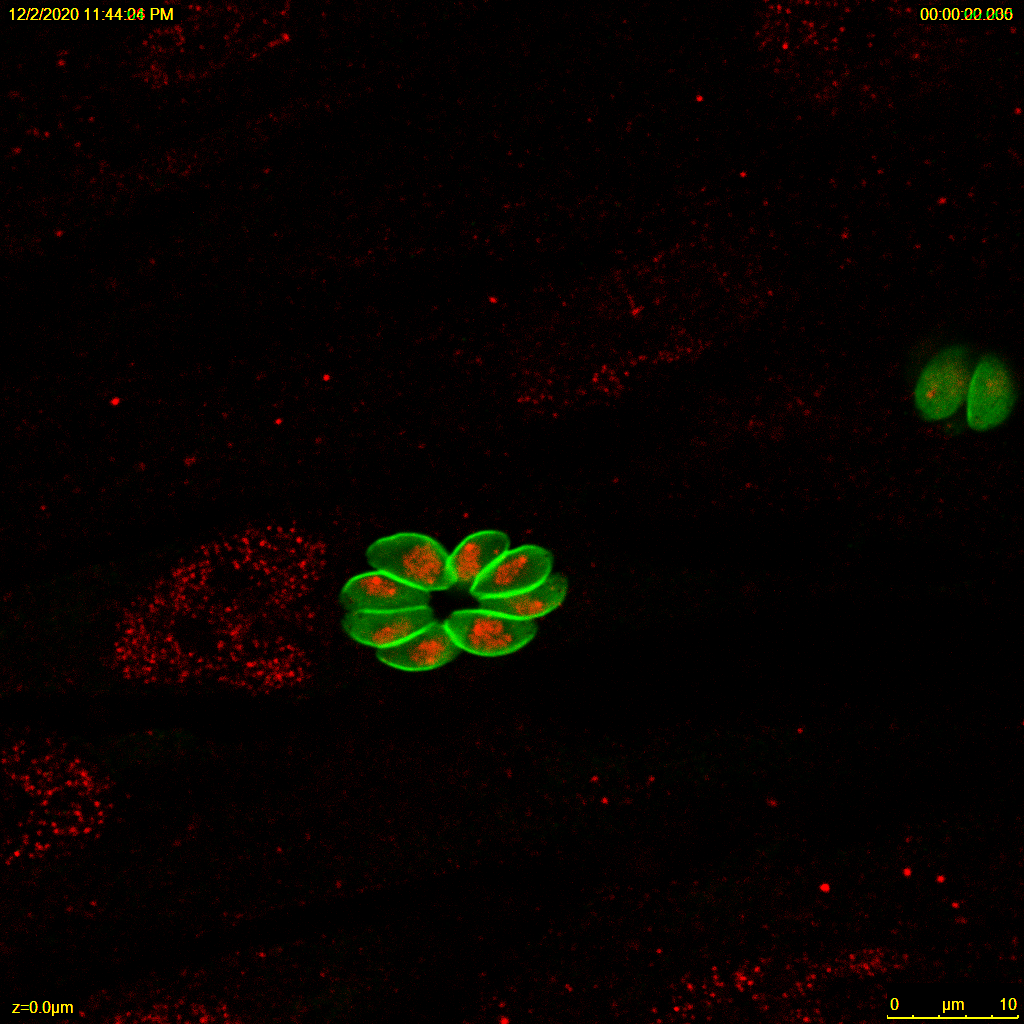

Supplement: Supplementary file 2 [file DataSheet1.ZIP › Figure 1/m4-merge.tif]

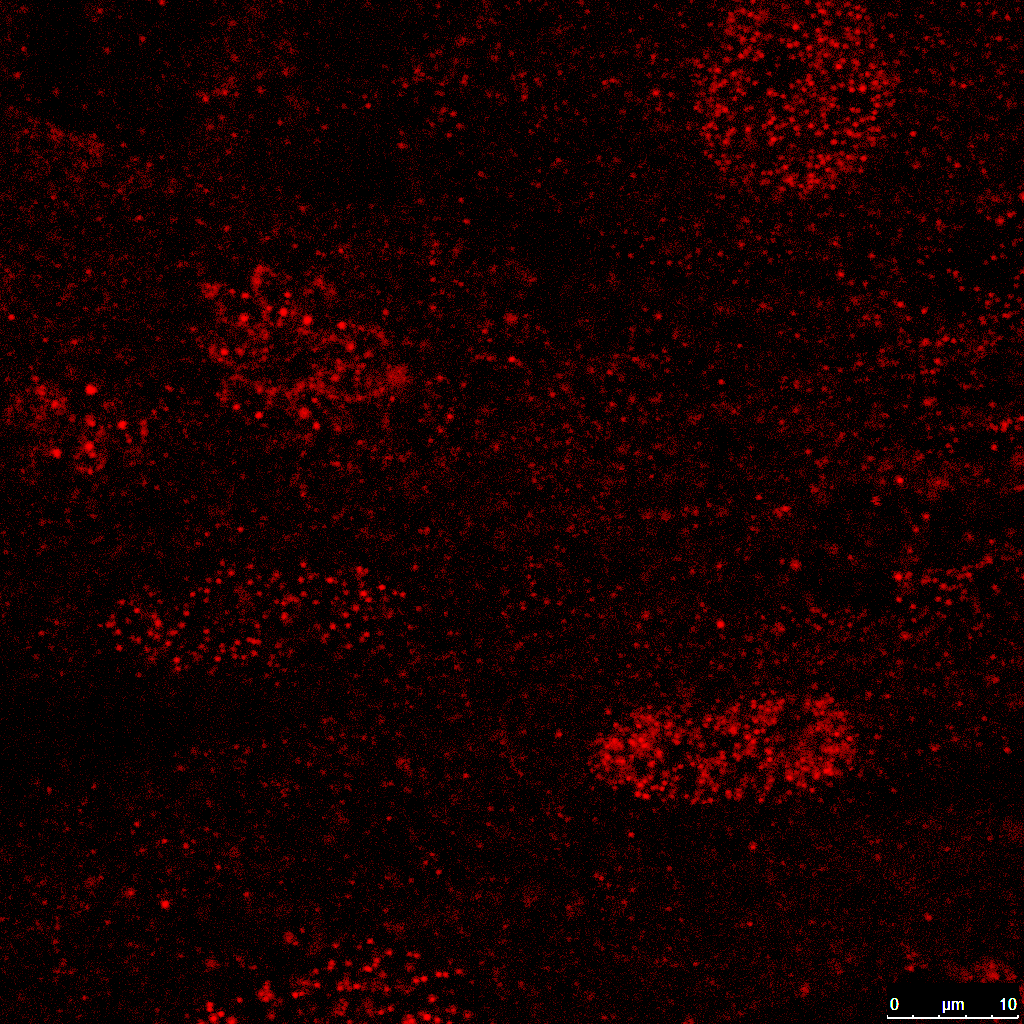

Supplement: Supplementary file 2 [file DataSheet1.ZIP › Figure 1/m8-ha.png]

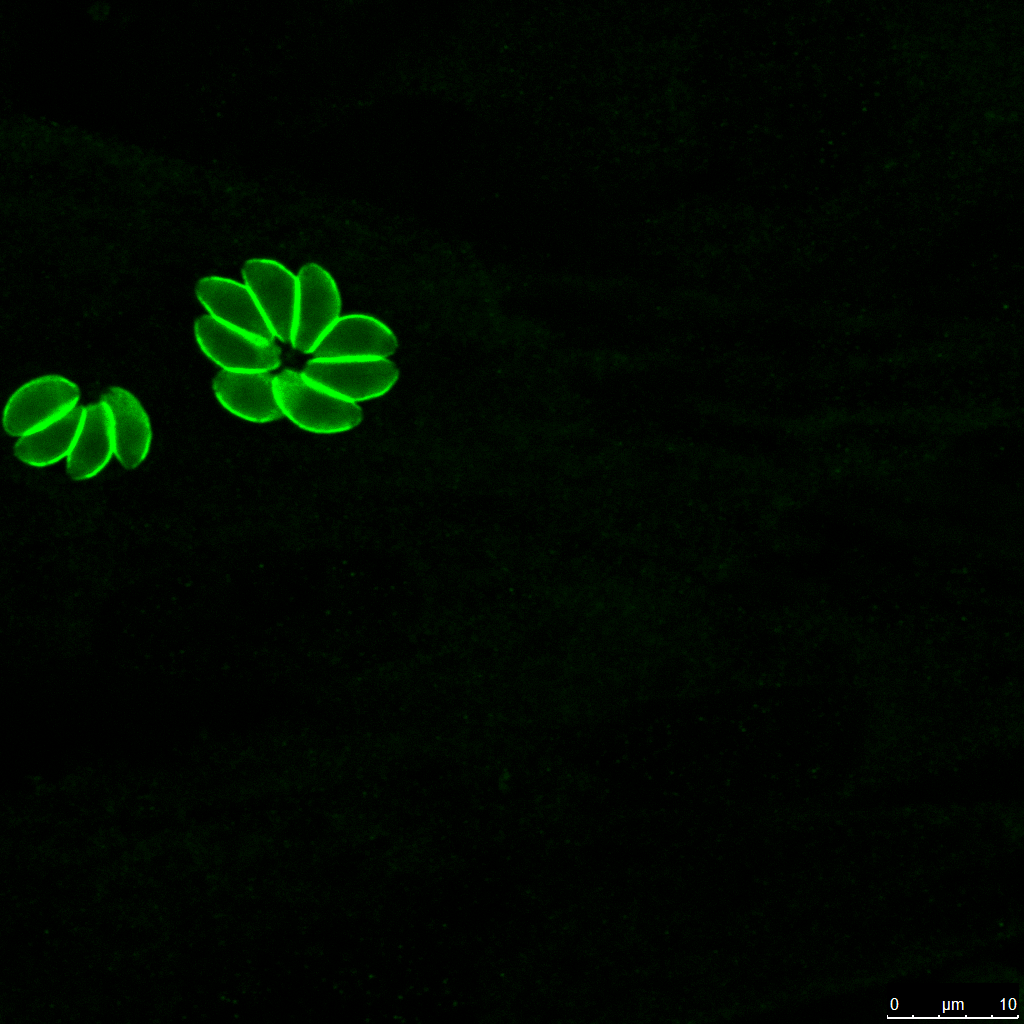

Supplement: Supplementary file 2 [file DataSheet1.ZIP › Figure 1/m8-imc.png]

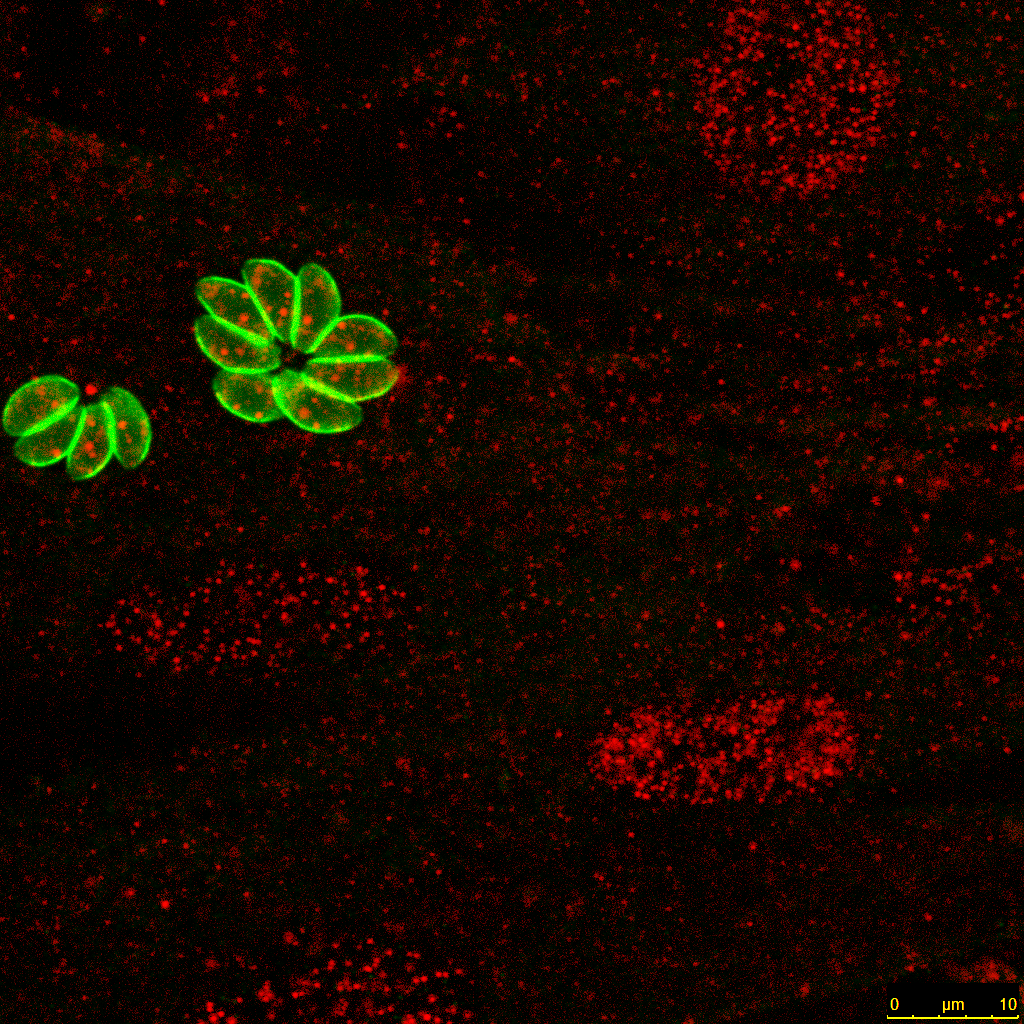

Supplement: Supplementary file 2 [file DataSheet1.ZIP › Figure 1/m8-merge.tif]

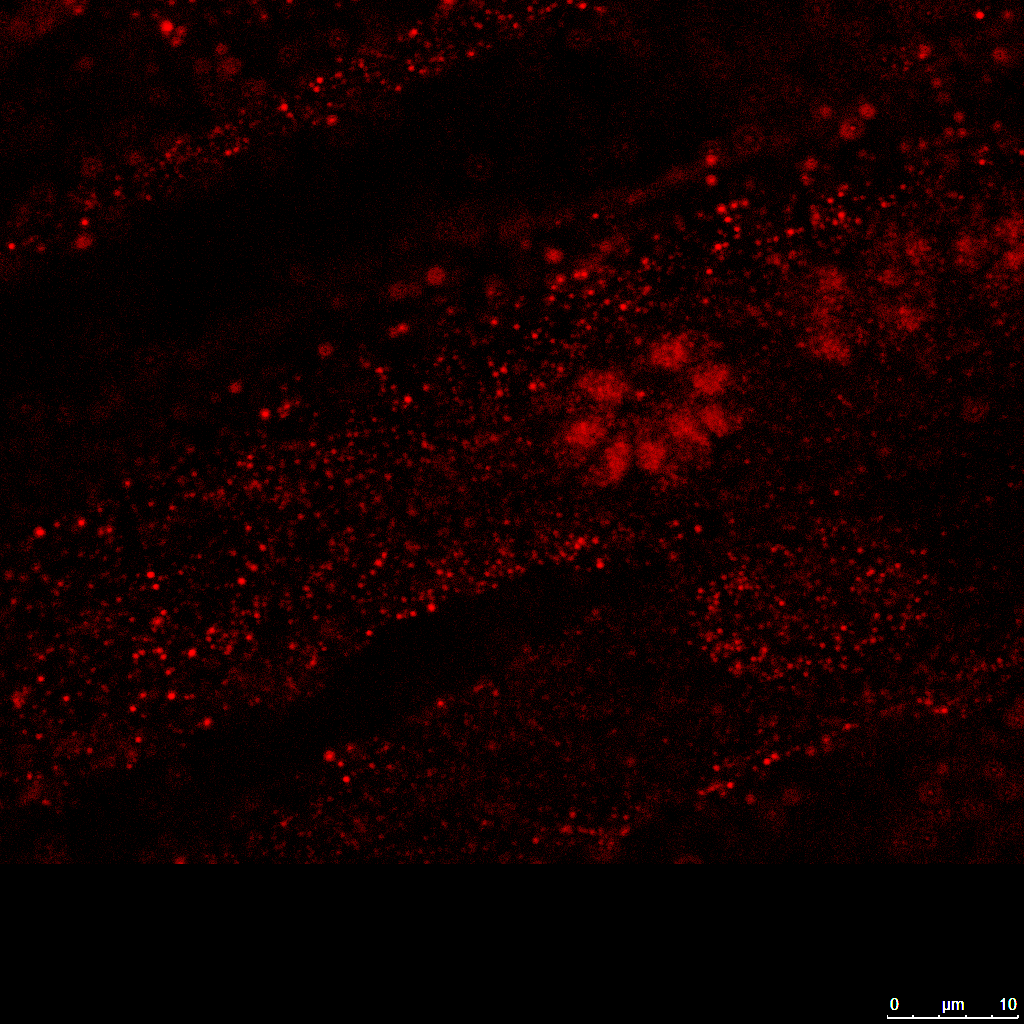

Supplement: Supplementary file 2 [file DataSheet1.ZIP › Figure 1/m9-ha.png]

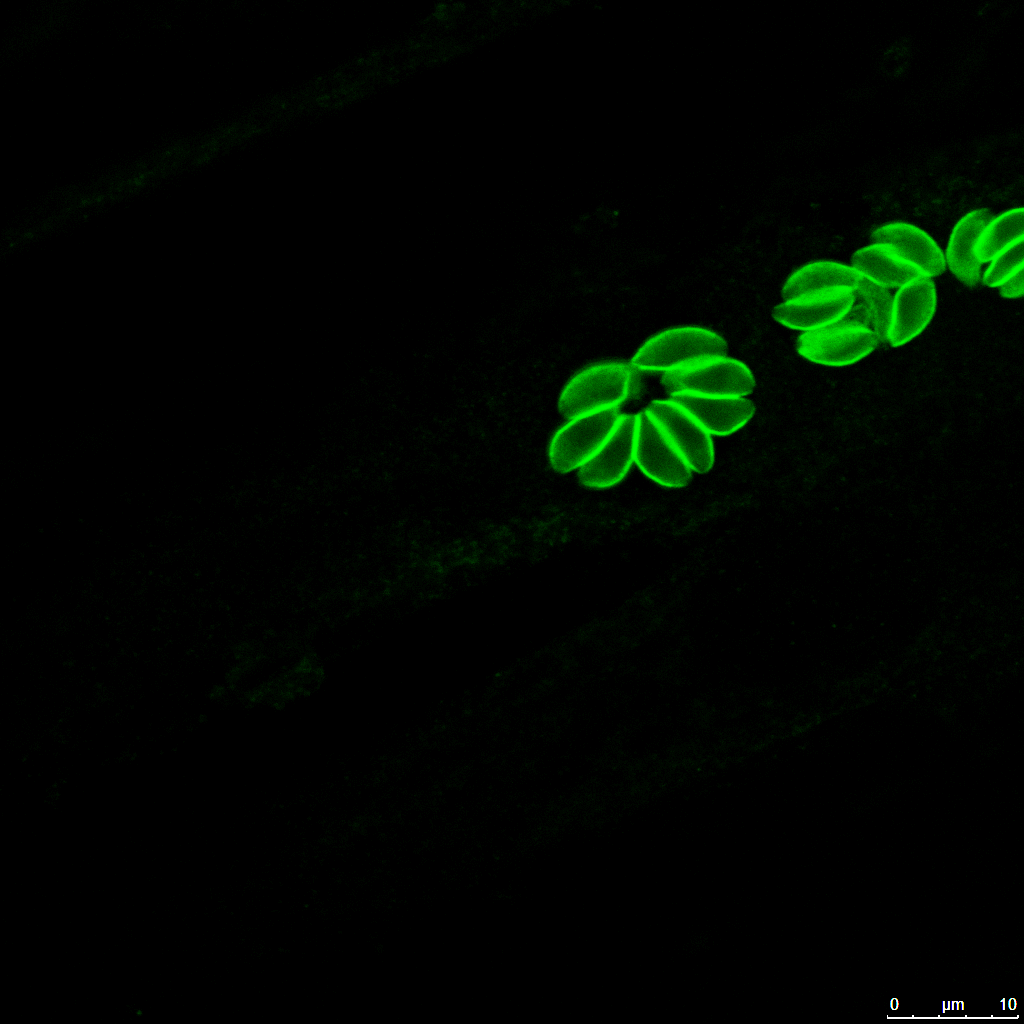

Supplement: Supplementary file 2 [file DataSheet1.ZIP › Figure 1/m9-imc.png]

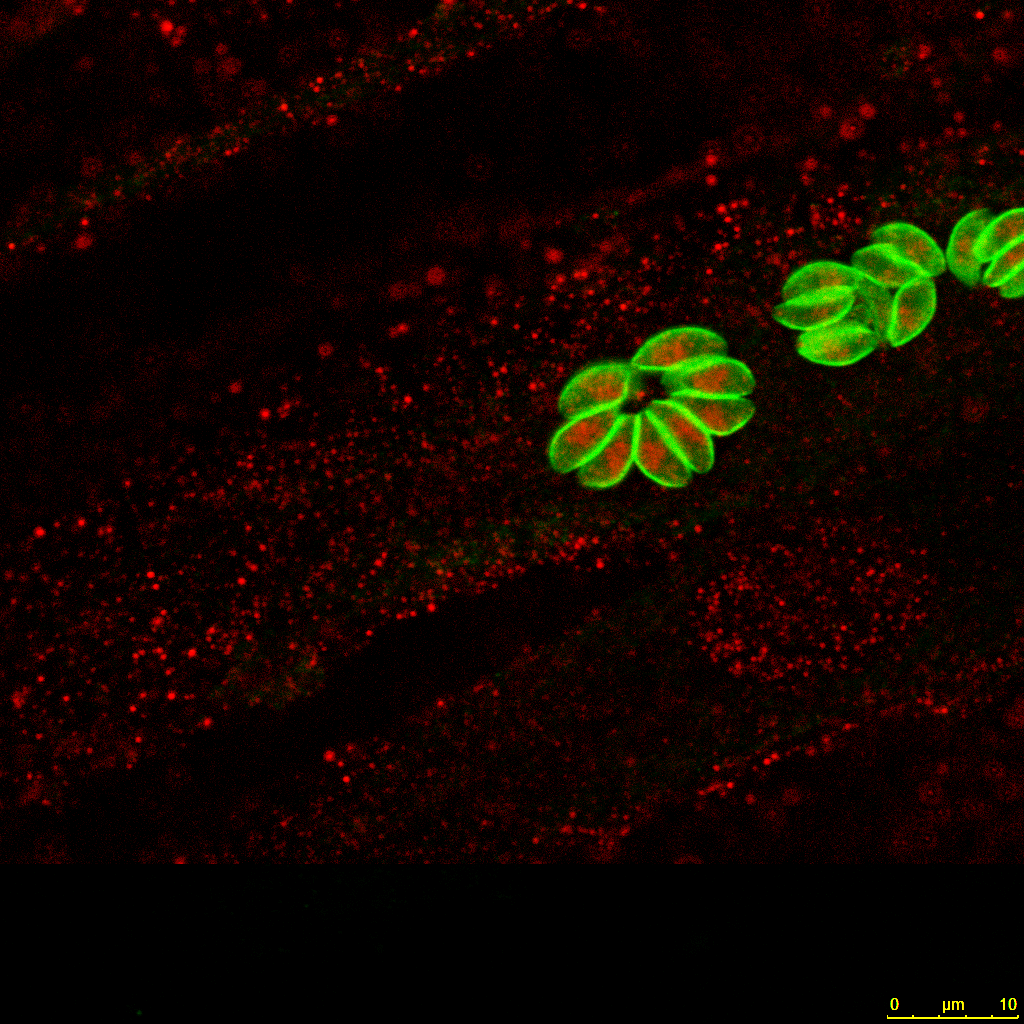

Supplement: Supplementary file 2 [file DataSheet1.ZIP › Figure 1/m9-merge.tif]

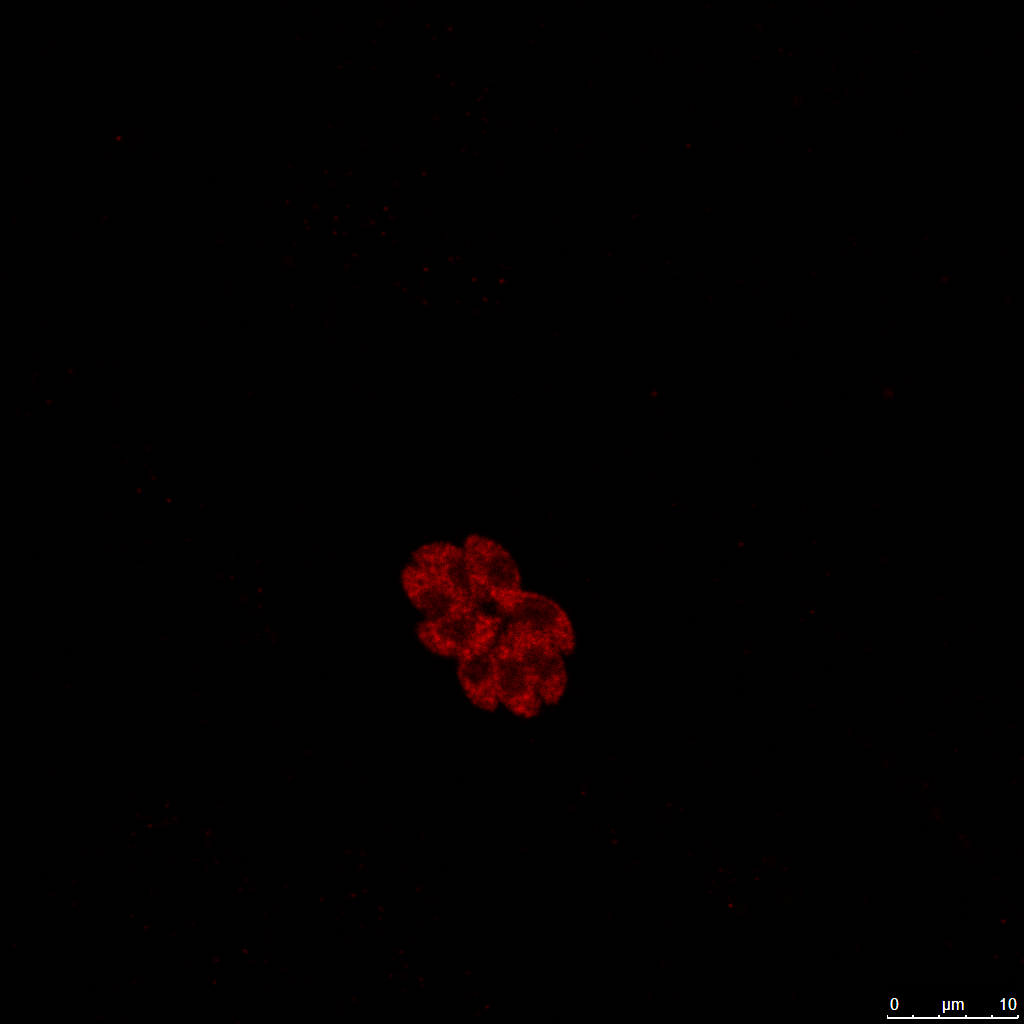

Supplement: Supplementary file 2 [file DataSheet1.ZIP › Figure 1/pp5-ha.png]

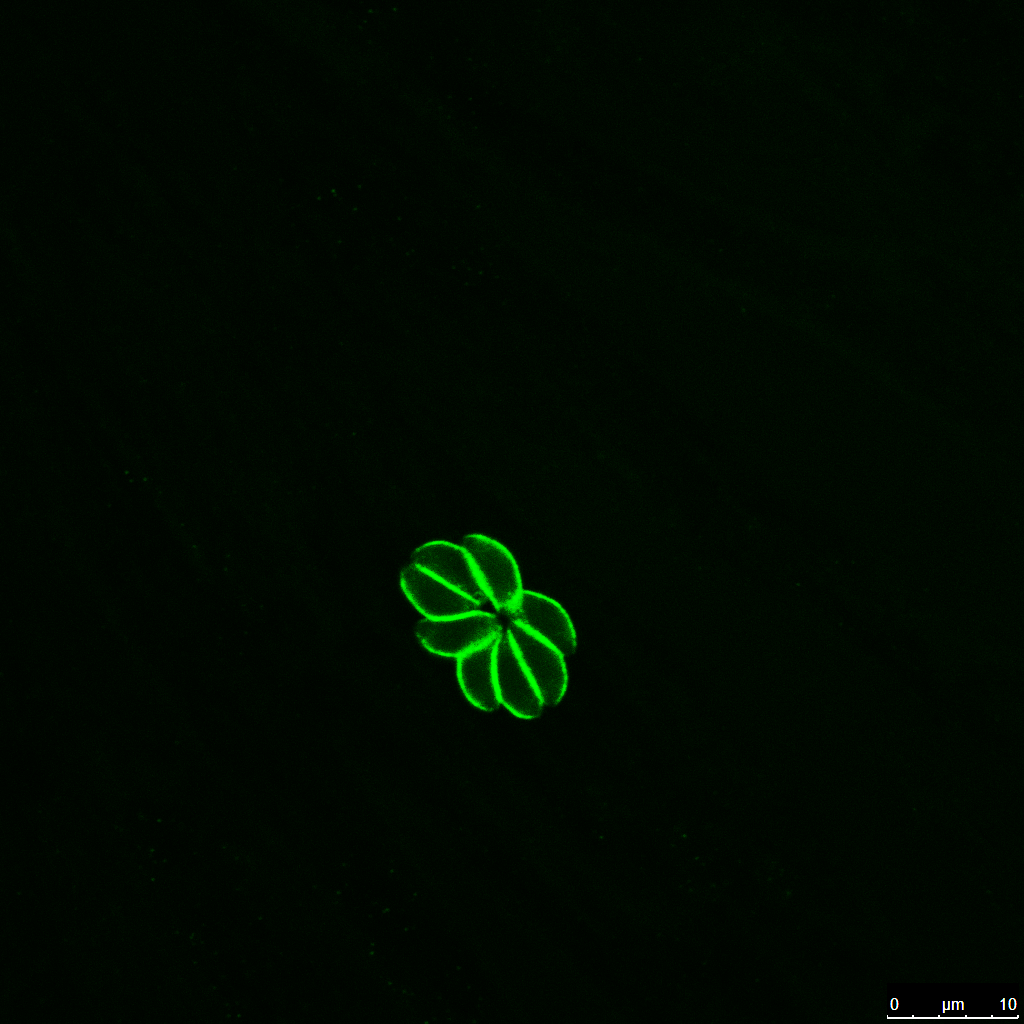

Supplement: Supplementary file 2 [file DataSheet1.ZIP › Figure 1/pp5-imc.png]

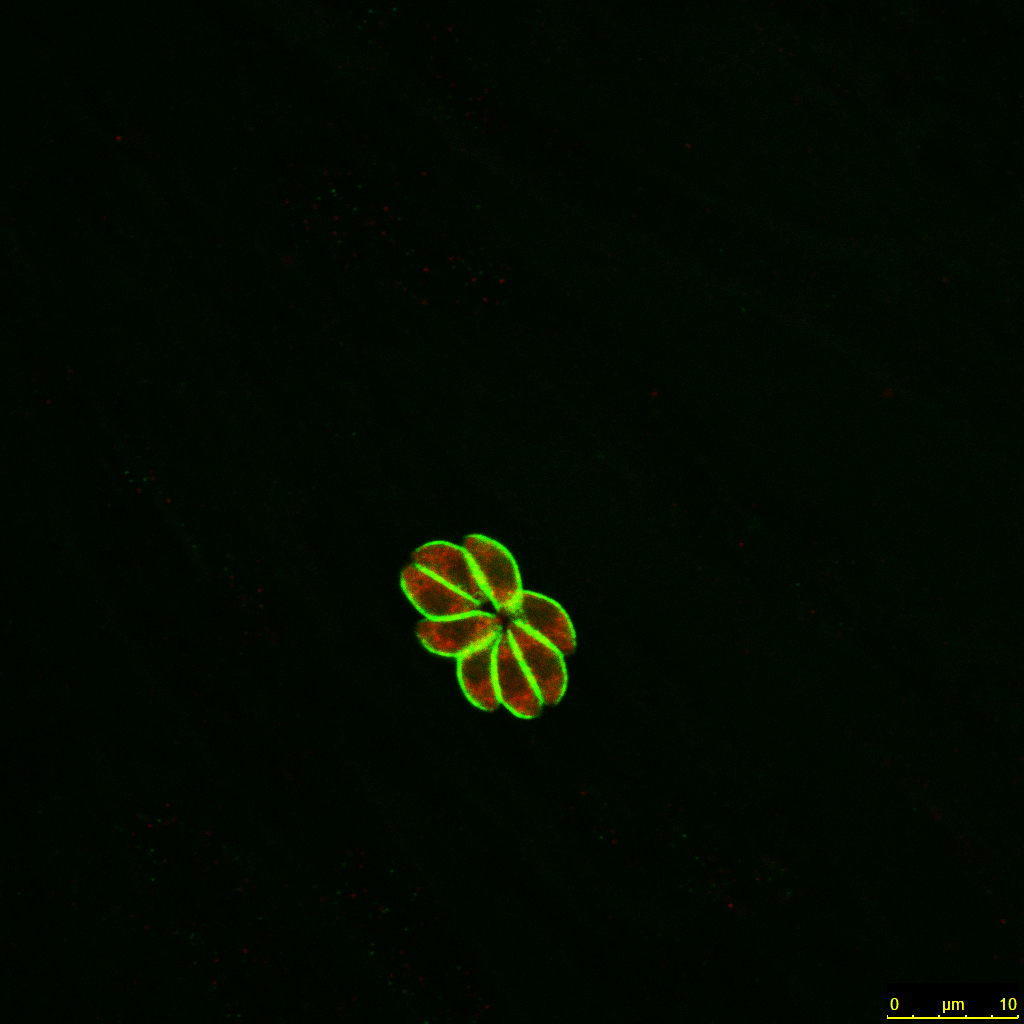

Supplement: Supplementary file 2 [file DataSheet1.ZIP › Figure 1/pp5-merge.tif]

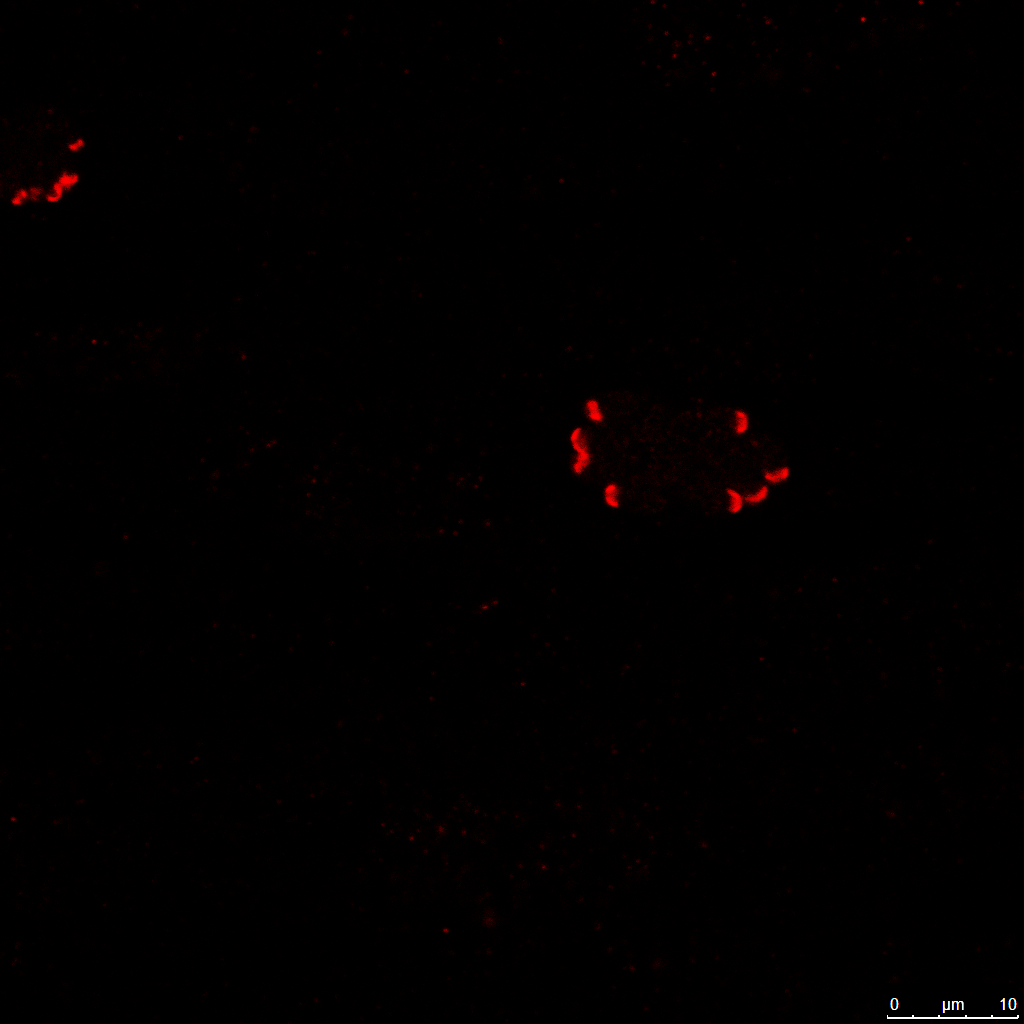

Supplement: Supplementary file 2 [file DataSheet1.ZIP › Figure 1/pp7-ha.png]

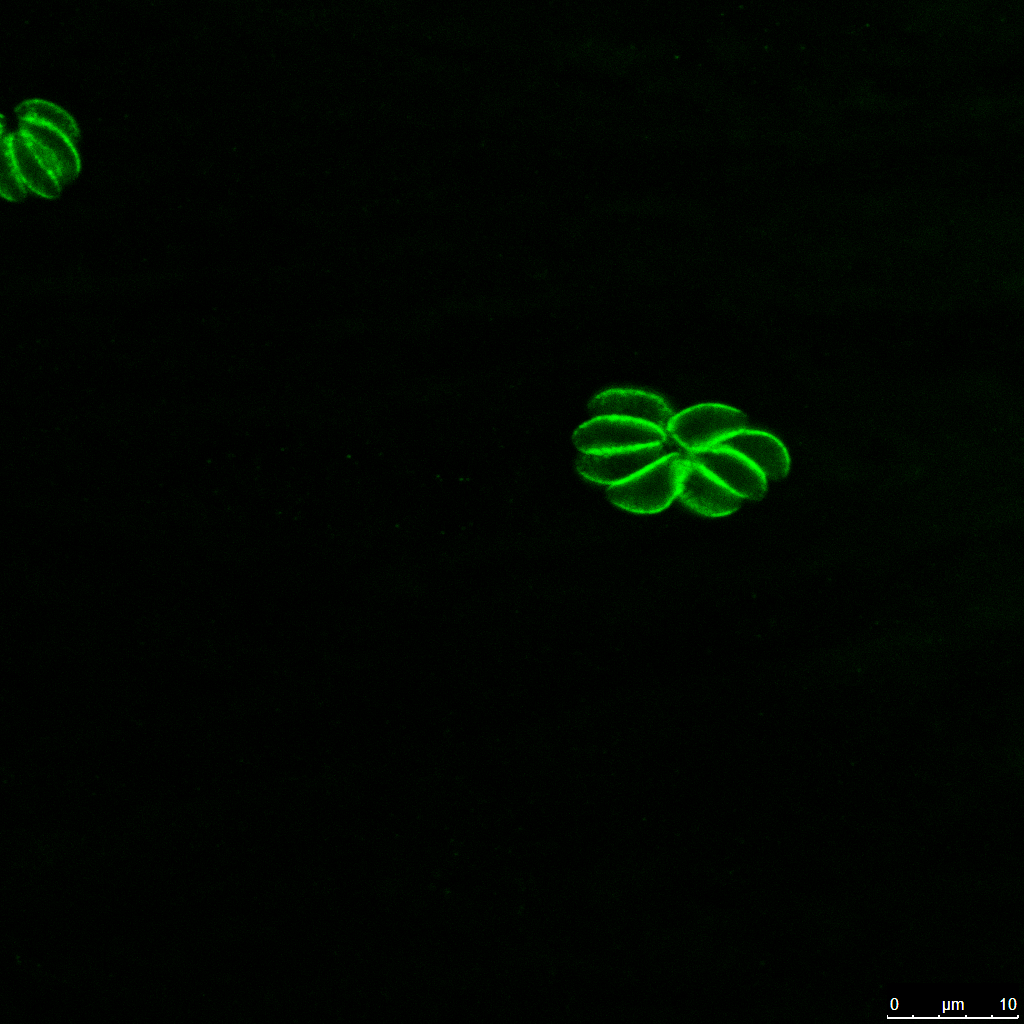

Supplement: Supplementary file 2 [file DataSheet1.ZIP › Figure 1/pp7-imc.png]

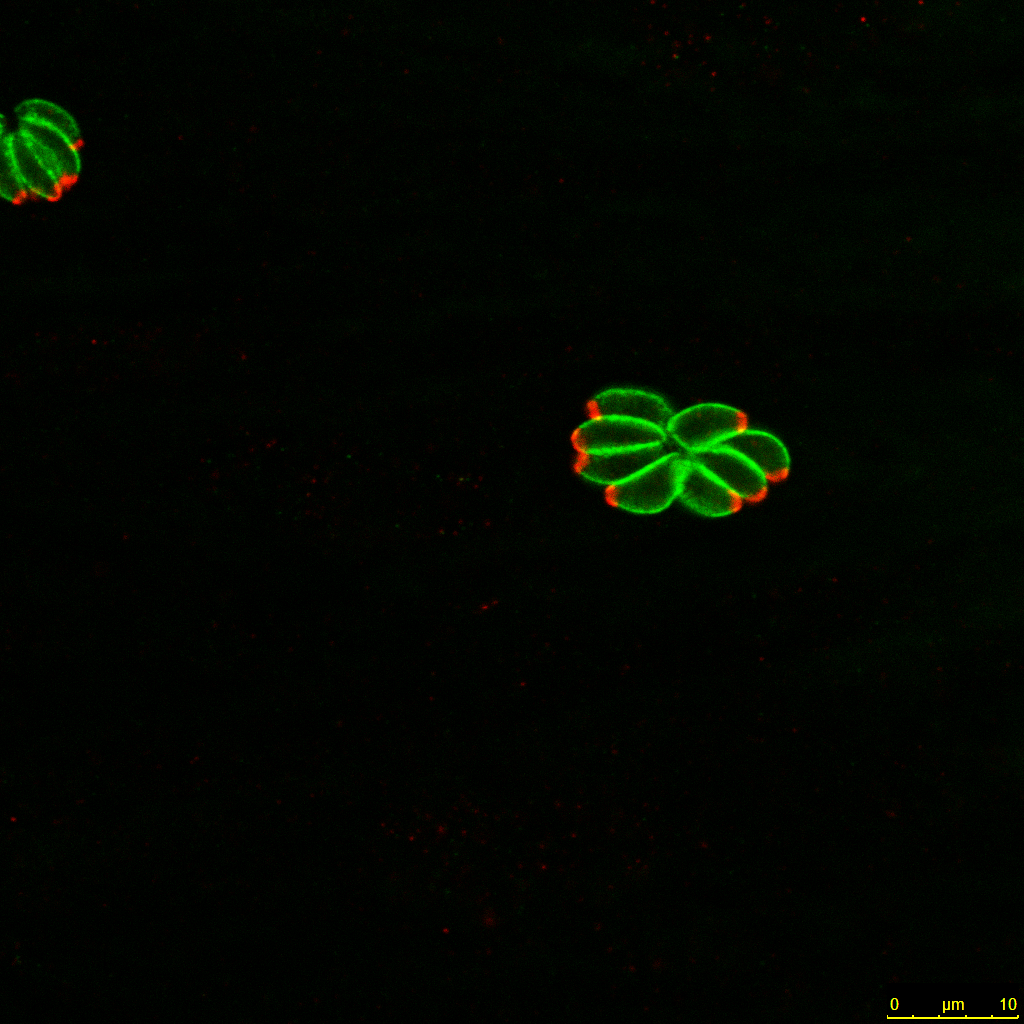

Supplement: Supplementary file 2 [file DataSheet1.ZIP › Figure 1/pp7-merge.tif]

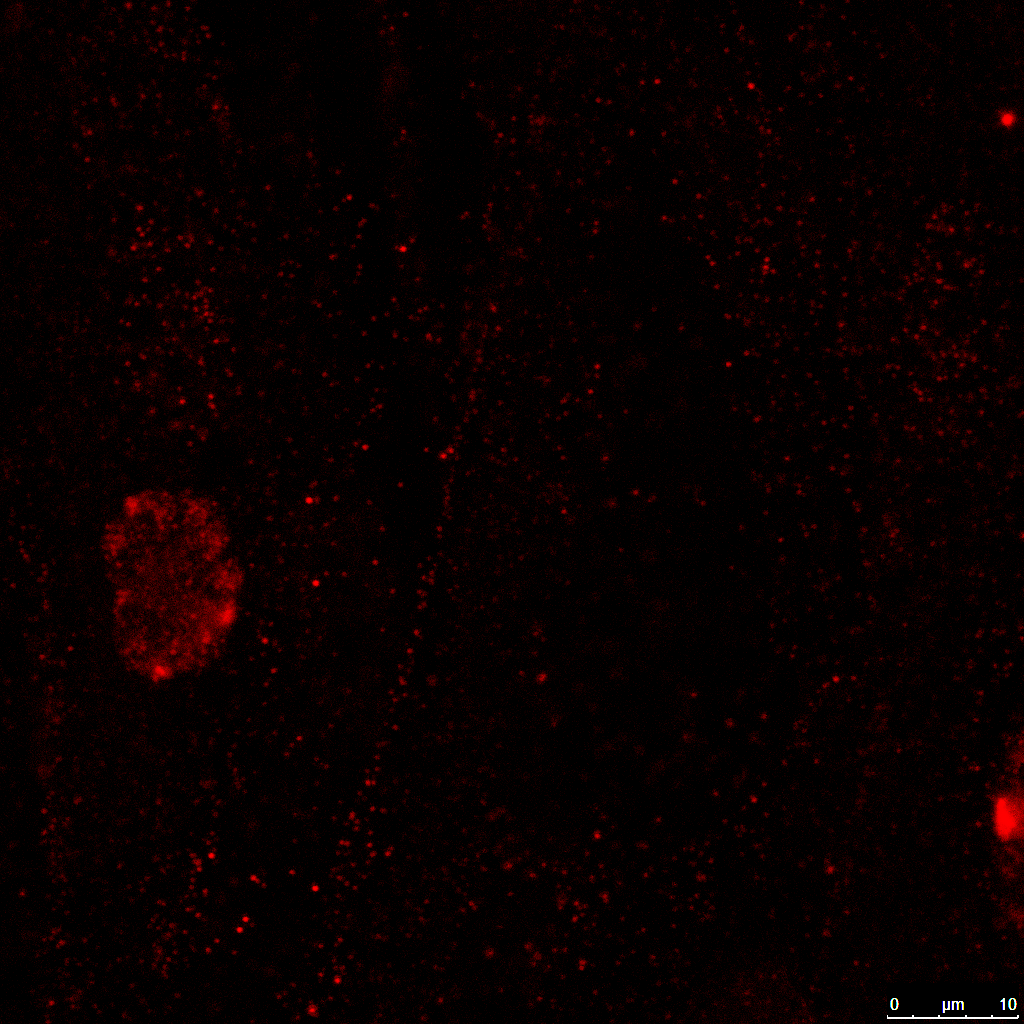

Supplement: Supplementary file 2 [file DataSheet1.ZIP › Figure 1/slp-ha.png]

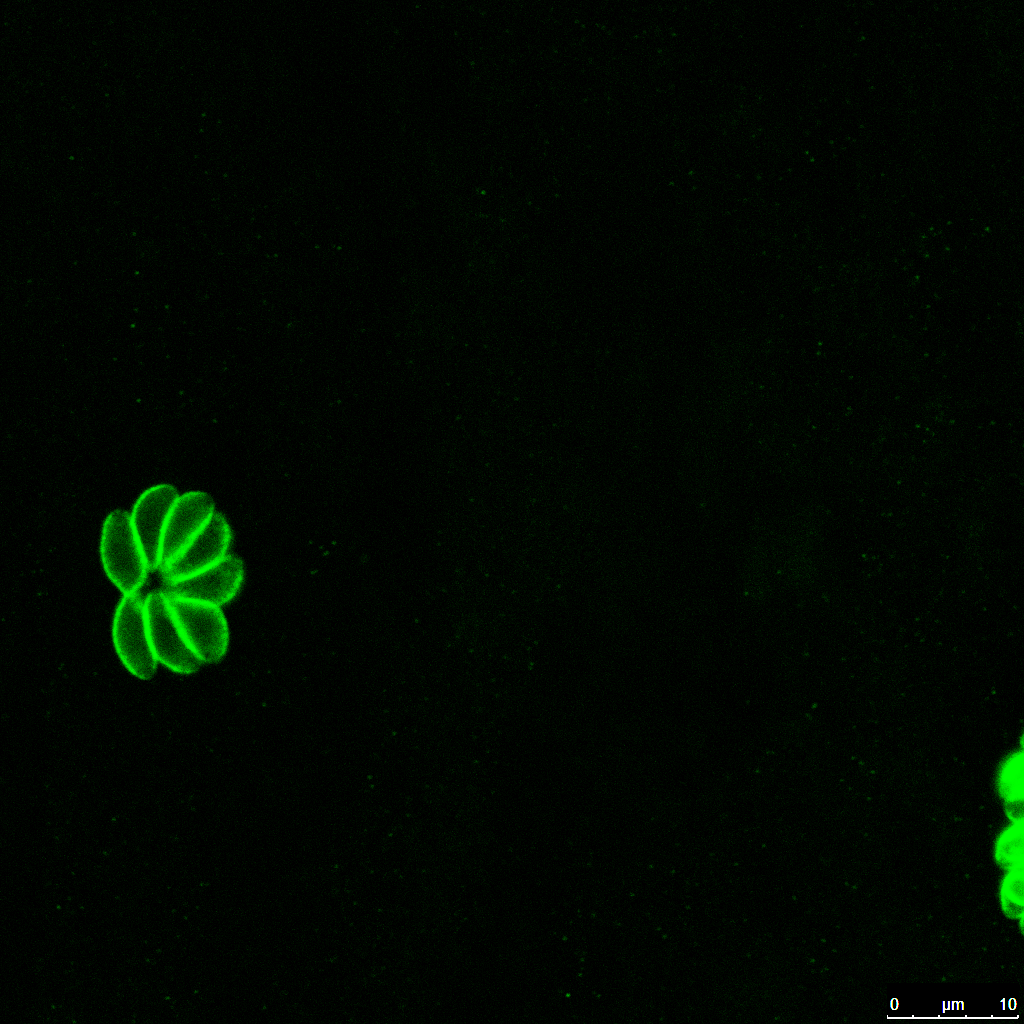

Supplement: Supplementary file 2 [file DataSheet1.ZIP › Figure 1/slp-imc.png]

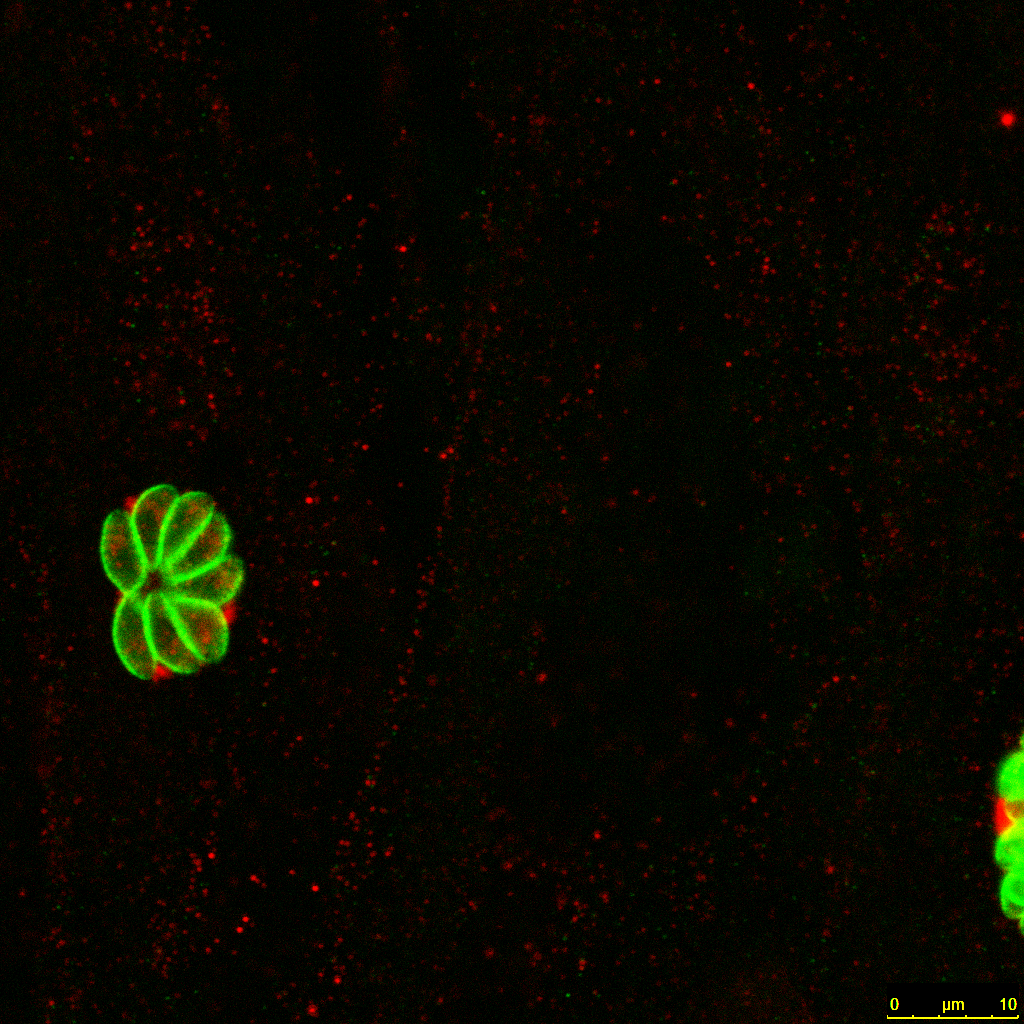

Supplement: Supplementary file 2 [file DataSheet1.ZIP › Figure 1/slp-merge.tif]

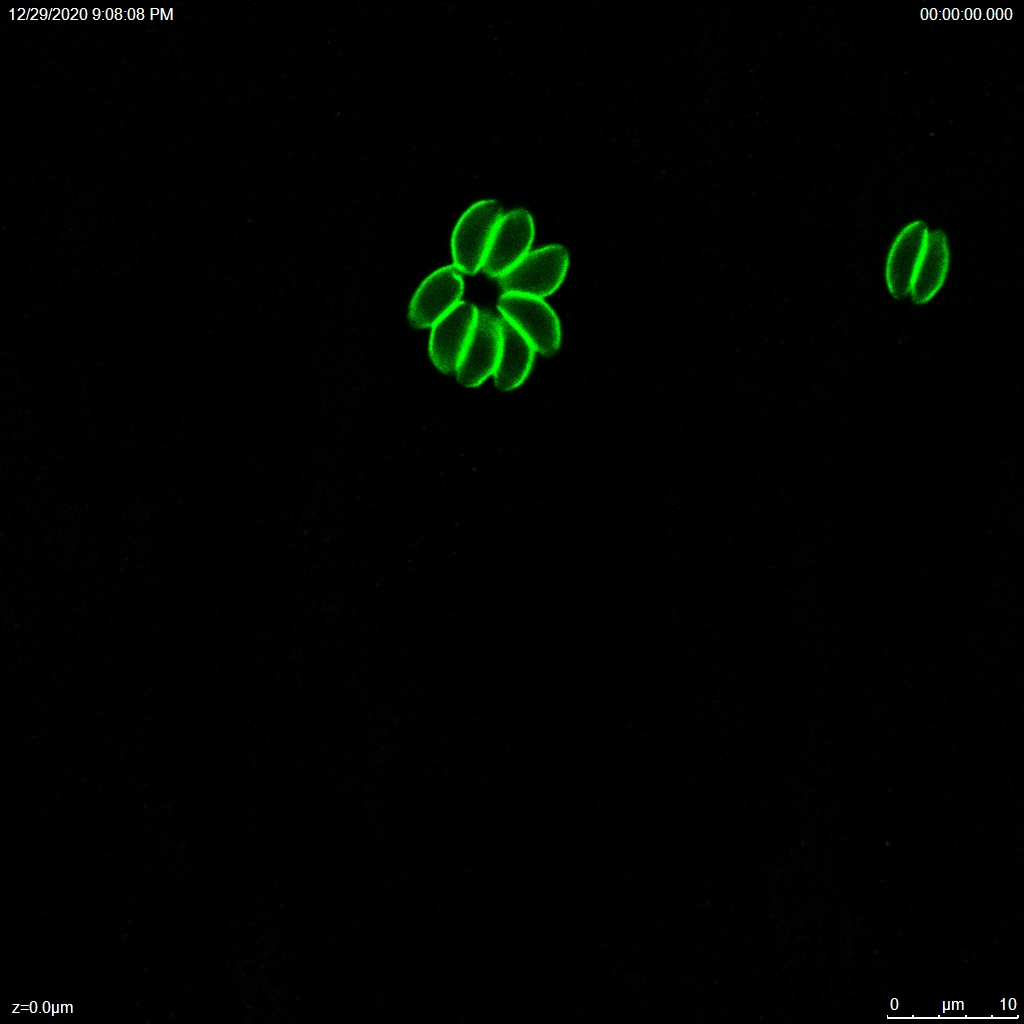

Supplement: Supplementary file 2 [file DataSheet1.ZIP › Figure 1/WT-green.png]

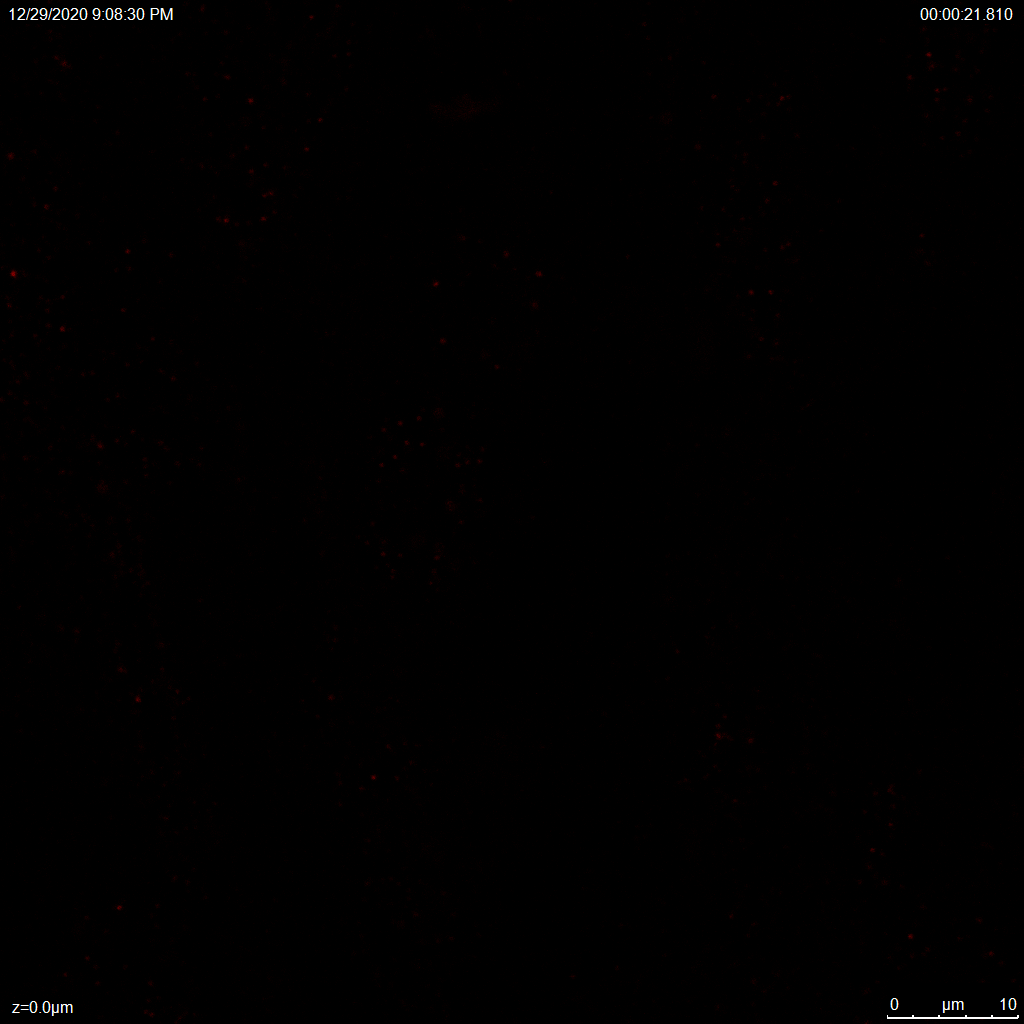

Supplement: Supplementary file 2 [file DataSheet1.ZIP › Figure 1/WT-red.png]

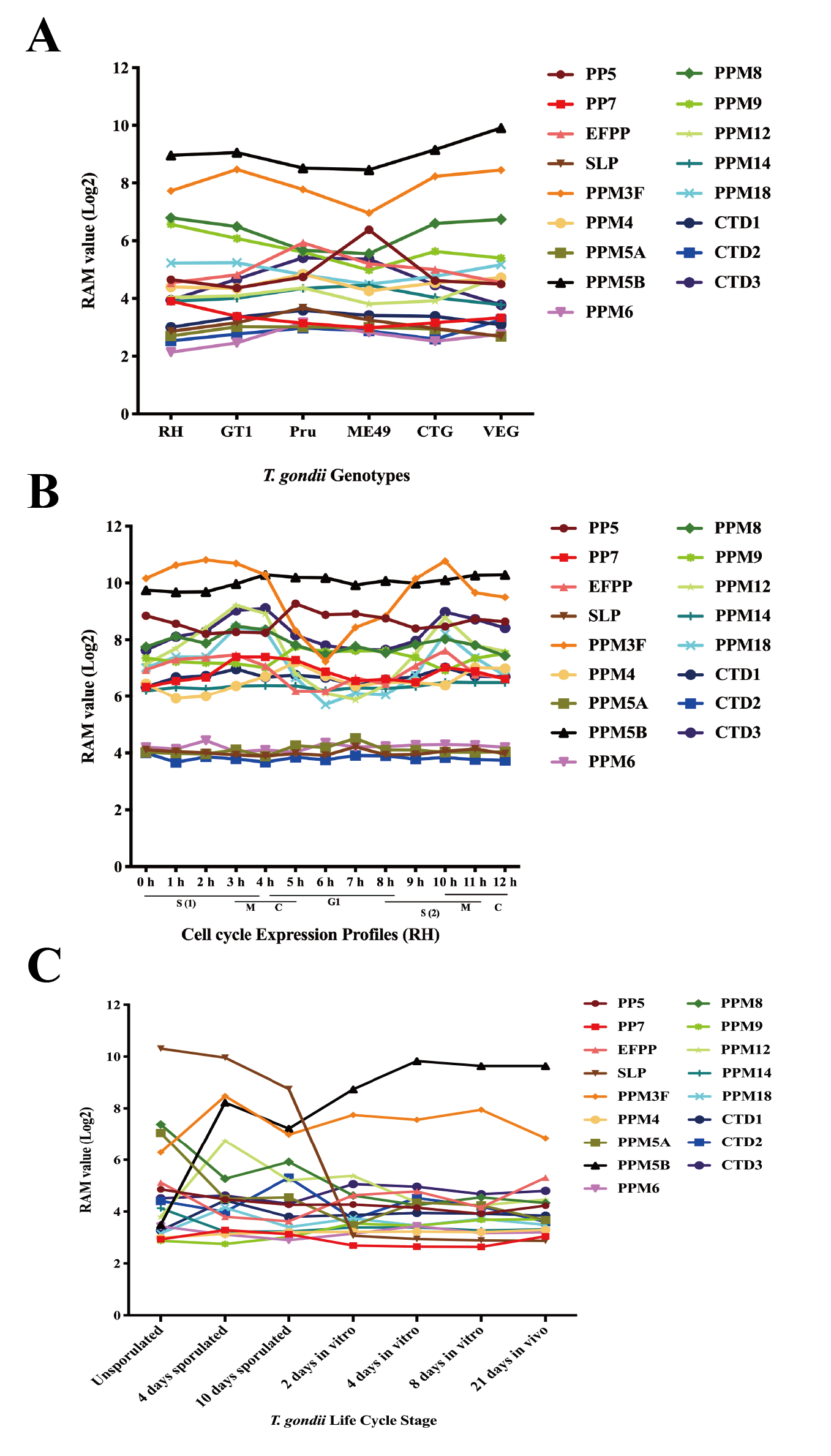

Supplement: Supplementary file 3 [file Image1.TIF]

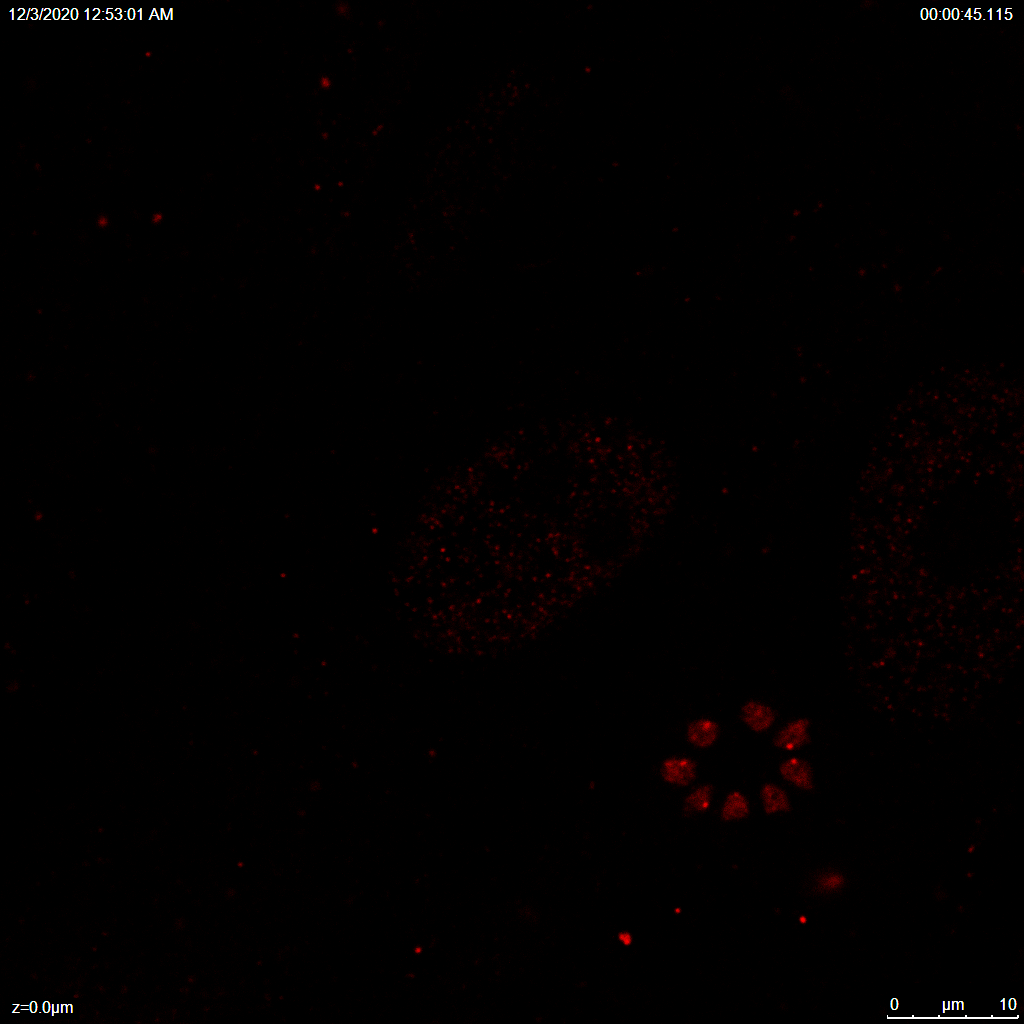

Supplement: Supplementary file 6 [file DataSheet2.ZIP › Figure 2/m4-ha.png]

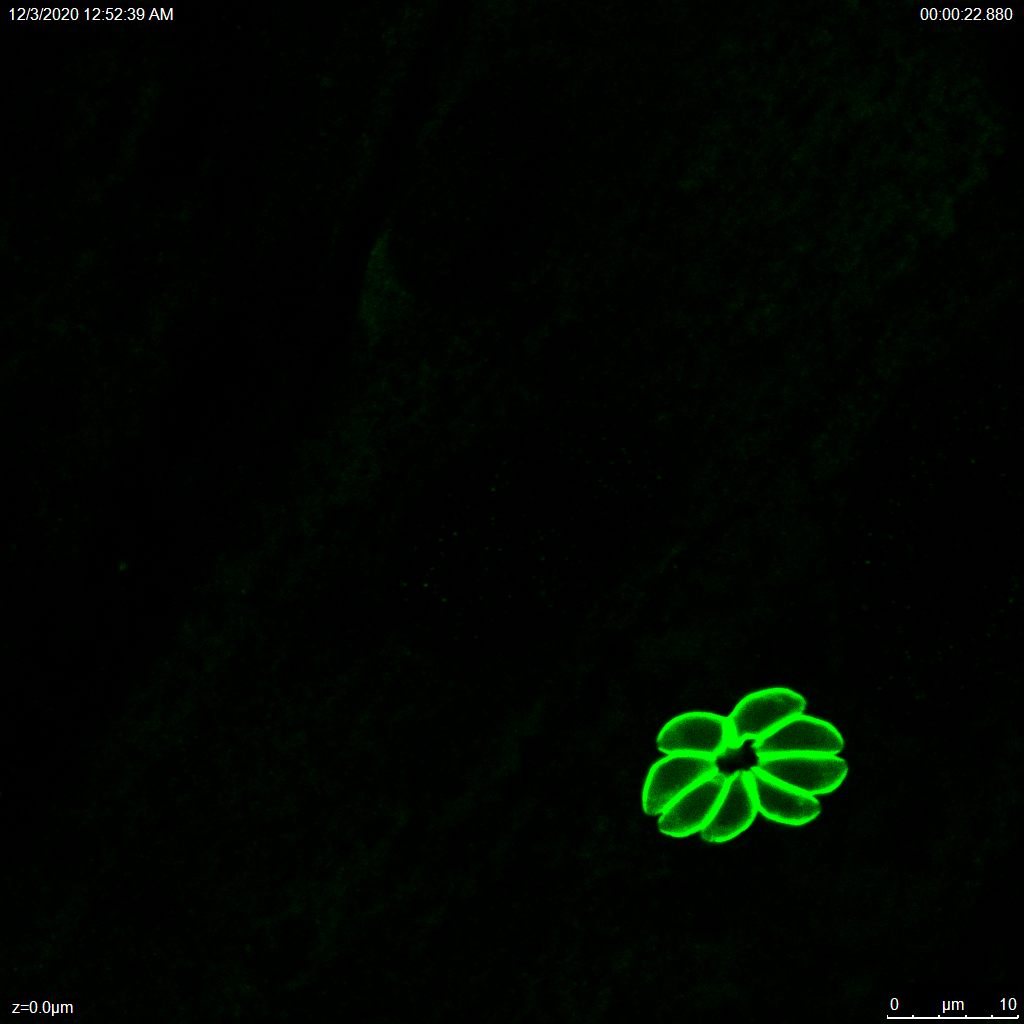

Supplement: Supplementary file 6 [file DataSheet2.ZIP › Figure 2/m4-imc.png]

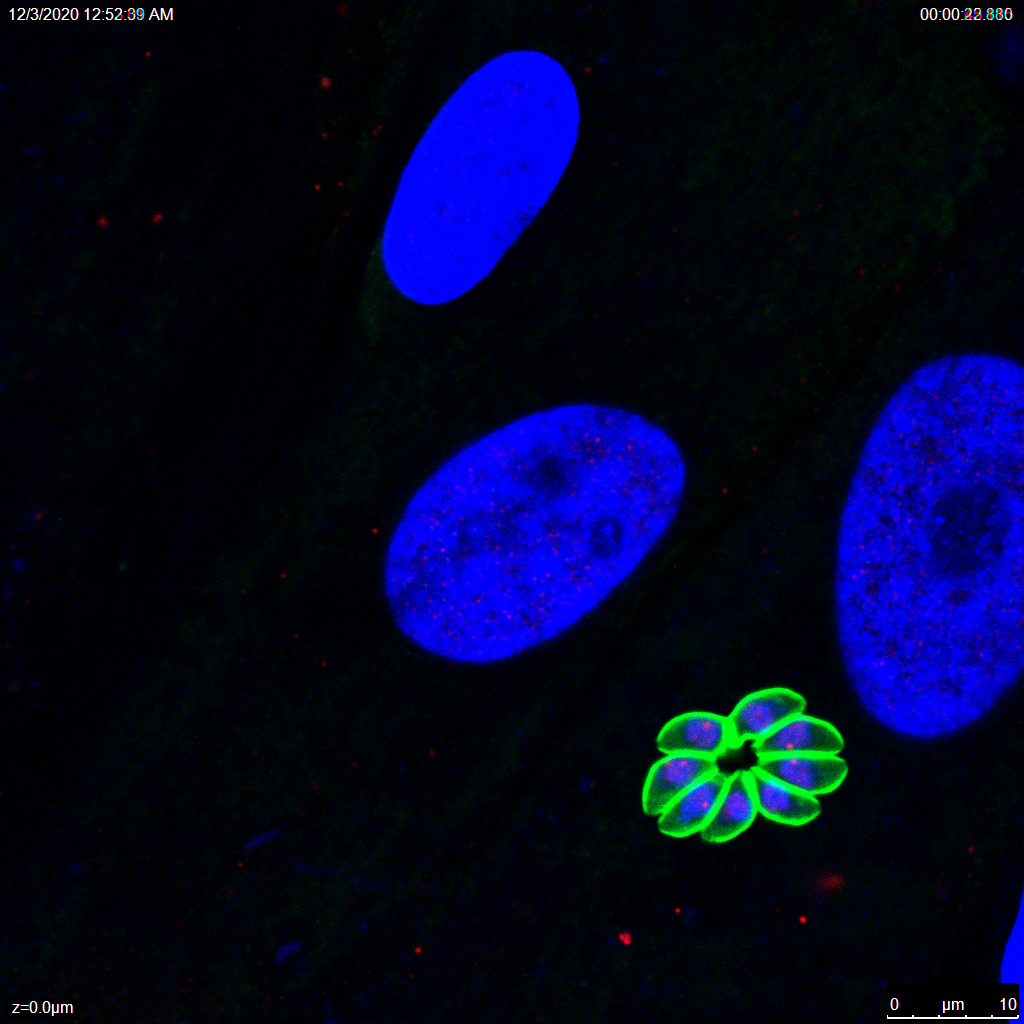

Supplement: Supplementary file 6 [file DataSheet2.ZIP › Figure 2/m4-merge.tif]

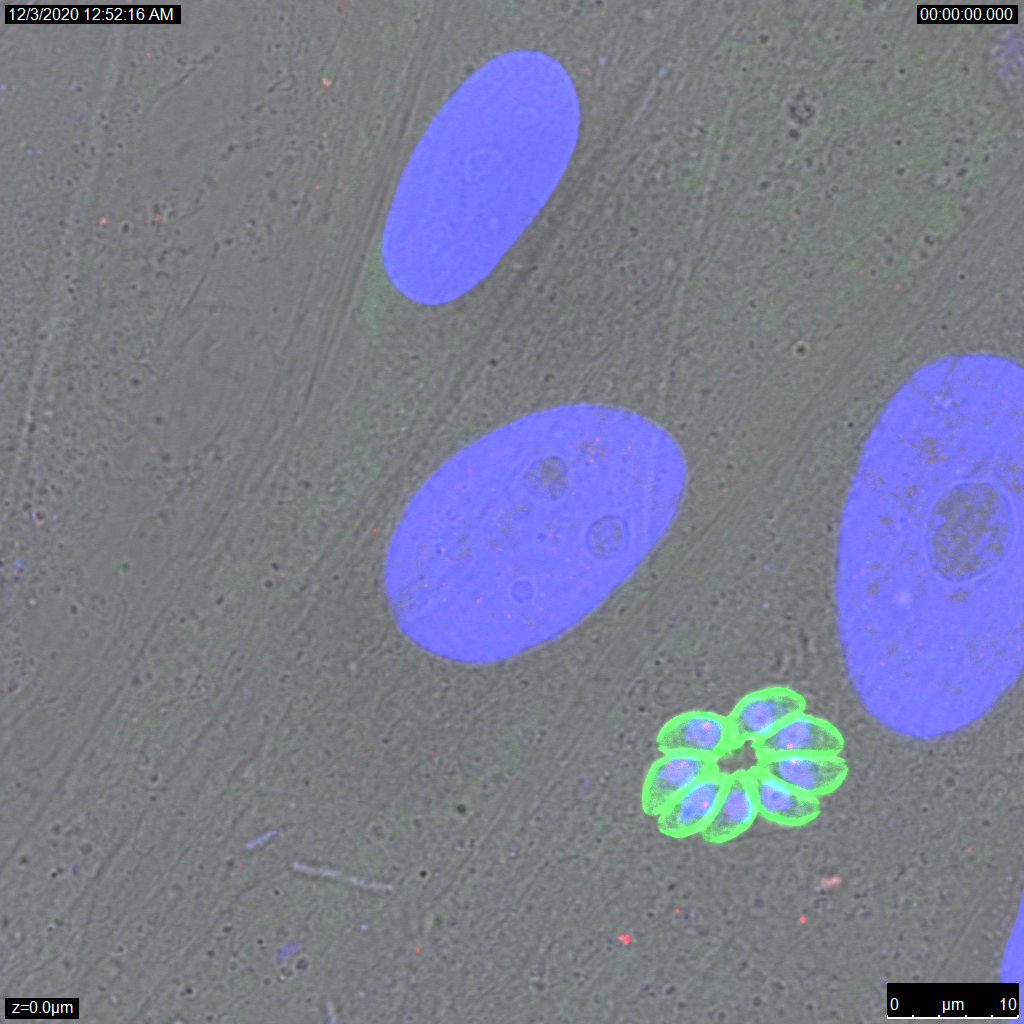

Supplement: Supplementary file 6 [file DataSheet2.ZIP › Figure 2/m4-overlap.png]

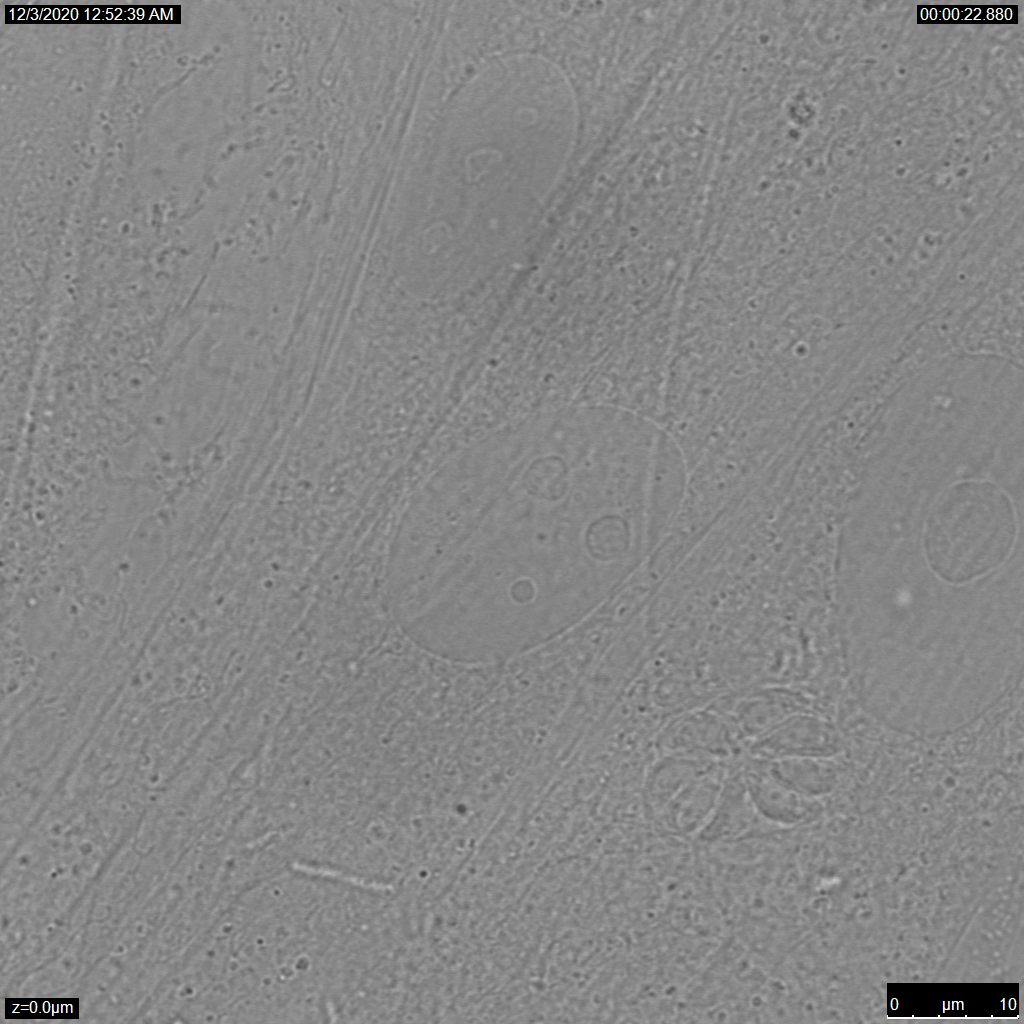

Supplement: Supplementary file 6 [file DataSheet2.ZIP › Figure 2/m4-phase.png]

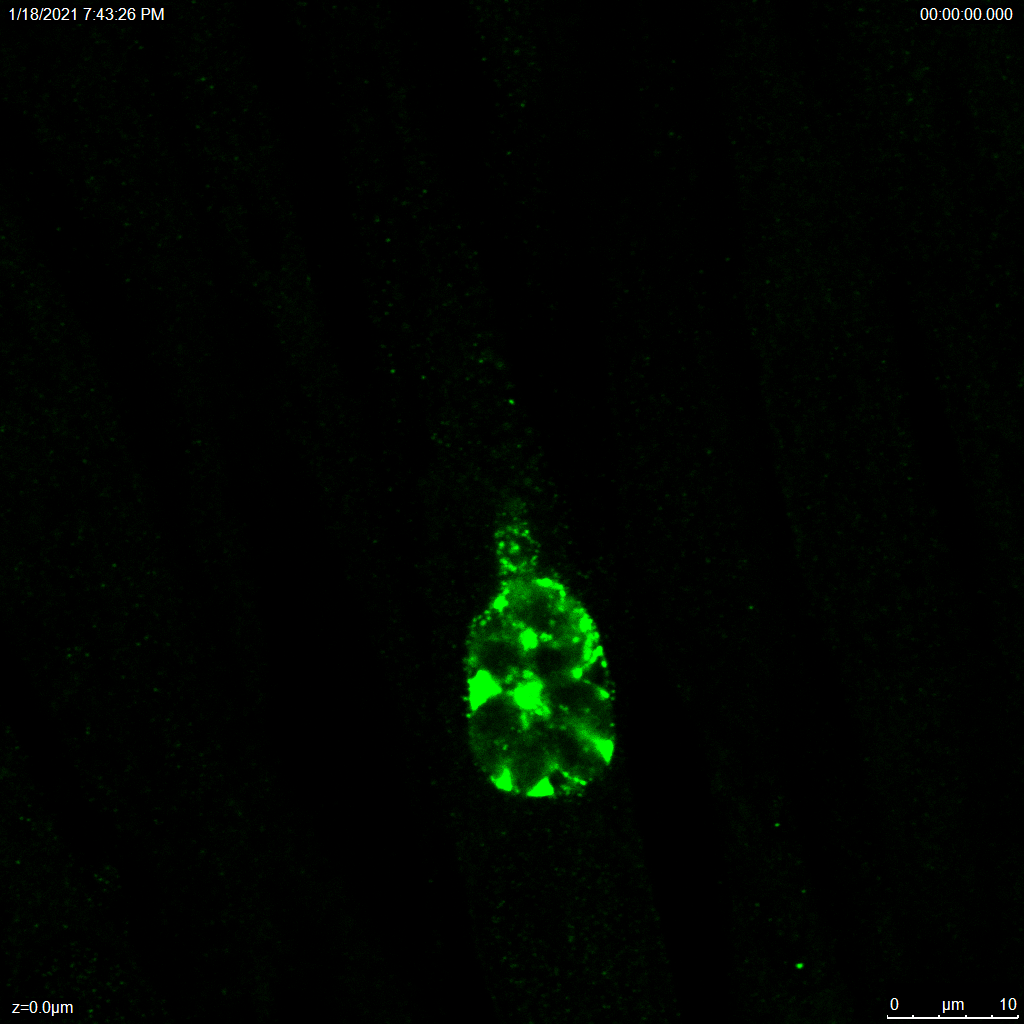

Supplement: Supplementary file 6 [file DataSheet2.ZIP › Figure 2/m8-gra12.png]

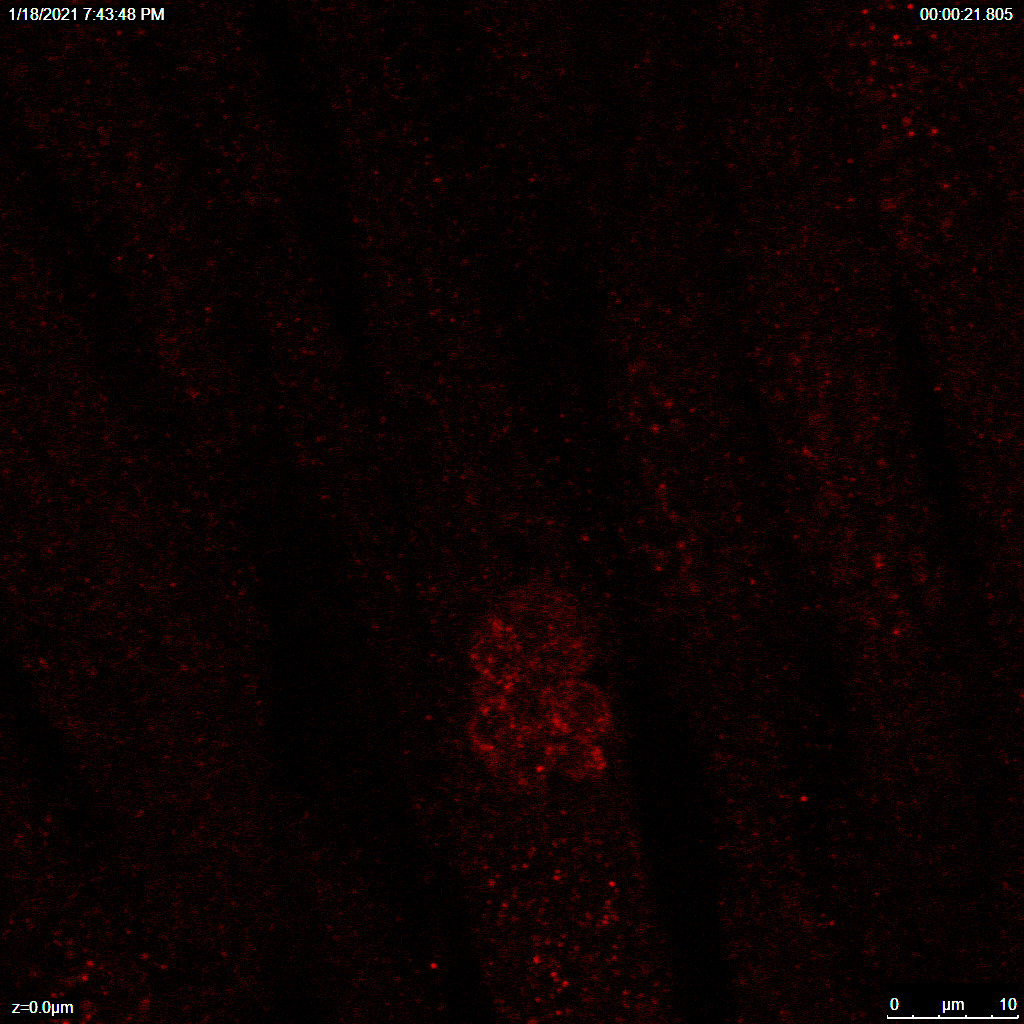

Supplement: Supplementary file 6 [file DataSheet2.ZIP › Figure 2/m8-ha.png]

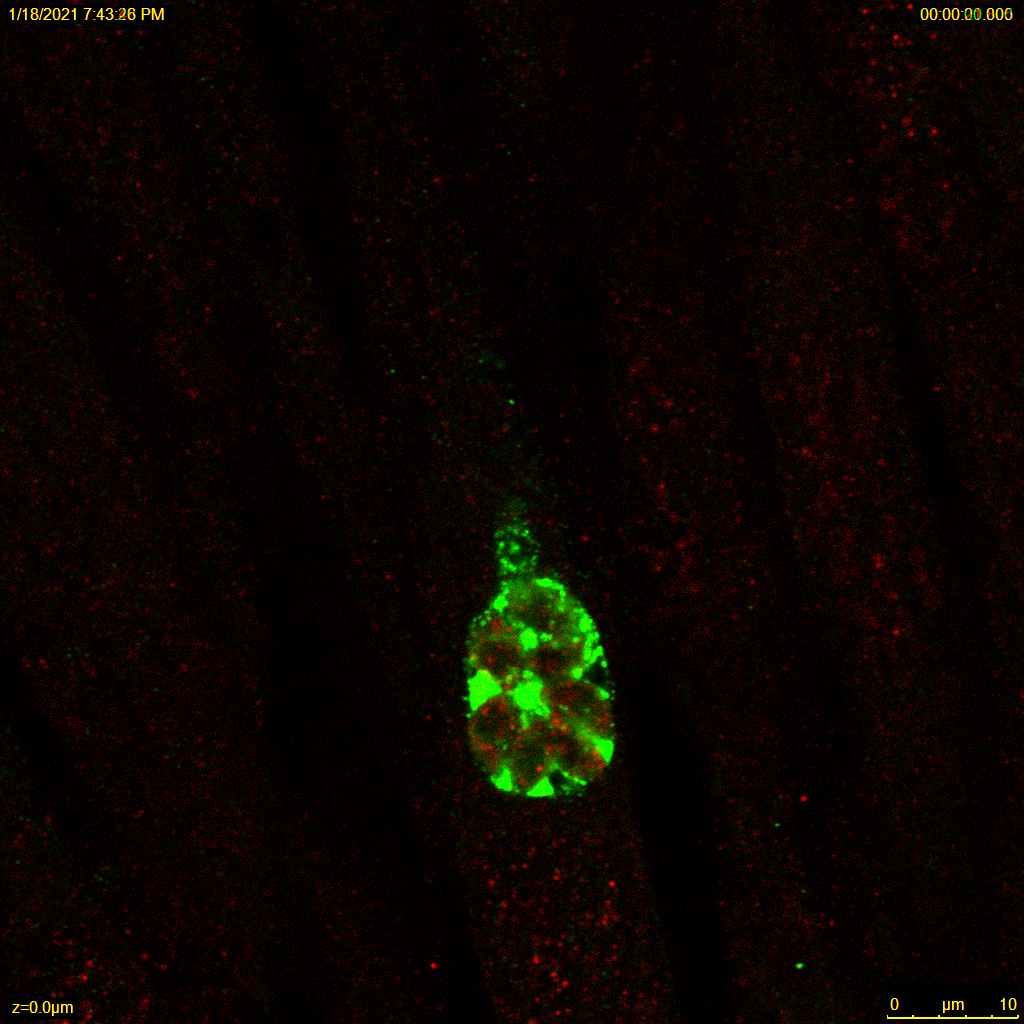

Supplement: Supplementary file 6 [file DataSheet2.ZIP › Figure 2/m8-merge.tif]

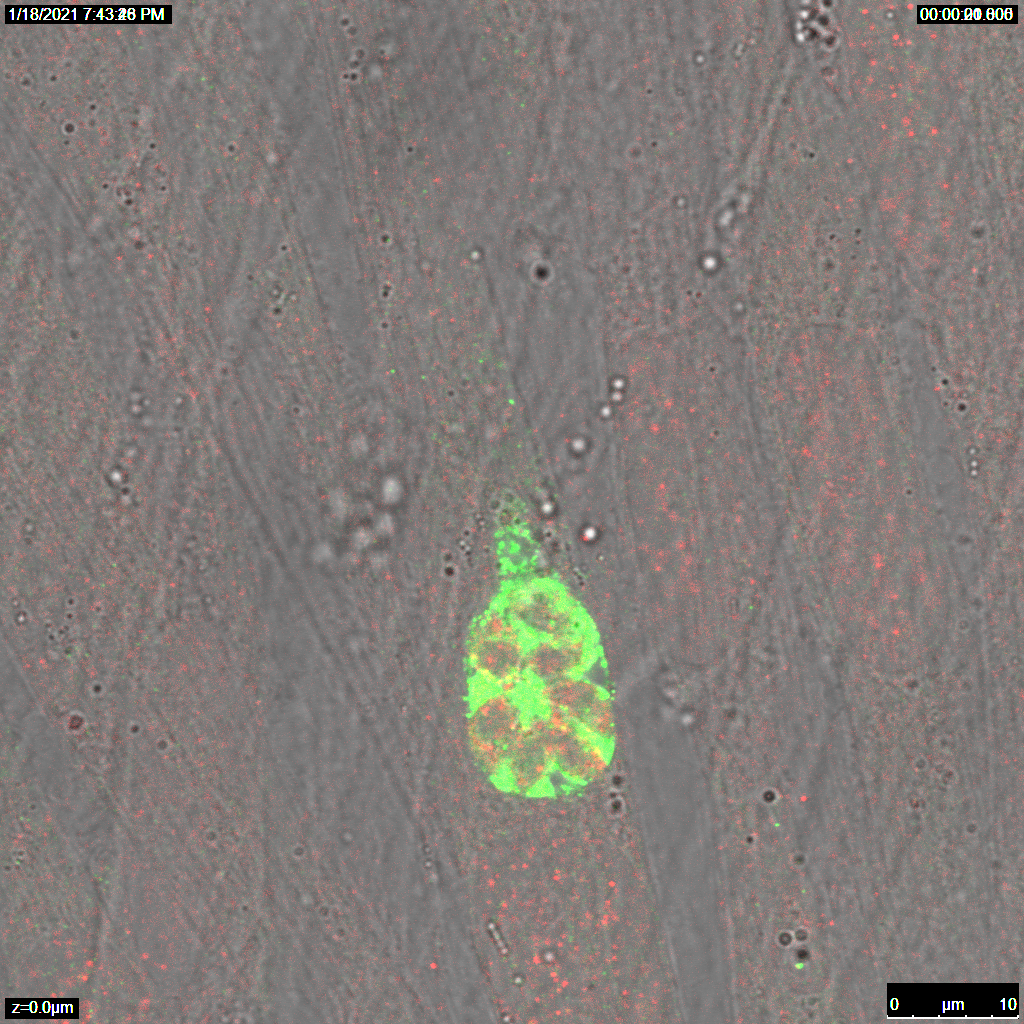

Supplement: Supplementary file 6 [file DataSheet2.ZIP › Figure 2/m8-overlap.tif]

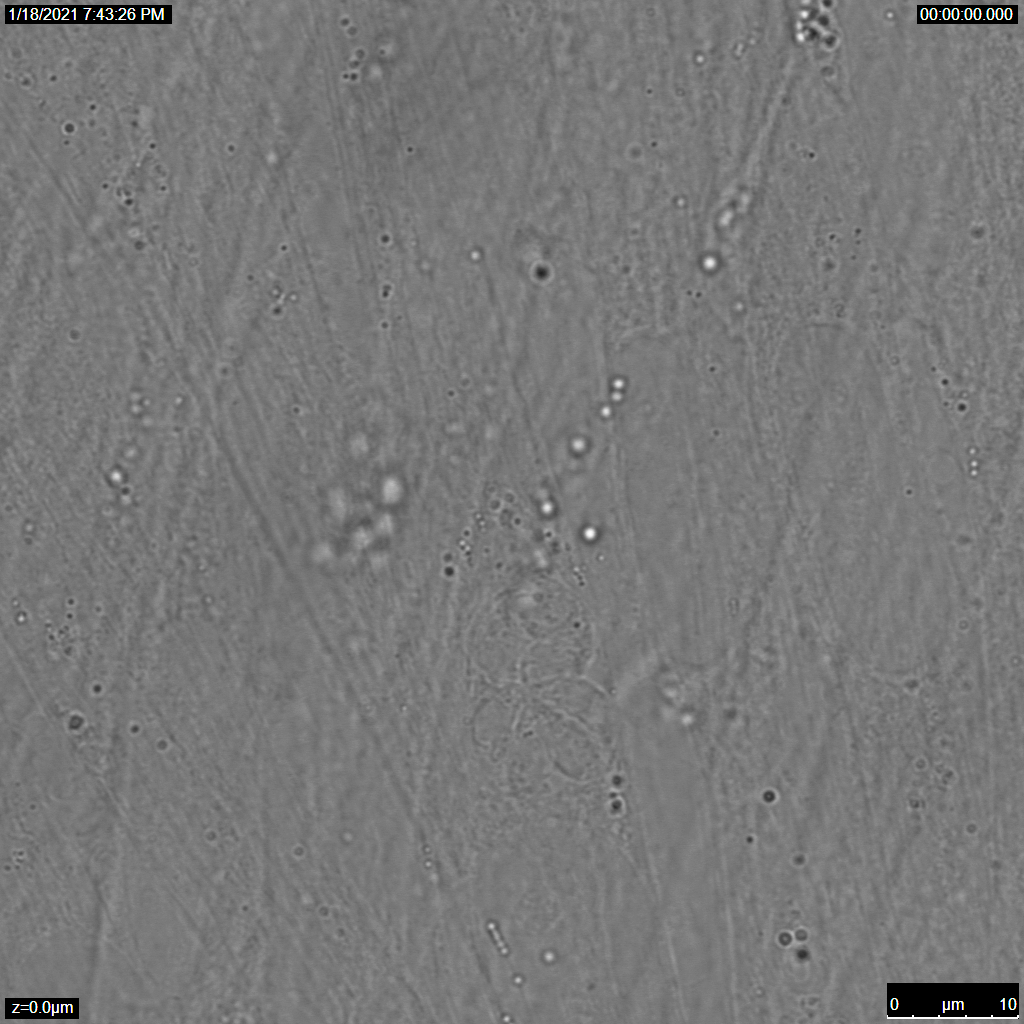

Supplement: Supplementary file 6 [file DataSheet2.ZIP › Figure 2/m8-phase.png]

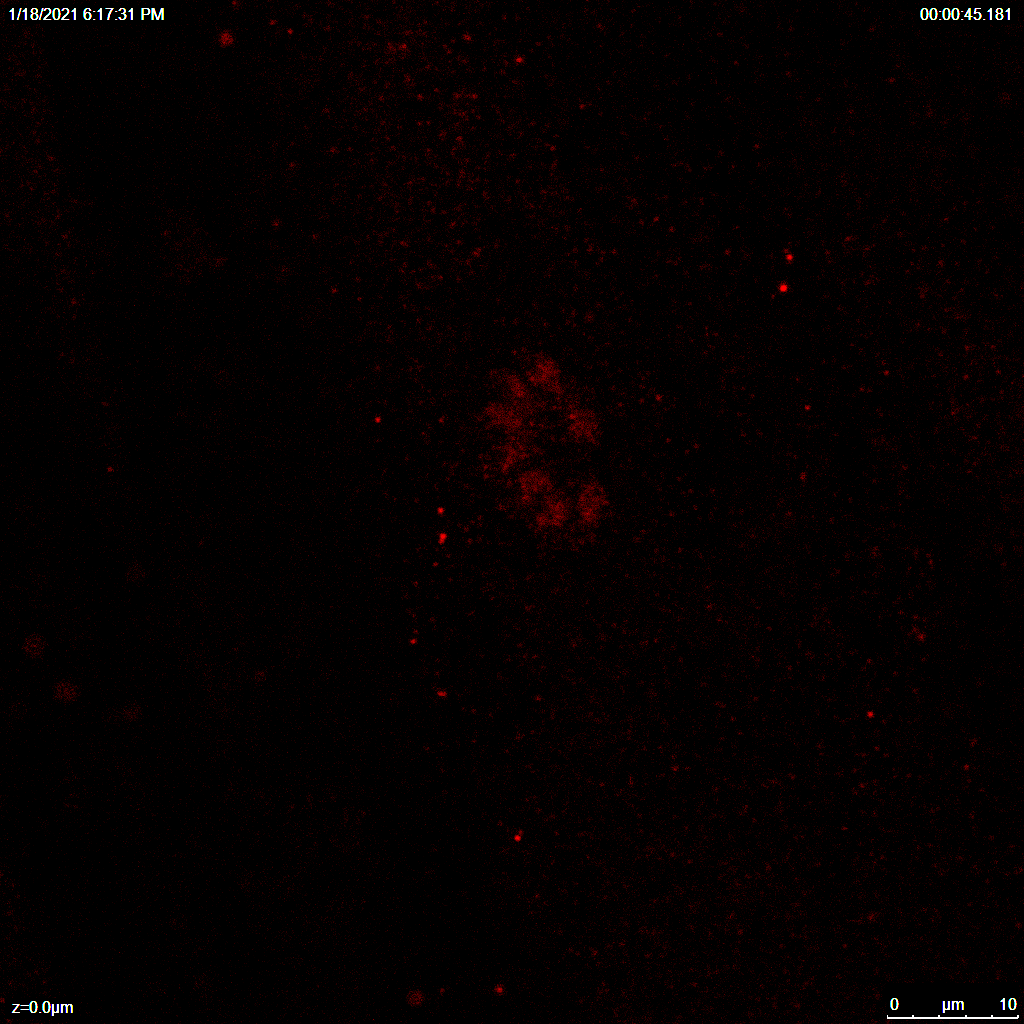

Supplement: Supplementary file 6 [file DataSheet2.ZIP › Figure 2/m9-ha.png]

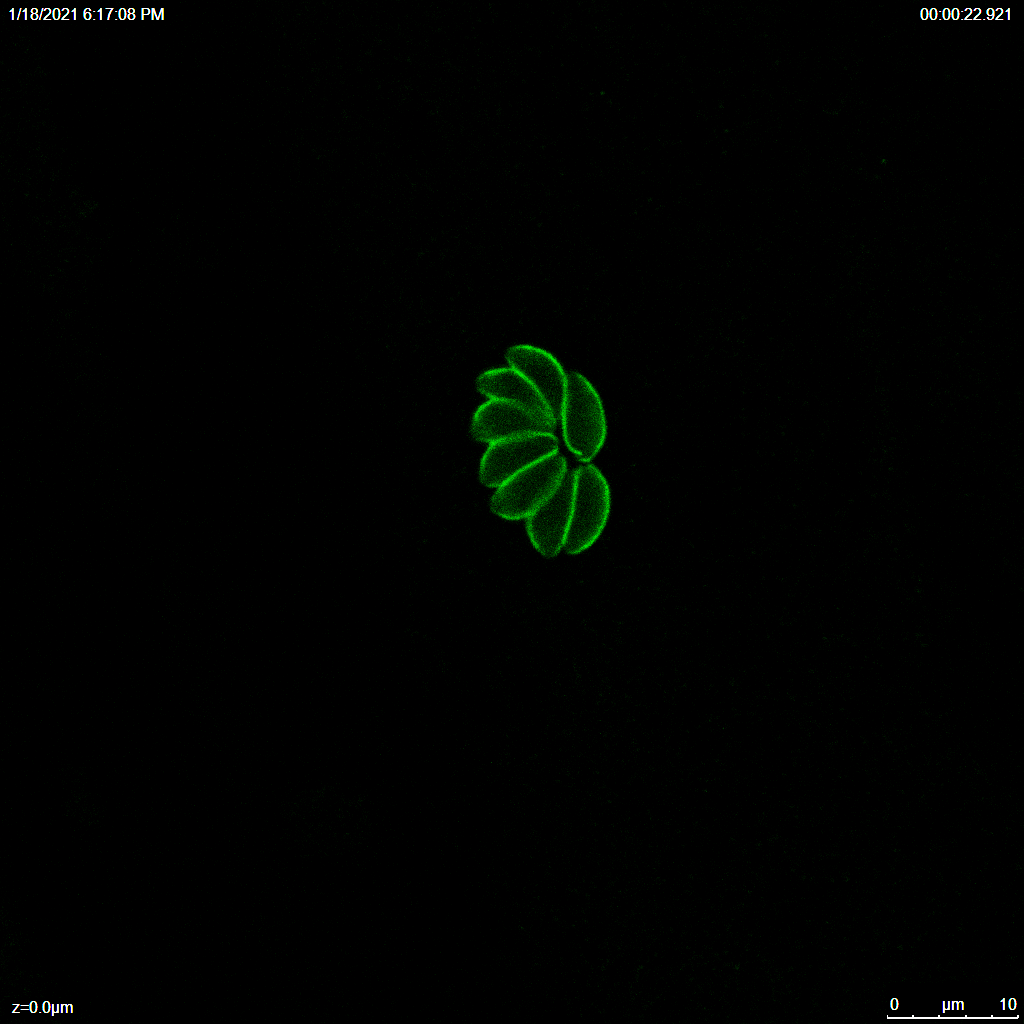

Supplement: Supplementary file 6 [file DataSheet2.ZIP › Figure 2/m9-imc.png]

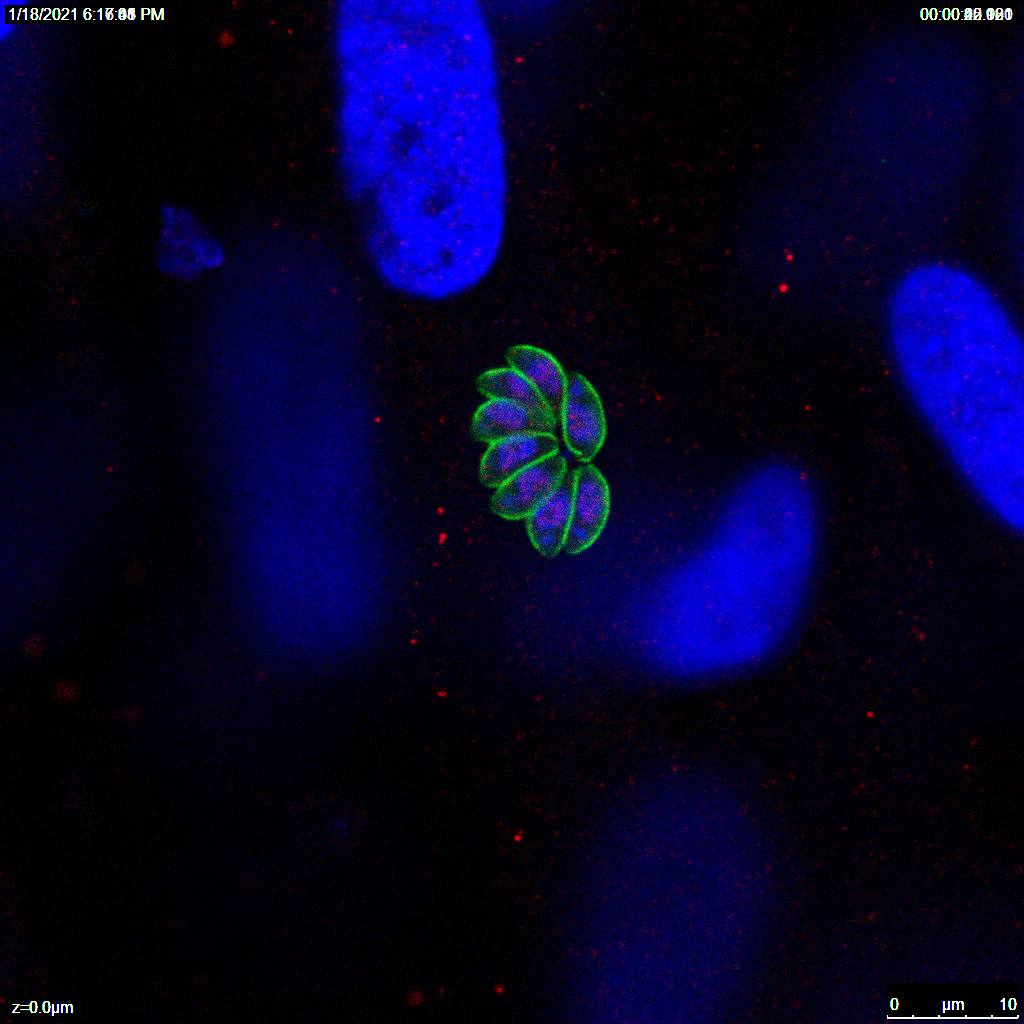

Supplement: Supplementary file 6 [file DataSheet2.ZIP › Figure 2/m9-merge.tif]

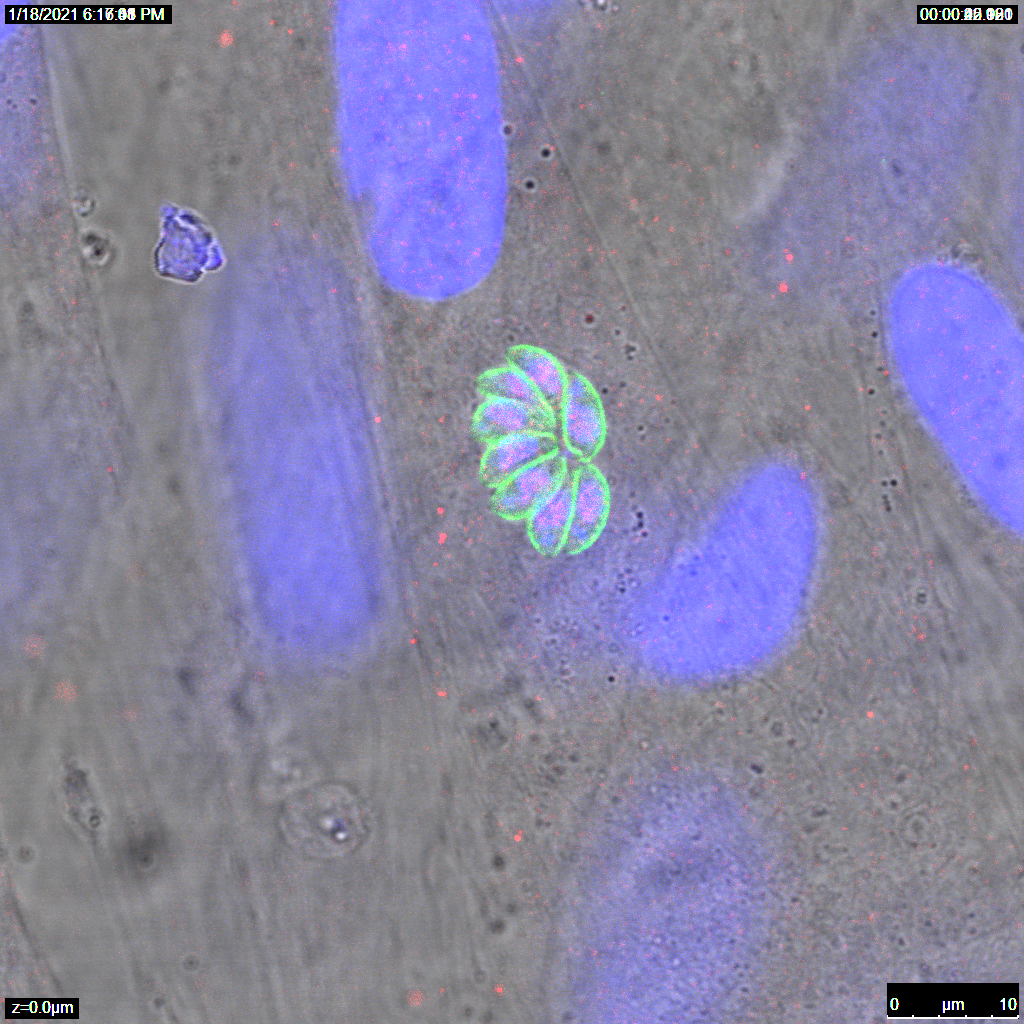

Supplement: Supplementary file 6 [file DataSheet2.ZIP › Figure 2/m9-overlap.tif]

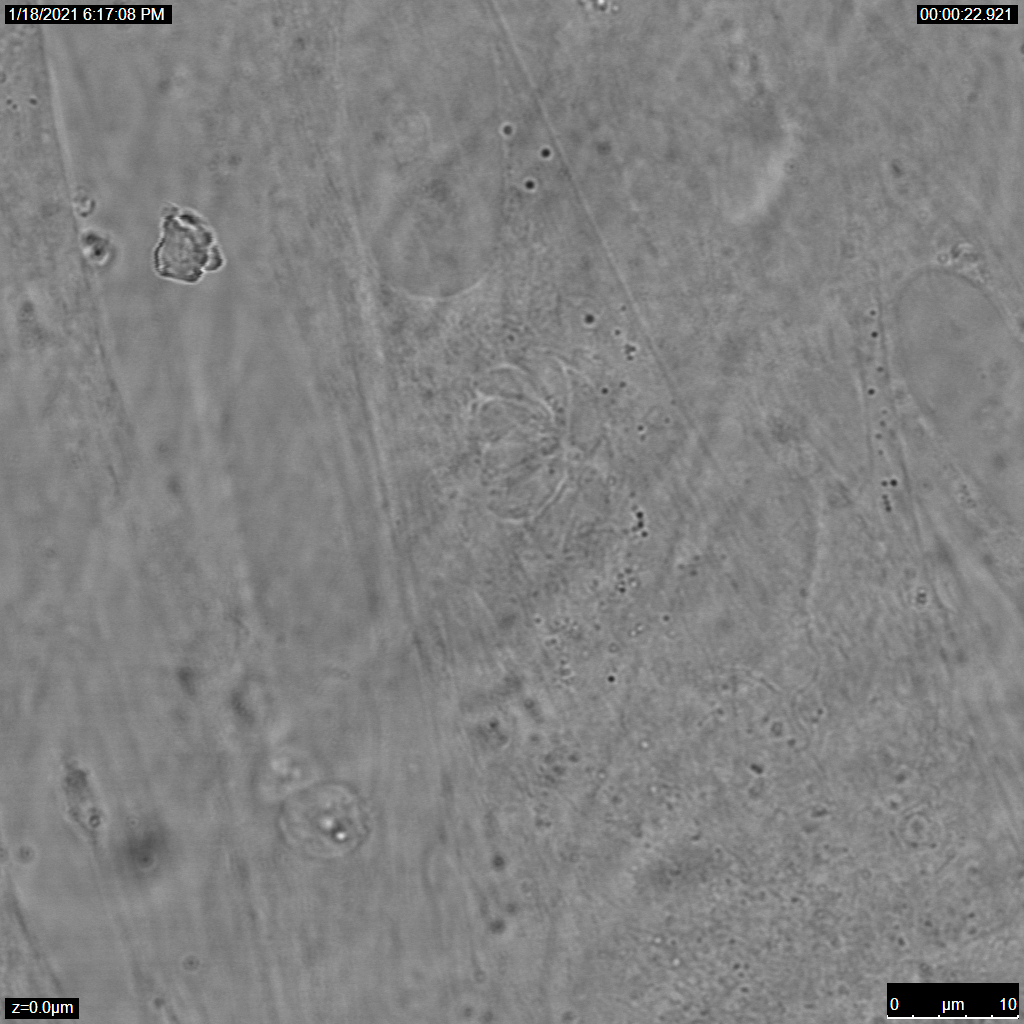

Supplement: Supplementary file 6 [file DataSheet2.ZIP › Figure 2/m9-phase.png]

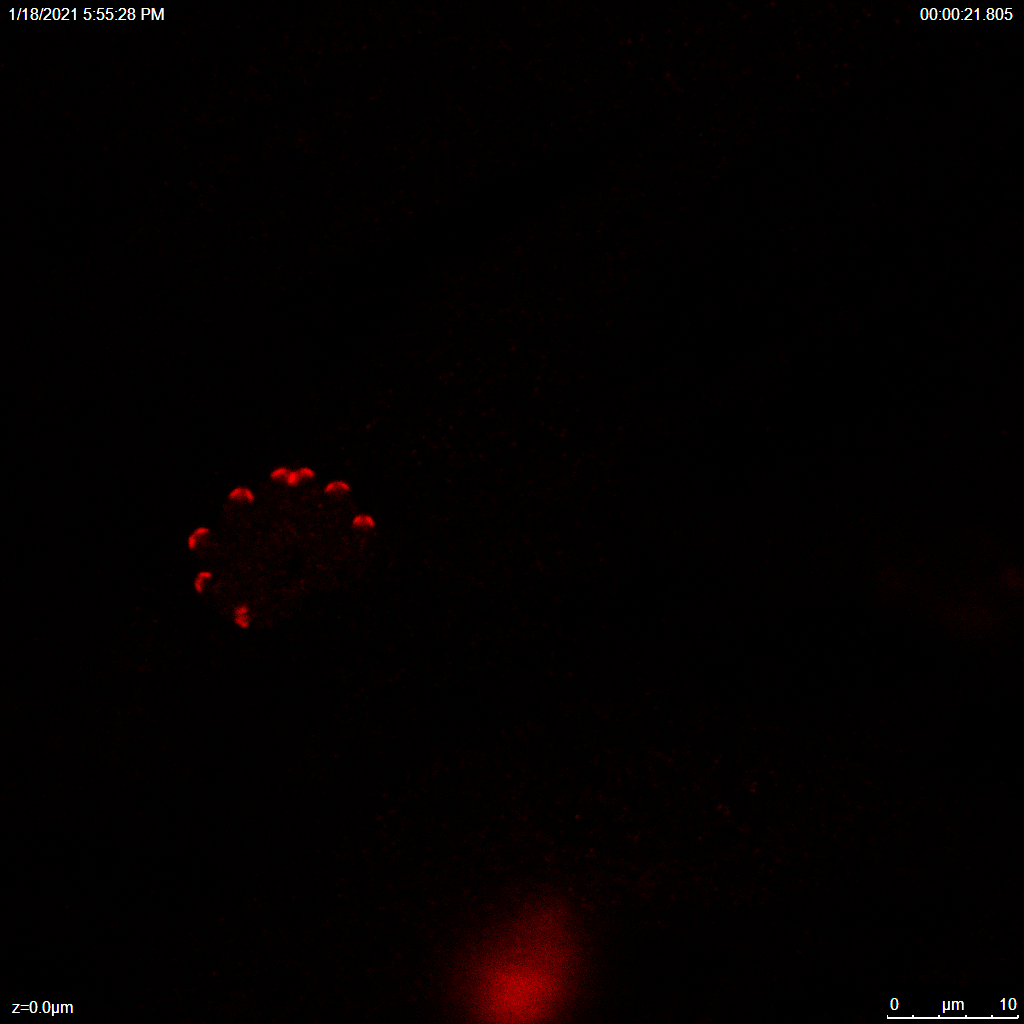

Supplement: Supplementary file 6 [file DataSheet2.ZIP › Figure 2/pp7-ha.png]

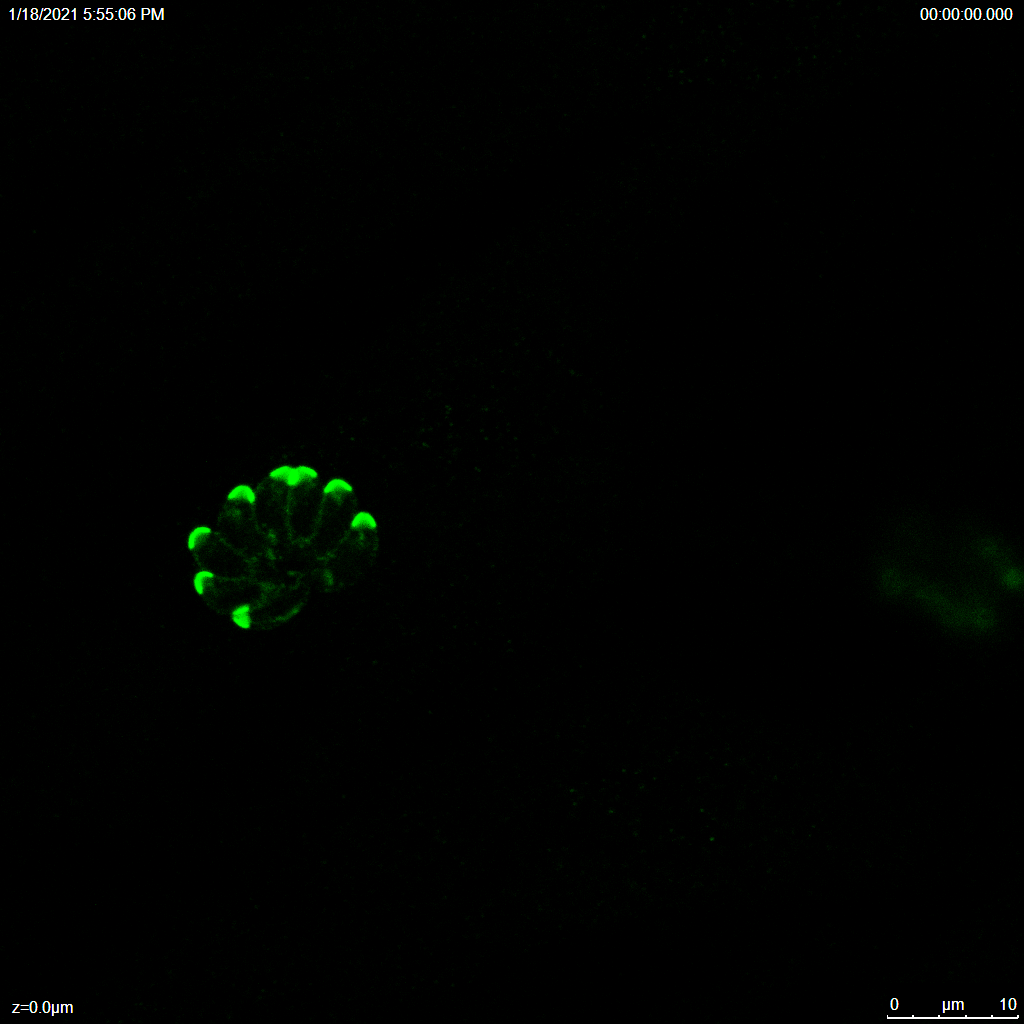

Supplement: Supplementary file 6 [file DataSheet2.ZIP › Figure 2/pp7-ISP1.png]

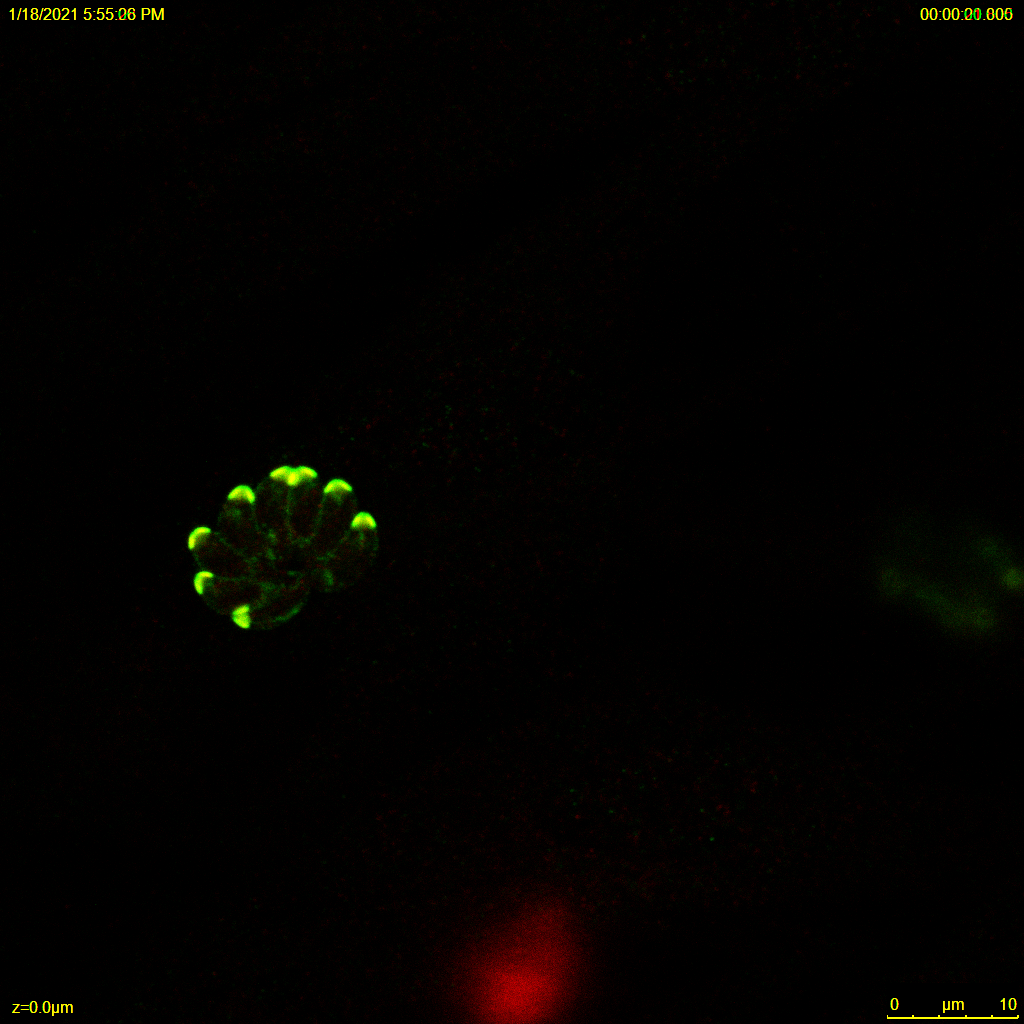

Supplement: Supplementary file 6 [file DataSheet2.ZIP › Figure 2/pp7-merge.tif]

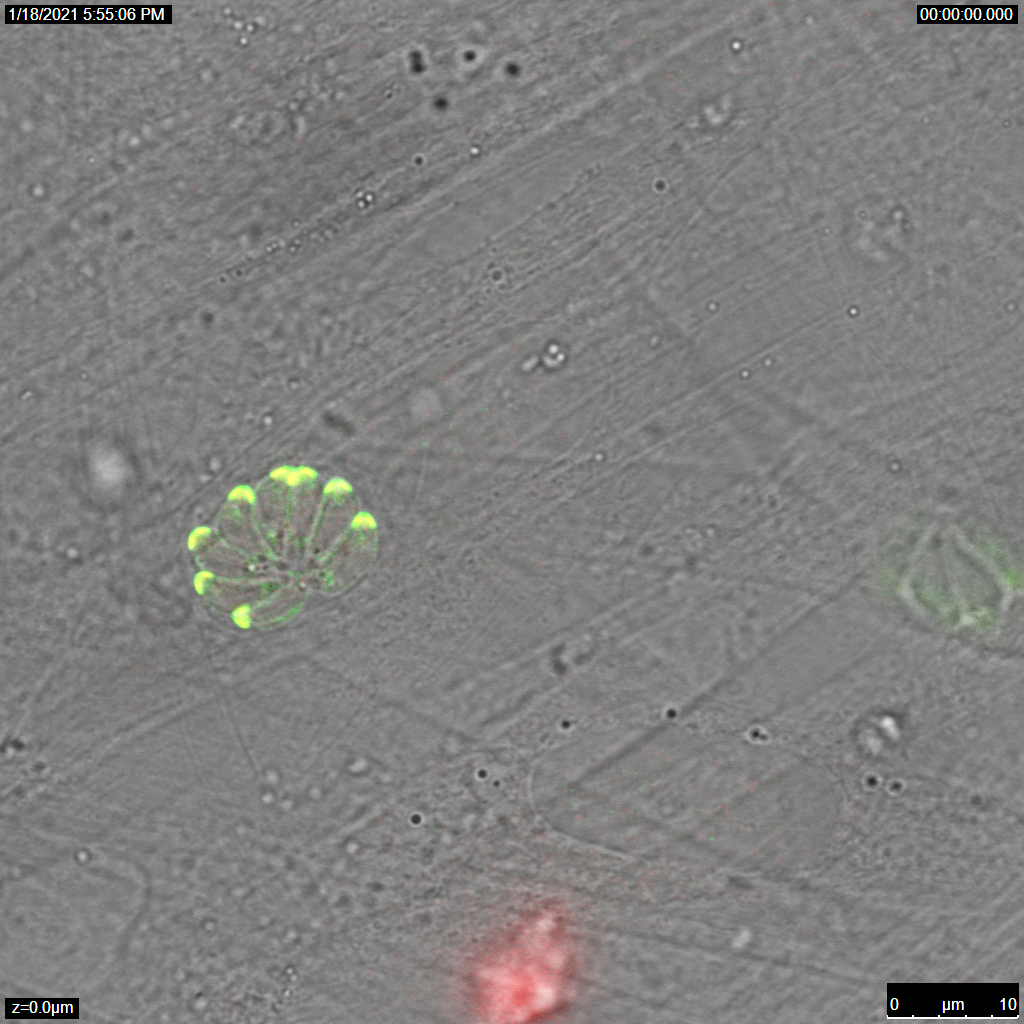

Supplement: Supplementary file 6 [file DataSheet2.ZIP › Figure 2/pp7-overlap.png]

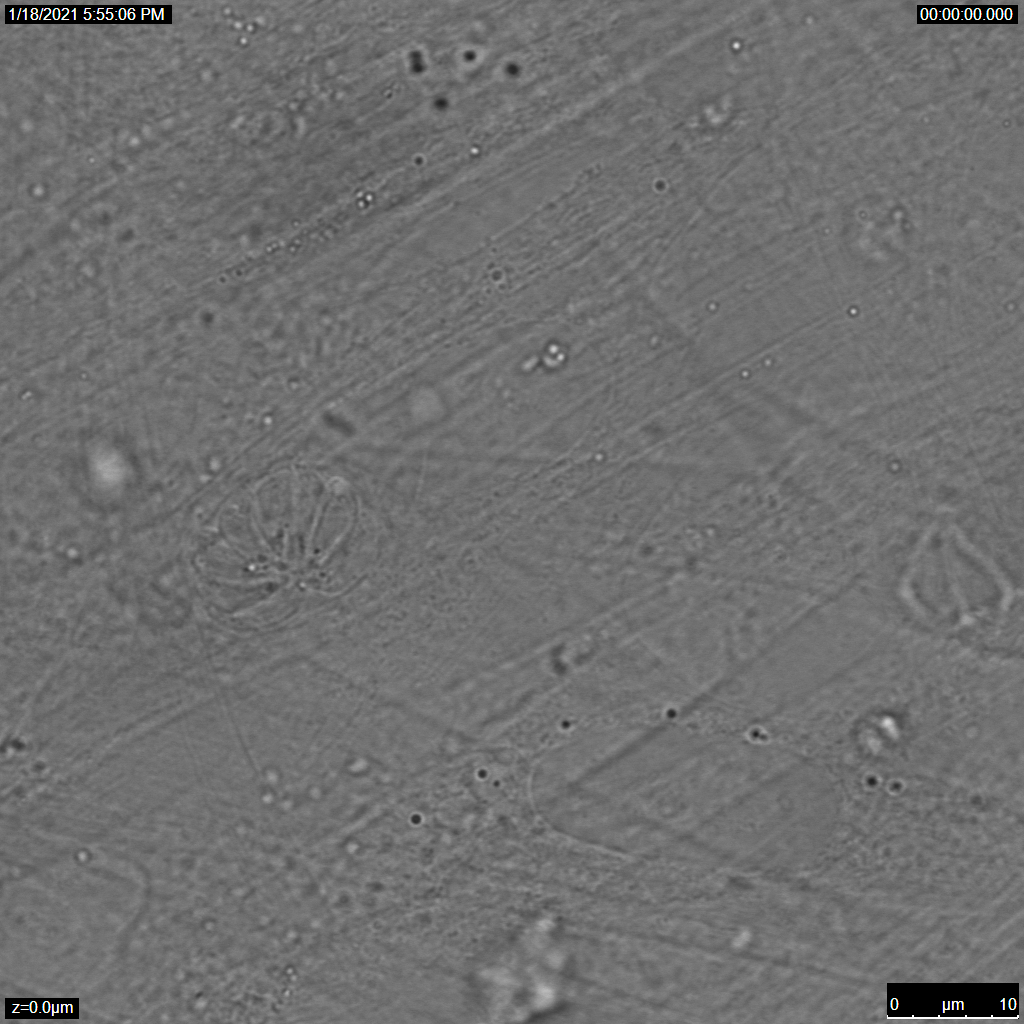

Supplement: Supplementary file 6 [file DataSheet2.ZIP › Figure 2/pp7-phase.png]

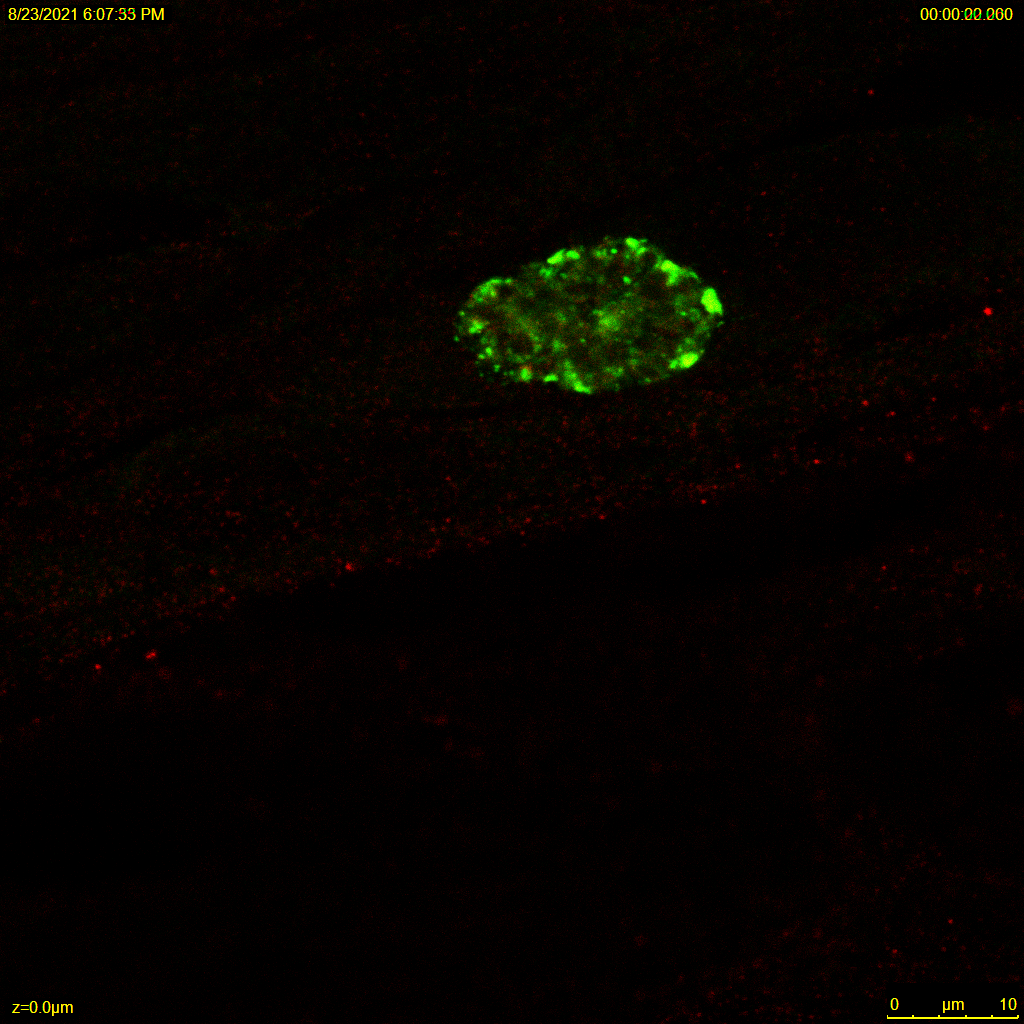

Supplement: Supplementary file 6 [file DataSheet2.ZIP › Figure 2/slp-Composite.tif]

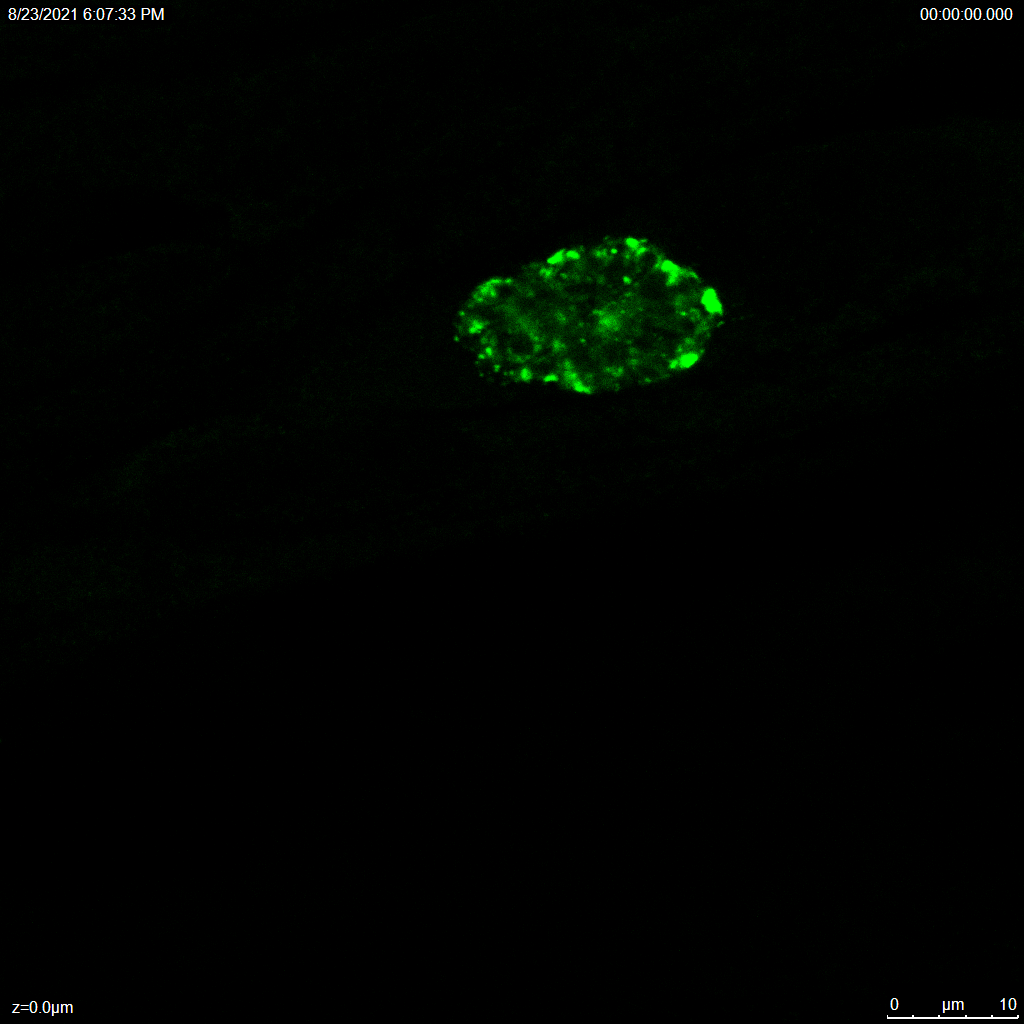

Supplement: Supplementary file 6 [file DataSheet2.ZIP › Figure 2/slp-green.png]
