# Supplementary material for: Functional Characterization of 17 Protein Serine/Threonine Phosphatases in Toxoplasma gondii Using CRISPR-Cas9 System
Source: Front Cell Dev Biol. 2022 Jan 10;9:738794. doi: 10.3389/fcell.2021.738794 (PMC8785970; doi:10.3389/fcell.2021.738794)
Supplement: Supplementary file 4 [file Table1.DOC]

**TABLE S1****｜Details of PSP genes, sgRNAs, identification and 5HR-DHFR-3HR homologous template primers.**

| **Gene** | **sgRNA** | **KO primers (5**′**-3**′**)** | **U5-Gibson-primers (5**′**-3**′**)** | **U3-Gibson-primers (5**′**-3**′**)** |
| --- | --- | --- | --- | --- |
| *PP5* | GGAGTGCTTTGGAGACATGG | F-GGTTCACTGCGGAGACTT | F-CCGATAGGTTCCTGTGTAA | F-TGTAGGTCACCGTCTTCA |
|  | GACGGCGAACGCCCCGGTAG | R-TCGTCAGGAATGCGGATT | R-CTCTCCCGAAACTCCTTC | R-ATTCTGTTCCGCCTCATC |
| *PP7* | GCTGGACGCGTTTGAAGAGA | F-CATTGGCAAGGCGATCTT | F-CCCTCCTCGGCTTTCTAA | F-TCTGTCTGCGTTGCCTAT |
|  | GACGAATGCTGCTCTTATGC | R-CAAGCACCCATCTGTCTAA | R-TACTACCACGCACATCAAC | R-CTCCTCTTCCACCATTCG |
| *EFPP* | GGCCGGGAGGTACTGGCGCG | F-GACAGATGAACCGAACTTAC | F-GGAGACAGAGAGGAAGAAAG | F-CGGAATCCAGTGGTCTCT |
|  | GAAGGACGCGTTGTTTGGCT | R-TGAATGGCACAGACATCC | R-CGGAGCCTGGGATATACA | R-AGGTTCTGCGTTGACAAG |
| *SLP* | GCTTTCGATGAAAGGGGGCA | F-GCGACACTCTCCTGATTC | F-GTATTCGGTAGGAGGTCATC | F-GAGTAAGAGGCAGTTGAATATC |
|  | GCAGGAATCTGGCAATATTG | R-GTCAAGTTCCTCACATATCTTC | R-GGTTCCCGTTTCTCTGTC | R-CTCTCAGGAAGCCAGGAA |
| *PPM3F* | GAAGAGTGGCATCTGGTCAG | F-GAAGAGTGGCATCTGGTCAG | F-CTTGTTCTTGAATGCCTCTT | F-ACATCTTCGCACAACTCTG |
|  |  | R-GGAACAGTGACAAGCCTAA | R-CTTACAGGAGCACTTGGAA | R-GTTCGCTCGCTTCTCTGA |
| *PPM4* | GCTCGGGGACCGTCGCATGA | F-GTCCGTTACTCACCACTG | F-TTCTTCTTCTCTGTCAACGA | F-GAAGGATTCCACGCATCAG |
|  |  | R-GCTCTTACCACTGATGAATT | R-AAGTAGACGCCTTCTCTTG | R-CCGTCACTACTCTCCACTT |
| *PPM5A* | GGATTGATTGCAGCATGAGC | F-GAAGCAGCAACTGTCCATA | F-CTCAAGGTTACCACAGATACA | F-CTTAACTTGGCGTTGTGTAT |
|  |  | R-GCGTTCGGTATCTGTGTC | R-CAAGCAGCAAGTCCTCAG | R-CTCGTTCTGGTCTAGCATAT |
| *PPM5B* | GAAAATCGCGTTTCGGATGG | F-TGATGTCATCGCCAGTTG | F-GTCTGTGAGTTCAACTGCTA | F-TTGATATAGTCAGCCGCTAT |
|  |  | R-CTCGGCTCACTCGTAATC | R-TTCTCTGCCATCCCTGTC | R-CCGCAGTATTCACATCTAAC |
| *PPM6* | GGAGACAGTAGGGCTATCAT | F-AGTGTTGTAGCCGTGTATG | F-AGACCAAGAACGGAGTGT | F-GTTACTTGGAGGTTCATCTTC |
|  |  | R-GGATGTTCAGTACCAAGTTATG | R-TGGCGAACTATGTCAGAGA | R-CAGGAGAATGGACAACAATT |
| *PPM8* | GCAGCCGACGCGTGCGTTCG | F-CTGGATTGGTCGCTAGGA | F-GATAGAGCAGAACCACCAG | F-TTCTTAACGGTGCGATGG |
|  |  | R-GCTGAAGACTCGCATGAA | R-GAGGAGACGAGGAAGAGA | R-GGACTAGATGCTGGATACTG |
| *PPM9* | GCTCTCAGAGCTGGGAGCTG | F-AAGAGGTGTAGATAAGGTCTG | F-CTCTATCTCAGTTCCGTGTC | F-CCGTTATATCGTCCTGCTTA |
|  |  | R-CGGAACTTGGAGAGGAAC | R-TGGCAGACTTCTCAGGAG | R-CCTTACTGTCTCACCAATCT |
| *PPM12* | GCTCCTTCGGAGAAGTTCTG | F-CTGTCTACGATGCGGAAT | F-GGAGTTGCTTGTGAGGAA | F-ACTGTCGTTGTTGGCTAC |
|  |  | R-GTAACATTGCCACTCTTGAA | R-AGTATTCAACACGCCTTAAC | R-GAATAAGCAAGCGGATGTG |
| *PPM14* | GCTGCGTTTGAGACTGTCCG | F-TCTTCTTCGCTTCACCTAC | F-GTTATATGTGATCTGCGTTCC | F-CTTCATCCGACGCAGACT |
|  |  | R-TCCTACGCAGTCCTCATT | R-CCAATCGGAGGTCGTCAT | R-AATAGAAGAGAAGCACAGGAG |
| *PPM18* | CACCGTTCCCTCTTCAAA | F-CACCGTTCCCTCTTCAAA | F-GTAAACCGACCACTCCTG | F-CTGTGAGTCAGGAAGATTCT |
|  |  | R-CGACCTGTCATGTACTACTT | R-AAGTTGCCAGAGTCCATAC | R-GCGACTTCGTCAGAGATAT |
| *CTD1* | GCTGTCTTCGTTATGCGACA | F-GTATGGCTTGGACCTCAC | F-CGAATGACCACGGTAGAG | F-ATCTTCGTGGAGGATAATAGG |
|  |  | R-TTGCTGTGTTGATCTGTGT | R-ATCCAGACGAATGCCTTG | R-CTTAGTGTATGCTTGTCAGTC |
| *CTD2* | GATGGACGAGGCGACGATCT | F-CGAAGCCTCTGAATTAGCA | F-GCTGACAGACTTGGCTTAT | F-AACCAGAGAAGGCTCCTT |
|  |  | R-CACGAACAGCATTGAACAG | R-ACTCCTCGAAGACTATCAATG | R-AGACAAGAAGCAGTGAATCA |
| *CTD3* | GATCTGGCCTGCCCGCGAAG | F-GGAAGAGGGAGAGATGAGA | F-GATCTGCTTCGCTCATAAG | F-GGCGTCAACTCTGATGTT |
|  |  | R-ACGAGAACAGGAGGACAA | R-TGAGAAGATTCCAGGACATC | R-AACTCCTTCTTCTCTTCTTCC |
