# Supplementary material for: Functional Characterization of 17 Protein Serine/Threonine Phosphatases in Toxoplasma gondii Using CRISPR-Cas9 System
Source: Front Cell Dev Biol. 2022 Jan 10;9:738794. doi: 10.3389/fcell.2021.738794 (PMC8785970; doi:10.3389/fcell.2021.738794)
Supplement: Supplementary file 5 [file Table2.DOCX]

**TABLE S2****｜Primers used in the construction of the epitope-tagging strains.**

| **Gene** | **sgRNA** | **KO-Forward (5**′**-3**′**)** | **KO-Reverse (5**′**-3**′**)** |
| --- | --- | --- | --- |
| *PP5* | CGTCTACATGAAGCTGAGCA | AAGCATTGTCCTTCCTACC | ATTCTGTTCCGCCTCATC |
| *PP7* | GTTTTACGCTGATTGCGACA | AGTGTGGCTGTGGTGATT | ACGGAACTCGATTCTCTGT |
| *EFPP* | CGACATCGACTAACCTGCGA | GAATTGTCTCCGCAGTCT | AACGCCCTAAATCCGAAC |
| *SLP* | TATGAGTAGTAGTTGTTGAC | ACGAAGATATGTGAGGAACT | AACTCTCGCTGACTAGGA |
| *PPM3F* | TCTTCAAGTGCTCAAAACAG | ACTCAGTCTGCCTATCCTC | GGTGTCACAGGTGGAATAG |
| *PPM4* | GTAAAGAAAAAAGAGCGGCA | CCTACGAGGCACCTAGTT | AGAGACACAAGCAAGAAGAA |
| *PPM5A* | GTTTTTGCGTATGCATTCAA | AGACTTGACGGTTCATACG | CACGCACGATCCACTTAT |
| *PPM5B* | CCCACGTTTCAGCTCCATCG | ATCCATAGGCGACTCCAT | TGTCGTATCTCCTGTTGTG |
| *PPM6* | GTTTTTCACCCCCGTCCTTT | CTTACGCCTCAAGACGAT | CAGGAGAATGGACAACAATT |
| *PPM8* | TCGTCACCGACGCTCCTTCG | CTTAACGGTGCGATGGTT | CCTACGGTTGTTCAAGAGAT |
| *PPM9* | CAAATTAGTGATCAAACACA | CCTACCAGTTCCTCACTTC | TGTCTCACCAATCTCCAAG |
| *PPM12* | CGGTGTTTGTAGCAGTGTCA | CCGATTGGAGAGTTGGTT | TGAGCAGTAGCGAAGTTC |
| *PPM14* | ACGTGGTCGTATGTTTTTTA | TCTTCTTCGCTTCACCTAC | TCCTACGCAGTCCTCATT |
| *PPM18* | CTTAGGGAATTCATTATTTC | CCGTTCTCGTGATTCCTT | CCATTCCTCGTGTCTTGA |
| *CTD1* | GCGAAACGATCGTTCAAATC | CGAACAGAAGTGTCTCCTC | AGTTACAGCAACGCACAT |
| *CTD2* | GCGGGATCTTTGTGACCTCT | CGTATGAGTAAGGAGTGTGA | AGTCCGACGAAACAAGTG |
| *CTD3* | AGGACGCACAAAGGCGCTGA | TCGCCTCCACATGAAGAA | CTGTCCTCACATTCCTCTG |
