# Supplementary figures and images for: Functional Characterization of 17 Protein Serine/Threonine Phosphatases in Toxoplasma gondii Using CRISPR-Cas9 System (part 2 of 2)
Source: Front Cell Dev Biol. 2022 Jan 10;9:738794. doi: 10.3389/fcell.2021.738794 (PMC8785970; doi:10.3389/fcell.2021.738794)

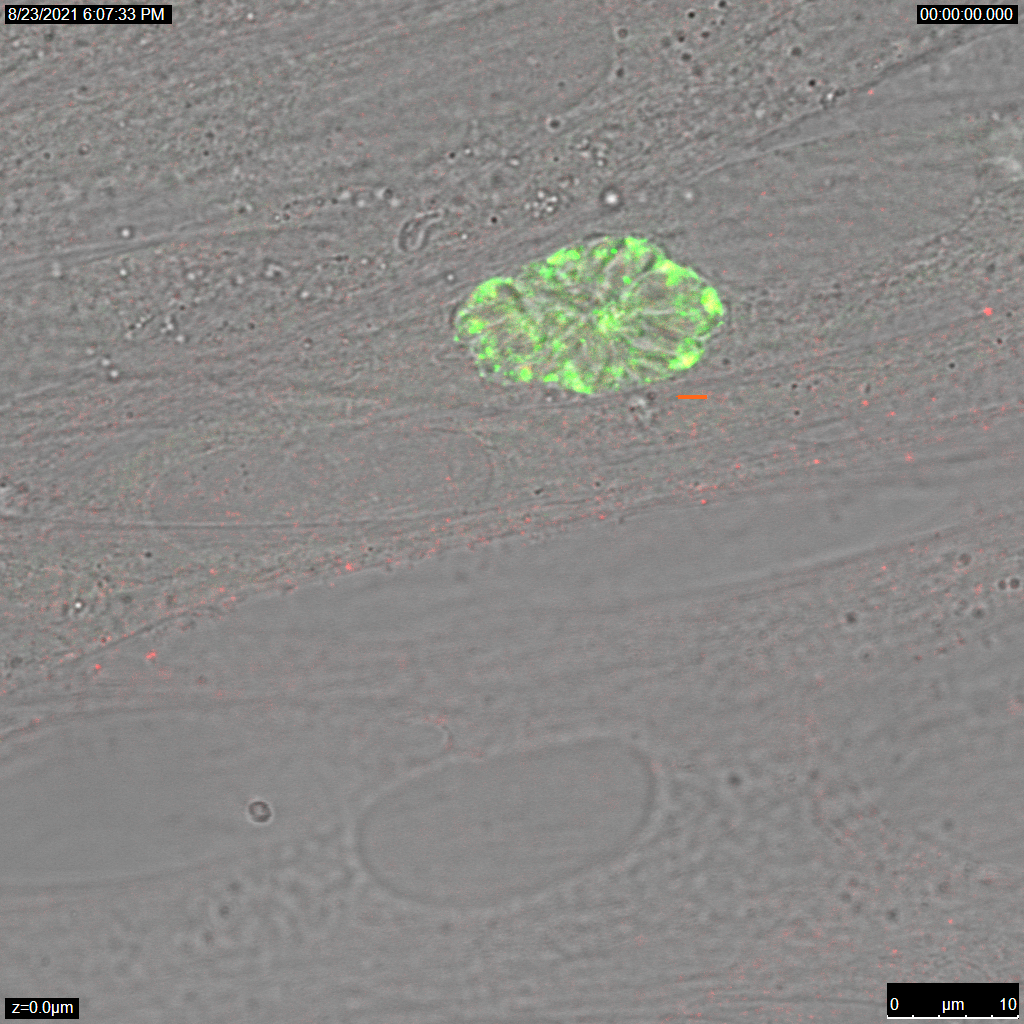

Supplement: Supplementary file 6 [file DataSheet2.ZIP › Figure 2/slp-merge.png]

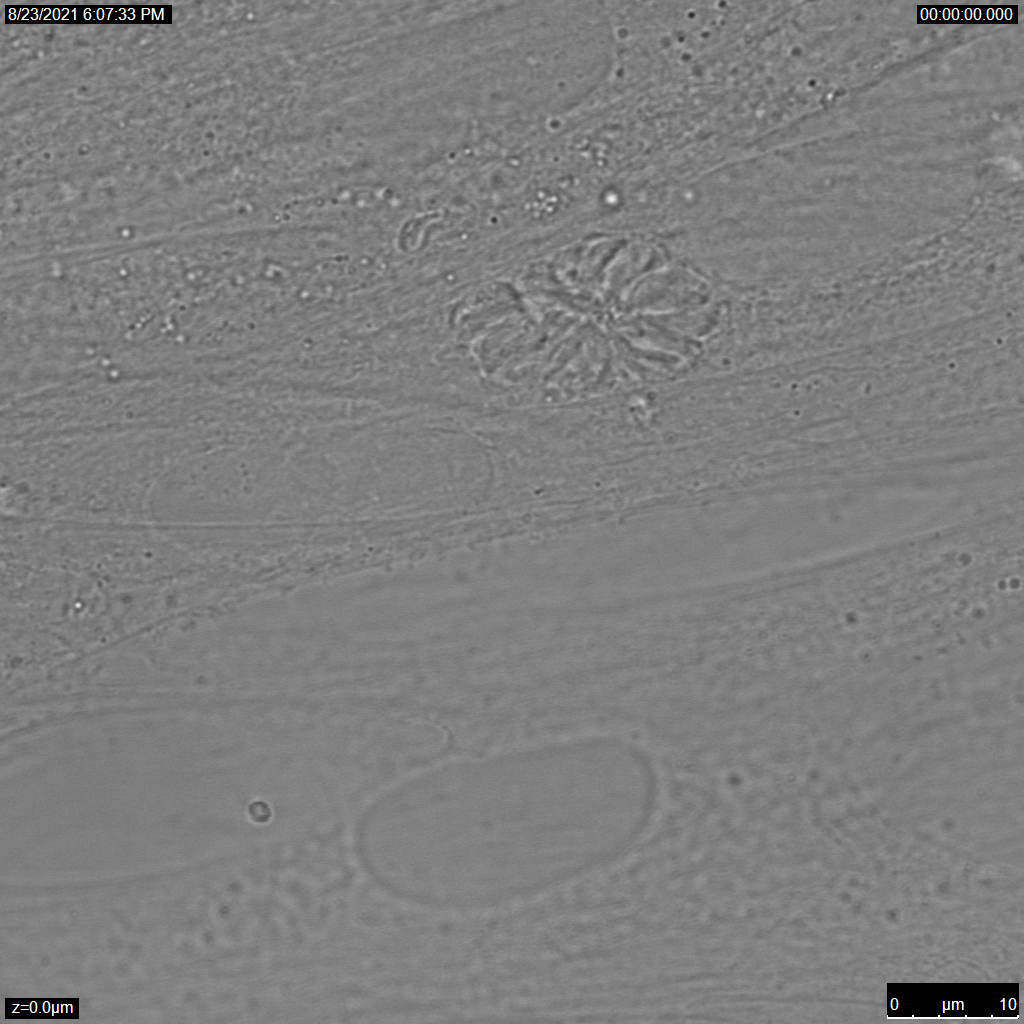

Supplement: Supplementary file 6 [file DataSheet2.ZIP › Figure 2/slp-origin.png]

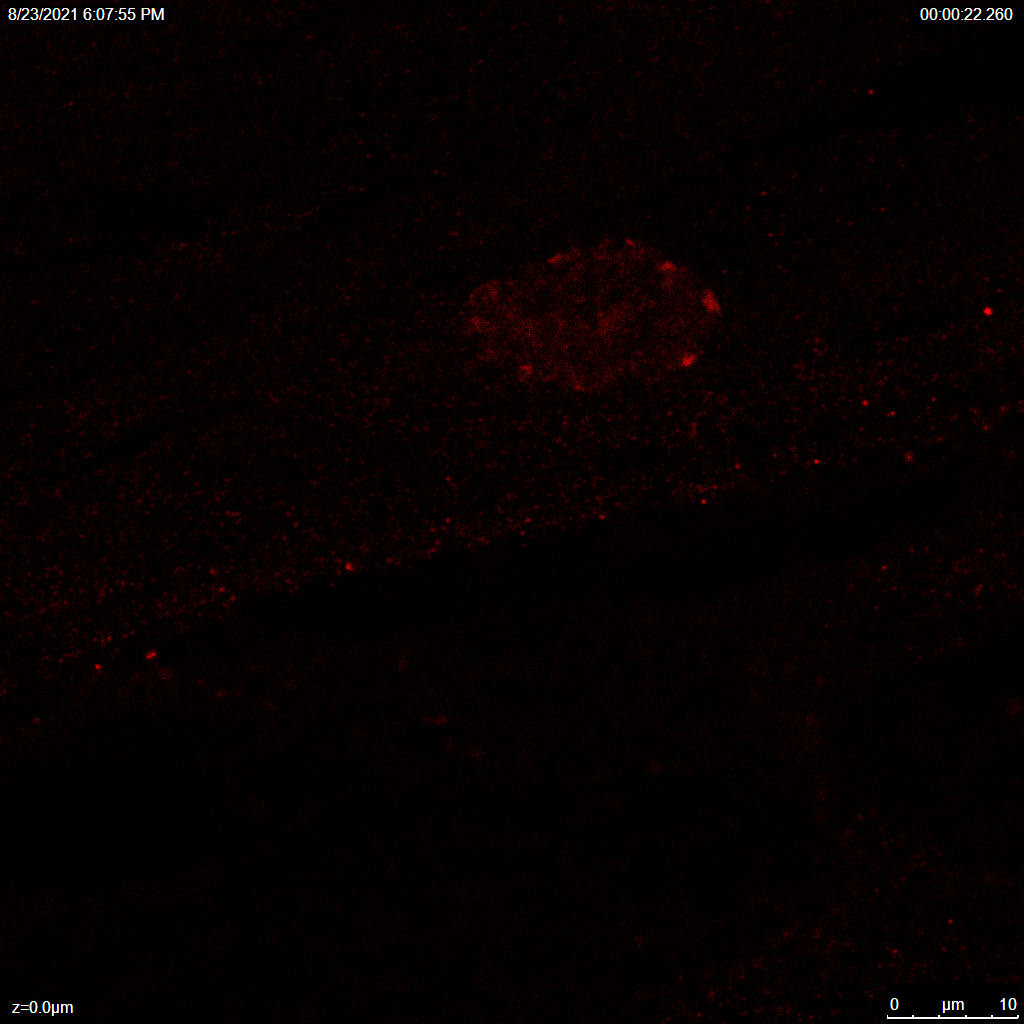

Supplement: Supplementary file 6 [file DataSheet2.ZIP › Figure 2/slp-red.png]
